# Microbiome and ecology of a hot spring-microbialite system on the Trans-Himalayan Plateau

**DOI:** 10.1038/s41598-020-62797-z

**Published:** 2020-04-03

**Authors:** Chayan Roy, Moidu Jameela Rameez, Prabir Kumar Haldar, Aditya Peketi, Nibendu Mondal, Utpal Bakshi, Tarunendu Mapder, Prosenjit Pyne, Svetlana Fernandes, Sabyasachi Bhattacharya, Rimi Roy, Subhrangshu Mandal, William Kenneth O’Neill, Aninda Mazumdar, Subhra Kanti Mukhopadhyay, Ambarish Mukherjee, Ranadhir Chakraborty, John Edward Hallsworth, Wriddhiman Ghosh

**Affiliations:** 1 Department of Microbiology, Bose Institute, P-1/12 CIT Scheme VIIM, Kolkata, 700054 India; 2 Gas Hydrate Research Group, Geological Oceanography, CSIR-National Institute of Oceanography, Dona Paula, Goa, 403004 India; 3 Microbiome Program, Center for Individualized Medicine, Mayo Clinic, Rochester, MN-55905 USA; 4 ARC CoE for Mathematical and Statistical Frontiers, School of Mathematical Sciences, Queensland University of Technology, Brisbane, QLD 4000 Australia; 5 Institute for Global Food Security, School of Biological Sciences, Queen’s University Belfast, 19 Chlorine Gardens, Belfast, BT9 5DL Northern Ireland; 6 Department of Microbiology, University of Burdwan, Burdwan, West Bengal 713104 India; 7 Department of Botany, University of Burdwan, Burdwan, West Bengal 713104 India; 8 Department of Biotechnology, University of North Bengal, Siliguri, West Bengal 734013 India; 9 Present Address: Department of Botany, Jagannath Kishore College, Purulia, 723101 West Bengal India

**Keywords:** Microbial ecology, Biofilms, Metagenomics, Microbiome

## Abstract

Little is known about life in the boron-rich hot springs of Trans-Himalayas. Here, we explore the geomicrobiology of a 4438-m-high spring which emanates ~70 **°**C-water from a boratic microbialite called *Shivlinga*. Due to low atmospheric pressure, the vent-water is close to boiling point so can entropically destabilize biomacromolecular systems. Starting from the vent, *Shivlinga*’s geomicrobiology was revealed along the thermal gradients of an outflow-channel and a progressively-drying mineral matrix that has no running water; ecosystem constraints were then considered in relation to those of entropically comparable environments. The spring-water chemistry and sinter mineralogy were dominated by borates, sodium, thiosulfate, sulfate, sulfite, sulfide, bicarbonate, and other macromolecule-stabilizing (kosmotropic) substances. Microbial diversity was high along both of the hydrothermal gradients. Bacteria, Eukarya and Archaea constituted >98%, ~1% and <1% of *Shivlinga*’s microbiome, respectively. Temperature constrained the biodiversity at ~50 °C and ~60 °C, but not below 46 °C. Along each thermal gradient, in the vent-to-apron trajectory, communities were dominated by *Aquificae*/*Deinococcus*-*Thermus*, then *Chlorobi*/*Chloroflexi*/Cyanobacteria, and finally *Bacteroidetes*/*Proteobacteria*/*Firmicutes*. Interestingly, sites of >45 °C were inhabited by phylogenetic relatives of taxa for which laboratory growth is not known at >45 °C. *Shivlinga*’s geomicrobiology highlights the possibility that the system’s kosmotrope-dominated chemistry mitigates against the biomacromolecule-disordering effects of its thermal water.

## Introduction

The microbial ecologies of habitats that are hydrothermal, or hypersaline, have been well-characterized, and can give insights into the origins of early life on Earth^1–3^. Both chaotrope-rich hypersaline brines and high-temperature freshwater systems can entropically disorder the macromolecules of cellular systems, and are in this way analogous as microbial habitats^4–7^. Indeed, highly-chaotropic and hydrothermal habitats are comparable at various scales of biology: the biomacromolecule, cellular system, and functional ecosystem^8,9^.

Chaotropic, hypersaline habitats include the MgCl_2_-constrained ecosystems located at the interfaces of some of the stratified deep-sea hypersaline brines and their overlying seawater. Biophysical, culture-based, and metagenomic studies of the steep haloclines found at these interfaces have revealed that macromolecule-disordering (chaotropic) activities of MgCl_2_ not only determine microbial community composition, but also limit Earth’s functional biosphere^5,7,10^ in such locations, as *in situ* microbial communities stop functioning at 2.2–2.4 M MgCl_2_ concentrations in the absence of any compensating ion; above these concentrations there is no evidence of cellular functions or life processes^5^. While the biophysical activities of MgCl_2_ and other chaotropic solutes and hydrophobes constrain the functionality of biomacromolecular systems (via mechanisms that are entropically analogous to the action of heat^8,11,12^), a number of macromolecule-ordering (kosmotropic) solutes such as NaCl, proline, trehalose and ammonium sulfate, as well as low temperature, can stabilize, and impart rigidity to, biomacromolecules^11,13–16^, and thereby mitigate against the inhibition of cellular systems by chaotropic agents^5,7,17,18^. Accordingly, active microbial life can be found even in hypersaline brines of up to 2.50–3.03 M MgCl_2_ when sodium or sulfate ions are also present^7,10^. Likewise, for other macromolecule-disordering agents such as ethanol, diverse types of kosmotropic substance as well as low temperature have been reported to mitigate against the chaotropic stress^17–19^. Whereas chaotropicity typically constrains cellular activity at temperatures >10 °C, chaotropes can promote metabolic activity at lower temperatures, thereby extending the growth windows for microbes under extreme cold^6,19^.

**Figure 1:**
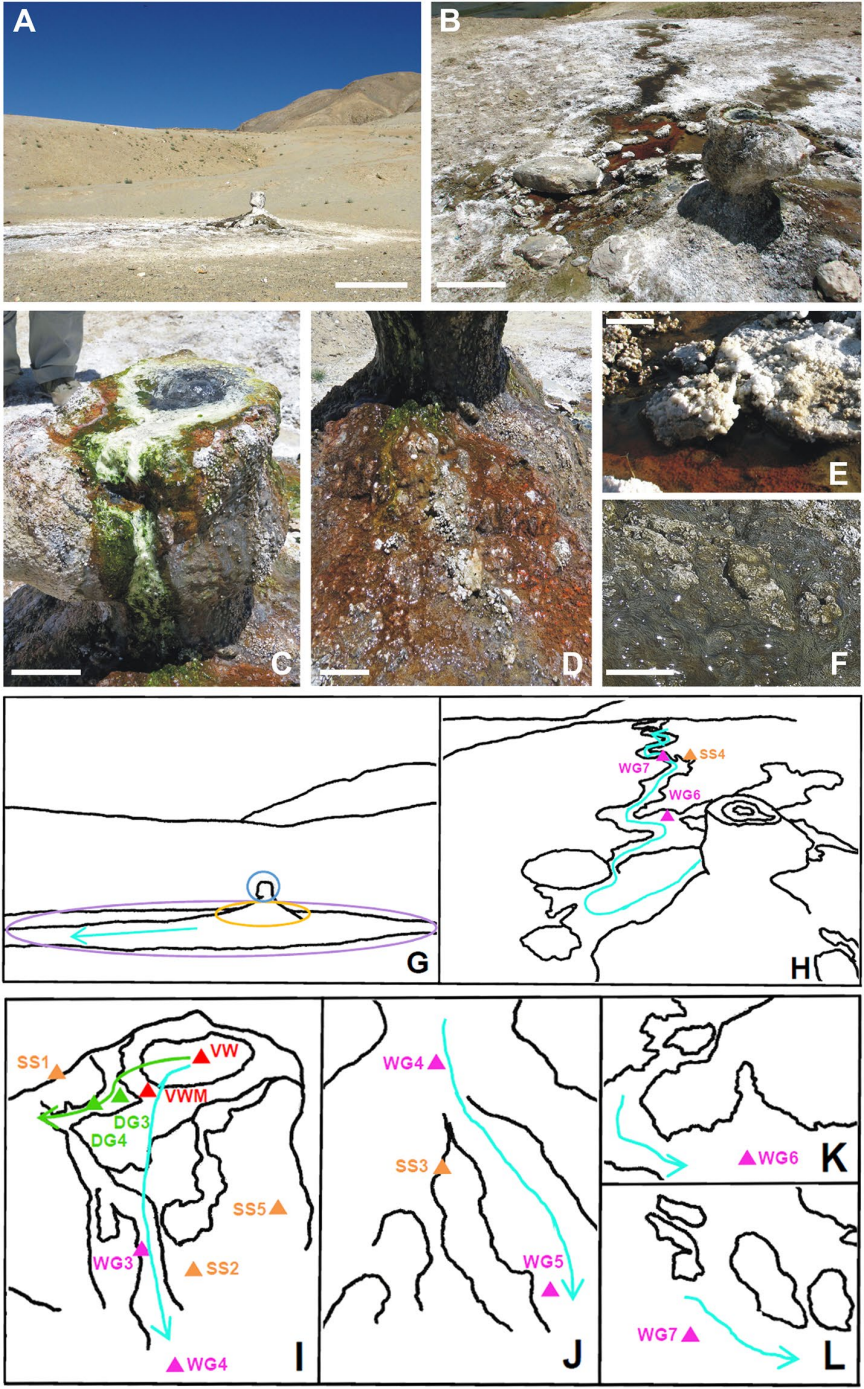
The environmental context of the *Shivlinga* microbialite hot spring system at the time of sampling on 23 July 2013: (**A**) the three geomicrobiological zones, scale bar represents 1 m; (**B**) view of *Shivlinga’s* microbial communities showing the spring-water transit that represented the wet thermal gradient, scale bar represents 1 m; (**C**) microbialite body showing the vent and position of the two thermal gradients that were sampled, scale bar represents 10 cm; (**D**) bedrock slope around *Shivlinga*’s base, showing the microflora, scale bar represents 10 cm; (**E** and **F**) WG6 and WG7, respectively, scale bars represent 5 cm for E and 10 cm for F. For (**G**), the light blue circle indicates the microbialite body, the yellow oval demarcation indicates the bedrock slope around the base, the purple oval demarcation shows *Shivlinga*’s apron, and the cyan arrow shows the direction of water flow across the apron, away from the microbialite hot spring. For (**H**), the cyan curved-line with an arrow-head shows the meandering path of the spring-water across the apron, away from the microbialite body (this also represents part of the wet thermal gradient), the pink triangles indicate the sample sites for WG6 and WG7, the orange triangle indicates the location of the Sinter-Sample 4 (SS4). For (**I**), the green curved-line with an arrow-head indicates the trajectory of the drying thermal gradient, while the cyan curved-line with an arrow-head indicates part of the wet thermal gradient which starts at the vent and leads to the first mat of the bedrock slope (the latter also represents the direction of water flow); the red triangles indicate the position of the sampled communities VW and VWM; the green triangles indicate the sampling-positions for communities DG3 and DG4; the pink triangles indicate the sample sites for WG3 and WG4; and the orange triangles indicate the locations of the Sinter-Samples 1, 2 and 5 (SS1, SS2 and SS5, respectively). For (**J**), the cyan curved-line with an arrow-head indicates part of the wet thermal gradient that runs along the bedrock slope surrounding *Shivlinga*’s base; the pink triangles indicate the sample sites for WG4 and WG5; and the orange triangle indicates the location of the Sinter-Sample 3 (SS3). For (**K**) and (**L**), the pink triangles indicate the sampling positions for communities WG6 and WG7, while the cyan curved-lines with an arrow-head each show the direction of water flow.

Ecological studies of geographically distinct hydrothermal habitats^20–26^ have elucidated various aspects of *in situ* microbiology including diversity^27–31^ and correlation of community structures/functions with temperature^32–39^. However, in relation to conditions which permit and/or constrain habitability, we currently know little about the geochemistry and biophysics of hot spring systems, especially the ones discharging hot waters, which have a neutral pH and are typically poor in sulfide, silicate and total dissolved solids but rich in sodium, boron, elemental sulfur and sulfate^40–42^. The current study, based in the Puga geothermal area of eastern Ladakh (within the Trans-Himalayan region, at the northern tip of India; see Supplementary Fig. 1 and Supplementary Note 1), focused on revealing the geomicrobiology of a sulfate- and boron-rich, silica-poor and neutral pH, hot spring originating from within a large chimney-shaped hydrothermal microbialite, known as *Shivlinga* (Fig. 1). This microbialite, which has formed via epithermal accretion of boratic and carbonatic hydrothermal minerals, is situated at an altitude of 4438 m, where the boiling point of water is approximately 85 °C. The functional ecology of *Shivlinga*’s microbiome was elucidated via a sampling-microscopy-geochemistry-metagenomics-biophysics approach, which encompassed analyses of water from the vent, microbial mats along the spring-water transit/dissipation, and fresh mineral sinters precipitating on mat-portions growing near the margins of the water-flow. Microbial diversity, microbe-mineral assemblages, and geochemical and biophysical parameters were characterized along *Shivlinga*’s hydrothermal gradients (Fig. 1) to determine the factors constraining and/or promoting the microbiome. The chemical milieus of these hydrothermal gradients were found to be rich in kosmotropic ions, so we compared their ecology with those of the kosmotrope-compensated chaotropic haloclines.

## Results

### The boratic microbialite, *Shivlinga*

*Shivlinga* is a 35-cm-tall microbialite that is several decades old according to residents of the closest village situated at the eastern end of the Puga valley (Fig. 1A,G). The abundance of microbial biomass on its surface and chemical composition of its mineral body (see below) suggest that *Shivlinga* has formed via precipitation of boratic minerals on lithifying microbial mats. Laminated, clotted or dendritic fabrics commonly found on stromatolites, thrombolites or dendrolites^43^ were not encountered on *Shivlinga*’s mineral deposits. It is for this reason that *Shivlinga* was considered to be a microbialite, i.e. a microbe-mediated sedimentary rock-like mineral deposit with or without defined internal fabrics^43^. The near-circular, flat top of *Shivlinga’s* mineral body has a mean diameter of ~22 cm (Fig. 1C,I). Throughout the year, there is a gentle discharge of water (68–73 °C, pH 7.0–7.5) from a 10-cm diameter vent at the summit of *Shivlinga*; this is accompanied by a weak emission of hydrothermal gases (a comprehensive characterization of the gaseous discharges of the geothermal vents of Puga valley has shown that they all emit steam, carbon dioxide and hydrogen sulfide^41^). *Shivlinga*’s discharge runs down the side of the microbialite (Fig. 1C,I) onto the bedrock surrounding its base (Fig. 1D,J). Beyond the slopes of the basement rock, the spring-water flows along several shallow channels (the longest one running 3.3 m from *Shivlinga’s* base), and eventually percolates into the regolith of the surrounding apron (Fig. 1B,H). Temperature, pH and flow-rates of the vent-water and outflows were found to remain consistent during the 2008–2013 annual site visits (Table 1 and Supplementary Table 1); this stability was attributable to the physicochemical consistency of the underlying geothermal reservoir^44^. The sizes and structures of the microbial mats growing along *Shivlinga*’s spring-water transit also remained largely unchanged during the period 2008–2013, indicating the microbiome’s resilience to seasonal and annual variations in weather. This observation was consistent with reports for microbial mats at other geothermal sites^22,25^; so *Shivlinga*’s ecosystem was considered suitable for studying microbial community dynamics along its hydrothermal gradients, and a comprehensive geomicrobiological exploration of the site was undertaken in July 2013.

**Table 1:** Microbial communities sampled at the *Shivlinga* ecosystem on 23 July 2013, and the temperature, pH, and flow-rate, of the vent-water, as well as those of the spring-water flowing over the mat samples, at the time of their collection.

Name of the sample	Description of the sample	Distance, in cm, from the vent center*	Temperature, in °C, at the time of sampling	Moisture content, in % w/w, of the *Shivlinga* sinter on which the mat grew^#^	Flow rate of spring-water, in cm s^−1^,at the sampling point^$^	pH
**VW**	Water discharged from *Shivlinga*’s vent	0	70	NA	25	7.0
**VWM**	White streamers made up of filamentous structures	8	66	NA	25	7.1
**DG3**	Green mat containing occasional red patches	11	52	41.7	NA	7.3
**DG4**	Purple mat with a green shimmer	14	41	28.9	NA	7.5
**WG3**	White streamer interspersed with green filaments	22	56	NA	22	7.5
**WG4**	Green mat containing occasional yellow patches	65	46	NA	15	8.0
**WG5**	Rust-colored mat	120	38	NA	11	8.2
**WG6**	Intensely red microbe-mineral nodules	190	36	NA	7	8.3
**WG7**	Dark grey streamers	290	33	NA	5	8.5

*Quantified using a measuring tape running along the flow of water.

^#^Measuring moisture content was applicable only to the samples DG3 and DG4 of the progressively-drying thermal gradient as these mats grew on moist sinter surfaces beyond the outflow channel. Flow rates were measured for all the other communities as they grew directly on the hot water outflow.

^$^Flow rate of water only at the vent (i.e. at sample sites of VW and VWM) was measured as ml s^−1^.

### Chemical characteristics of the vent-water

The chemical composition of *Shivlinga*’s vent-water was determined by collecting surficial discharges from the center of the vent’s orifice. The vent-water, at the time of the current sampling (on 23 July 2013), had a neutral pH (7.0) and a low concentration of total dissolved solids (2000 mg L^−1^) compared with other neutral-pH hot springs located in distinct geographical areas of the world^41,45,46^. *Shivlinga*’s vent-water was found to have high concentrations of boron (175 mg L^−1^), sodium (550 mg L^−1^), bicarbonate (620 mg L^−1^) and chloride (360 mg L^−1^), compared to the other solutes detected in the vent-water. There was also some silicon (60 mg L^−1^), potassium (15 mg L^−1^), calcium (10 mg L^−1^), lithium (6 mg L^−1^) and magnesium (3 mg L^−1^) detected. Of the sulfur species present, thiosulfate (3 mM) and sulfate (1 mM) were the most abundant, but sulfite (225 μM) and sulfide (250 μM) were also present at significant concentrations. Collectively, the vent-water chemistry was consistent with that reported for other hot springs within the Puga region^40,41^.

### Distinct mineralogies of *Shivlinga*’s vent and body, bedrock slope, and apron

**Figure 2:**
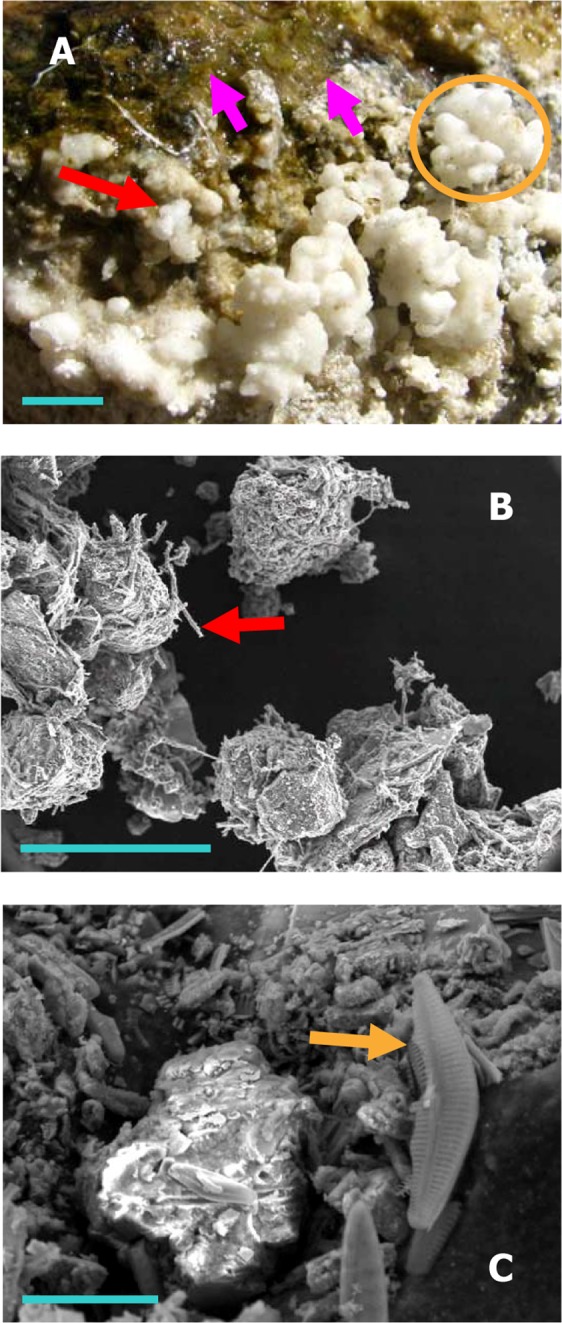
Macro-/micro-scale microbe-mineral structures at the *Shivlinga* site: (**A**) green microbial mat (pink arrows), and white spherules (red arrow) that collectively form shrub-like mineral bodies (orange circle), on the upper surfaces of the vent’s rim (blue scale bar = 10 mm); (**B**) sinter particles from 5-cm-deep inside the wall of the microbialite, associated with bacterial filaments (red arrow) (image was taken using SEM, blue scale bar = 150 µm); and (**C**) a diatom cell (golden arrow) associated with the particles shown in (**B**) (using SEM, blue scale bar = 20 µm).

For each of the water-flows running in *Shivlinga*’s vent-to-apron trajectory, and starting from the surface of the vent-water, multi-colored microbial mats grow all along their transits, until the end of the flow near the edge of the apron. Depending on their thickness, the mats are either slightly submerged or stay just above the water level. Segments of mats that lie at the interface of the spring-water and regolith are typically dry at their surface but moist within. Every mat at the site is intermeshed with fresh mineral accretions that precipitate from the cooling spring-water and also condense from the gases that arise from the vent; but mineralization processes are at far more advanced stages in the less-hydrated segments of mats. For microbial biomass growing just beneath the water level, or protruding from the water surface by some mm to cm, mineral dusts visible to the naked eye are present on the mat surfaces. For those mats or parts of mats that are situated at the margins of outflows, mm- to cm-sized, soft, white spherules and shrub-like bodies of accreted minerals cover the surface (Fig. 2A). In the latter cases, mineralization is very conspicuous: monitoring this process over a period of 21 days using close-up photography revealed that the microbial mats grow out from the top of the encrustations, and spread again over the surface of the spherules (Supplementary Fig. 2). It was also evident that the fresh biomass at the surface of such mats are covered by fresh mineral deposition; and so the process continues. Mineralization acts to solidify all the mat structures of the microbiome from the bottom upwards. The microbialite thereby grows in height as well as in girth, besides hardening from within; the height of *Shivlinga*, according to our field observations, increased by ~4 cm (and the vent orifice narrowed slightly) between 2008 and 2013. Spherules and shrub-like structures form across the apron’s dry surface, presumably due to condensation of *Shivlinga*’s fumarolic gases on the regolith (Fig. 1B,H). However, the salt accretions which form beyond a few cm from the banks of the water-flows are not associated with microbial mats, and are dry and brittle.

We analyzed fine mineral particles accreted on the mat samples taken from the middle of the water-flow, and sinter-spherules precipitating on mat-portions growing near the margins of the water-flow. Sinter-spherules and shrub-like bodies that were soft, so had presumably formed within a few weeks before sampling, were collected from one top-edge of *Shivlinga*’s body (Sinter-Sample 1), one side-surface of the microbialite (Sinter-Sample 2), one point on the bedrock slope around *Shivlinga*’s base (Sinter-Sample 3), and one point at mat:regolith interface on the margin of the spring-water flow across the apron (Sinter-Sample 4) (Fig. 1B–D,H–J). Besides these four samples, older and harder sinter material was collected from *Shivlinga*’s interior by boring the microbialite’s side-surface, using a 3 mm twist drill bit, to a depth of 5 cm at a point located 15 cm below the summit (Sinter-Sample 5) (Fig. 1C,I).

All the sinter samples were found to be rich in boron, sodium and calcium (Supplementary Table 2); kernite (Na_2_B_4_O_7_·4H_2_O) was the major mineral in *Shivlinga*’s interior, while borax (Na_2_B_4_O_7_·10H_2_O) and then tincalconite (Na_2_B_4_O_7_·5H_2_O) were predominant in the soft, recently-formed sinters (data not shown). Calcite (CaCO_3_), gypsum (CaSO_4_·2H_2_O), elemental sulfur, and silica (SiO_2_) were also identified within the boron-mineral matrices of all the five sinter samples. Minute quantities of aluminum, gold, iron, manganese, zinc, molybdenum, lead, silver, nickel and cobalt were also detected, regardless of the sinter samples (Supplementary Table 2). These mineralogical data are consistent with *Shivlinga*’s vent-water chemistry, and together they suggest that the microbialite has formed from the gradual precipitation and accretion of boratic-, carbonatic-, and sulfatic-minerals, a process that, in part at least, is facilitated by its microbial communities (see Fig. 2A).

The process of precipitation of boron minerals is common to *Shivlinga*’s vent and microbialite body, bedrock slope and apron; yet there is considerable variation in the proportions of oxides or carbonates of metals, alkali metals and alkaline earth metals which combine with the boron minerals in the three zones of this microbiome (Supplementary Table 2). The soft (fresh) sinters of the vent and microbialite body, and those of the sloping bedrock, contain greater proportions of aluminum and gold, while sinters of the apron contain greater proportions of iron and manganese. On a w/w basis, the percentages of zinc, molybdenum, lead, silver, nickel and cobalt etc are higher in the freshly-precipitated sinters located within the apron than in the accretions on the vent and microbialite body, or the bedrock slopes (Supplementary Table 2).

### Microbial characterizations along *Shivlinga*’s spring-water transit/dissipation: a wet, and a progressively-drying, thermal gradient

Microbiological investigations were carried out along two distinct axes of *Shivlinga*’s spring-water transit/dissipation. One of these represented a progressively-drying thermal gradient having no running water on it (along this axis, the spring-water dissipated into the mineral sinters of the microbialite body); the other featured a wet thermal gradient along the outflow channel of the spring. Both of these gradients start from the vent and, besides the vent-water community (VW), share a common microbial mat community (VWM) that floats on the vent-water and anchors to the sintered rim of the vent (Fig. 1C,I). The drying thermal gradient traverses the moist sinters concentrically from the vent opening, ~15 cm towards the edge of *Shivlinga*’s flat-topped summit; two more, morphologically distinct but physically contiguous mat communities (DG3 and DG4) occur along this trajectory (Fig. 1C,I). On the other hand, the ~4-m-long wet thermal gradient, at the time of sampling, laid along the longest and widest of the six prominent channels of *Shivlinga*’s spring-water transit; it ran from the vent, down one side of *Shivlinga*’s mineral body and base (Fig. 1C,D,I,J), and then along the outflow channel in the apron (Fig. 1B,H). Along the wet thermal gradient lies a continuum of multi-hued microbial mat communities, from within which five representative mats (WG3, WG4, WG5, WG6 and WG7) were chosen for investigation. In total, nine physically/morphologically distinctive microbial communities, including one in the vent-water (Table 1), were sampled across the two thermal gradients and subjected to analysis using microscopy and metagenomics. These microbiological data were collated with the mineralogical analyses of sinter materials (sediment-accretions) sampled and investigated from the five distinct depositional facies (physical and chemical conditions under which deposition takes place) existing in the *Shivlinga* territory. This revealed that the *Shivlinga* microbiome encompasses three distinct geomicrobiological zones (Fig. 1A,G) characterized by distinctive temperature- and pH-conditions, and topographical, mineralogical and microbiological features. These are (i) the vent and the microbialite body, (ii) the microbialite’s base (including the sloping bedrock) and (iii) the apron. Whilst the first geomicrobiological zone encompassed the VW, VWM, DG3, DG4 and WG3 communities (Fig. 1C,I), the second and the third included the communities WG4 and WG5 (Fig. 1D,J), and WG6 and WG7 (Fig. 1B,E,F,H,K,L), respectively. Further details of the geomicrobial features present along *Shivlinga*’s spring-water transit/dissipation are given in the Methods sub-section titled “Site Description”.

### Distinct mat morphologies of the vent surface, microbialite body, sloping bedrock-base, and apron

**Figure 3:**
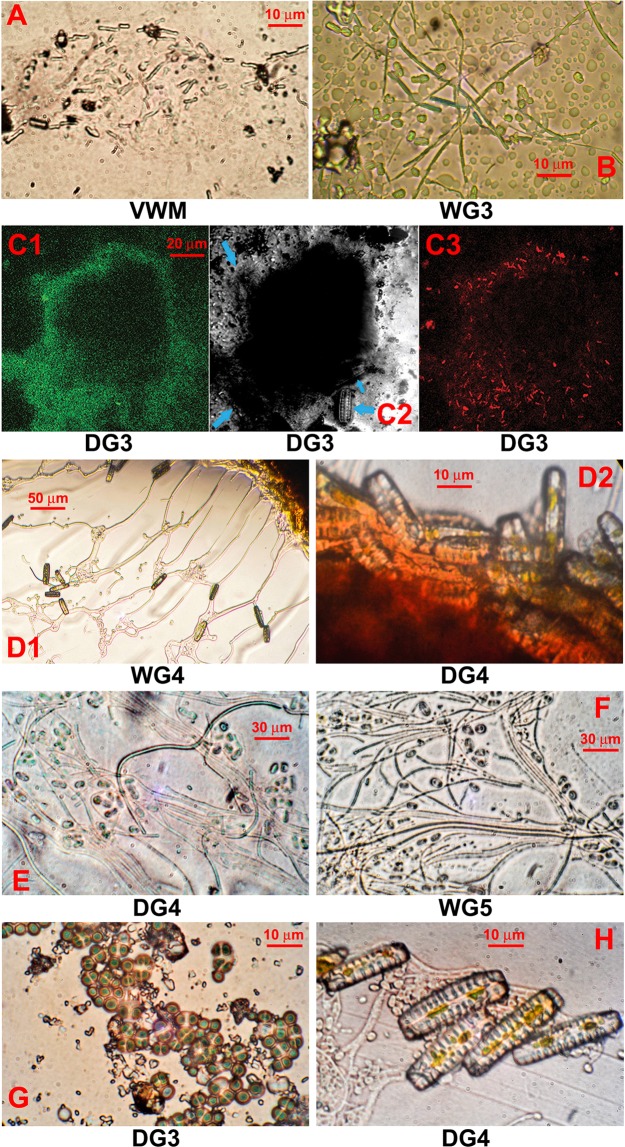
Microbial structures from the *Shivlinga* site: (**A** and **B**) microbial cell filaments from streamers of VWM and WG3, respectively; (**C**) microbial biomass of DG3; (**D**) diatoms from the surface of the microbial mass which makes up WG4 and DG4 (**D1** and **D2** respectively); (**E** and **F**) cells from the core of the microbial mass which makes up DG4 and WG5, respectively; (**G**) Chroococcales-like cyanobacteria in the core of the microbial mass which makes up DG3; (**H**) fungal hyphae and diatoms near the edge of the microbial mass which makes up DG4. All images were produced using phase contrast microscopy except for (**C**) that used laser scanning confocal microscopy. For (**C1**), excitation was carried out at 488 nm and detection at 630–650 nm; (**C2**) is a differential interference contrast image with blue arrows indicating diatom cells; and for (**C3**), excitation was carried out at 543 nm and detection at 650 nm.

Microscopic examinations revealed four distinct patterns of organization of microbial cells in *Shivlinga*’s mat communities. The VWM and WG3 streamers are composed of rod-shaped bacterial cells that lie end-to-end, interspersed with smaller coccoidal cells, within dense networks of aseptate filaments of cyanobacteria that are long, straight, sheathed and semi-rigid (Fig. 3A,B). All the green, red and purple mats growing around the vent, on the microbialite body, and the bedrock slope of *Shivlinga* (i.e. DG3, DG4, WG4 and WG5) are made up of spherical bodies ranging from 10–100 µm in diameter (Fig. 3C). Diatoms that appear to be members of Cymbellaceae form the margins of these spheres, and remain in immediate contact with adjacent spheres or other mineral structures (Fig. 3C,D). Cells, which have the dimensions and morphologies of prokaryotic cells, occur along the boundary of the spheres. Filamentous and coccoidal microorganisms, resembling *Chloroflexi* and Chroococcales respectively, occupy the internal core of the spheres; abundant hyphal structures that are aseptate and white (some have stalked buds coming out of them), and resemble Phycomycetian fungi are also seen here (Fig. 3E–H). Notably, boron mineral-encrusted forms of these spherical bodies were identified in SEM-EDS of Sinter-Sample 5 (Fig. 2B,C). These microscopic data, together with the detection of small but definite proportions of fungus-affiliated reads in the subsequent metagenome analysis of the mat communities, indicated that specific amplification and sequencing of fungal DNA, in future investigations, may reveal even greater fungal diversity than shotgun metagenomics indicates.

**Figure 4:**
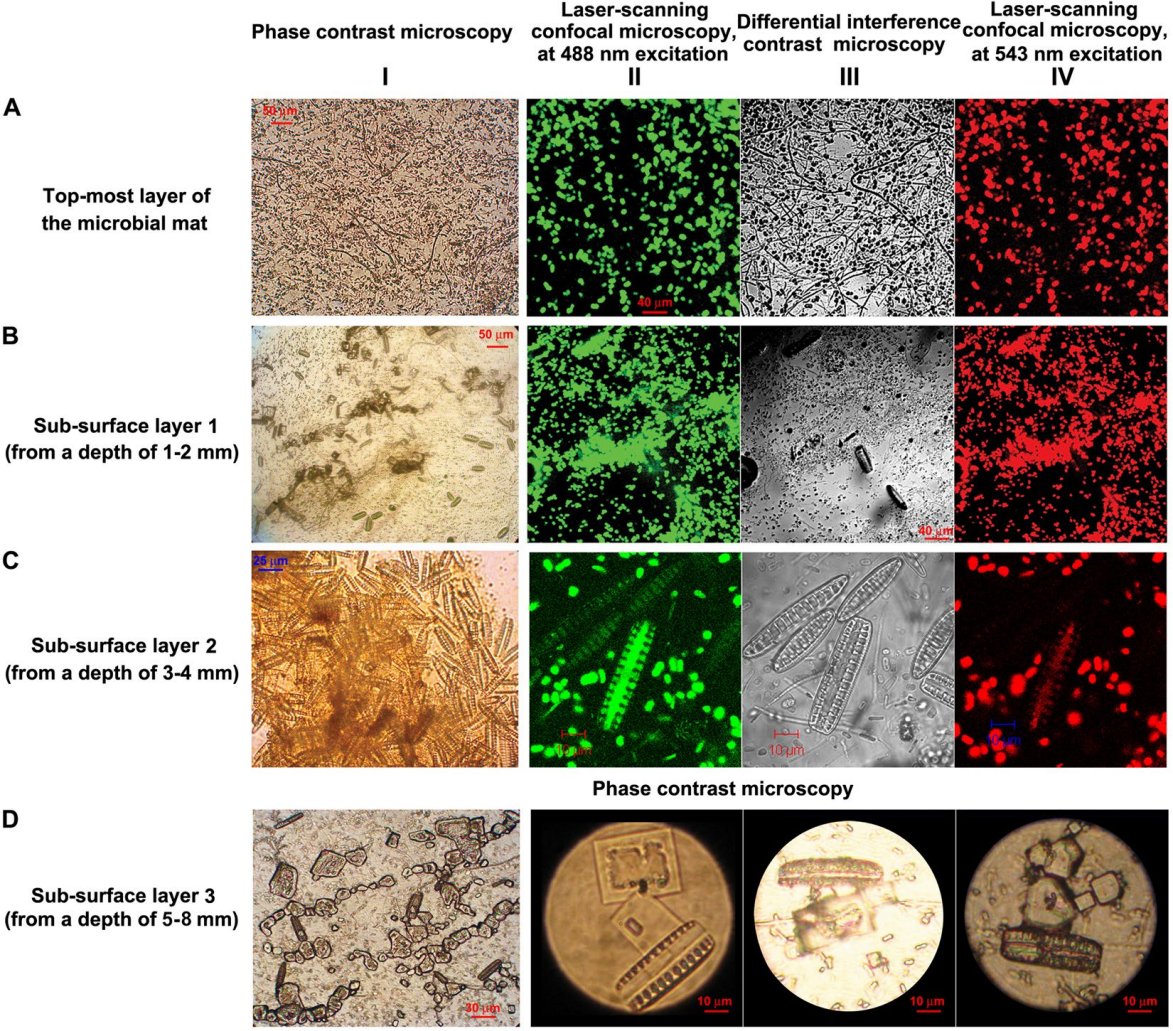
Microbial cells present in the different layers of the WG7 sample: (row **A**) top-most layer of the microbial mat; (row **B**) sub-surface layer 1 (from a depth of 1–2 mm); (row **C**) sub-surface layer 2 (from a depth of 3–4 mm); and (row **D**) diatom cells and borax crystals in sub-surface layer 3 (from a depth of 5–8 mm); (Column **I**) images were taken using phase contrast microscopy; (column **II**, except for row **D**) images were taken using laser scanning confocal microscopy, at 488 nm excitation and 630–650 nm detection; (column **III**, except for row **D**) used differential interference contrast to modify the image from column II; and (column **IV**, except for row **D**) used laser scanning confocal microscopy (543 nm excitation and long pass 650 nm detection) to further modify the image from column II. Photographs in row **D** were taken using phase contrast microscopy.

The two morphologically distinct mat structures of the wet thermal gradient that are located within apron environment, i.e. WG6 and WG7, suggest processes by which mat formation had occurred. The former type appears to have come about via nucleation of borax, extracellular organic matter, and traces of calcite and other minerals, on irregular aggregations of diatoms, cyanobacteria, red-pigmented *Chloroflexi*, and other cells having dimensions and morphologies akin to those of prokaryotes. The WG7 streamers, in contrast, exhibited a laminar organization. The top-most layer of these stratified mats contained dense sheaths of filamentous bacteria resembling *Chloroflexi*, *Leptothrix* and *Sphaerotilus*. Embedded in complex mineral assemblages, these filamentous organisms are interspersed with large round-shaped cells that appear to be flagellates, according to their morphologies and 5–20 µm diameters (row A of Fig. 4). At 1–2 mm beneath the top-layer (i.e. in sub-surface layer 1), abundance of the filamentous bacteria decreases and the density of the eukaryotic cells increases; at these depths, there are also occasional cells of pennate diatom species (row B of Fig. 4). Diatoms are, however, much more abundant at subsequent depths up to the boratic substratum. At 3–4 mm from the mat surface (i.e. in sub-surface layer 2), the diatoms co-exist with morphologically-diverse bacteria, many of which resemble purple sulfur and non-sulfur bacteria (row C of Fig. 4). At the deepest layer of WG7, 5–8 mm below the surface (i.e. in sub-surface layer 3), diatom cells predominate and are attached to borax crystals (row D of Fig. 4).

### Bacteria-dominated vent-water community

PCR amplification (using Bacteria-/Archaea-specific oligonucleotide primers), followed by high throughput sequencing, of the V3 regions of all 16S rRNA genes present in the total environmental DNA extracted from the VW sample revealed the alpha diversity of the community. This is referred to hereafter as the metataxonomic composition^47^ so as to distinguish the diversity reported for the VW community from the diversities reported subsequently for the mat communities on the basis of shotgun metagenome sequencing and analysis. Notably, total environmental DNA yield from the VW sample was insufficient for direct shotgun sequencing and metagenome analysis.

Bacteria-specific V3 primers generated PCR products of desired size (~200 bp), so the amplification product was sequenced at high data throughput. Archaea-specific V3 primers did not yield any PCR product, suggesting that very low numbers of archaeal cells are present in *Shivlinga*’s vent-water. Reads of the Bacteria-specific V3 sequence dataset were clustered into operational taxonomic units (OTUs) or putative species-level entities unified at the level of 97% 16S rRNA gene sequence similarity (Supplementary Table 3 shows the summary results of OTU clustering). Rarefaction analysis of the dataset confirmed that the read-sampling level (data throughput) achieved was sufficient to reveal most of the diversity present in the sample (Supplementary Fig. 3).

Total 64 bacterial OTUs - distributed over the phyla *Proteobacteria* (45), *Actinobacteria* (4), *Firmicutes* (4), *Deferribacteres* (2), *Deinococcus*-*Thermus* (2), *Ignavibacteriae* (2), *Armatimonadetes* (1), *Aquificae* (1), *Synergistetes* (1) – were identified in the VW sample (number of OTUs affiliated to each phylum is given in parenthesis); 2 of these belonged to unclassified Bacteria. Of the 64 OTUs, 19 could be classified at the genus level. The genera identified (number of affiliated OTUs given in parenthesis) were *Achromobacter* (2), *Alcaligenes* (1), *Aminicenantes* Incertae Sedis (1), *Armatimonadetes* gp5 (1), *Calditerrivibrio* (2), *Ignavibacterium* (2), *Paenibacillus* (1), *Propionibacterium* (1), *Sulfurihydrogenibium* (1), *Thermomonas* (4), *Thermus* (2) and *Thiofaba* (1). Notably, out of the 12 genera detected metataxonomically in the 70 °C VW, member strains of at least four have never been found to grow at >45 °C *in vitro* (these are *Achromobacter*, *Alcaligenes*, *Paenibacillus* and *Propionibacterium*); and six are present in each of the mat communities studied (these are *Achromobacter*, *Calditerrivibrio*, *Paenibacillus*, *Propionibacterium*, *Sulfurihydrogenibium* and *Thermus*).

### Microbiology of *Shivlinga*’s thermal gradients

Variations in the community composition of *Shivlinga*’s microbial mats were assessed via metagenome sequencing and analysis along each thermal gradient (Supplementary Tables 4 and 5 show the summary statistics plus domain-level classifications of the metagenomic data obtained from the mats of the drying and wet thermal gradients respectively). Although total number of metagenomic reads generated for the different samples varied, plateauing of rarefaction curves determined for the individual samples by plotting number of reads analyzed versus genera identified in searches against the non-redundant (nr) protein sequence database of National Center for Biotechnology Information (NCBI, USA) or the 16S rRNA gene sequence database of the Ribosomal Database Project (RDP) showed that the data throughput achieved was sufficient to reveal most of the diversity present in the sample. Furthermore, to avoid the risk of potential anomalies that may arise in the subsequent analyses due to differential read-count of the different samples, prevalence of taxa was quantified as the percentage of total metagenomic reads ascribed to them, i.e. relative abundance.

In searches against the nr protein database, unassigned and unclassified sequences made up 7–26% of the metagenomic readsets; Bacteria accounted for at least 72% of all these and Archaea constituted <1%, regardless of the sample. The highest relative abundance of Archaea was encountered in the distal (cooler) ends of the gradients, i.e. in DG4 (41 °C), WG4 (46 °C) and WG7 (33 °C) communities. The genera *Archaeoglobus*, *Methanocaldococcus*, *Methanococcus*, *Methanosarcina*, *Methanospirillum*, *Methanothermobacter*, *Pyrococcus* and/or *Thermococcus* comprised the major archaeal component of every mat community. Eukarya accounted for <0.6% of the metagenomic readsets for VWM, WG3 and WG4; and ~1% for DG3, DG4; WG5, WG6, and WG7 (for at least one replicate). In other words, relative abundance of eukaryotes almost doubled along the drying and wet thermal gradients at ≤52 °C and ≤38 °C (respectively), compared to those observed at higher temperatures. Diverse types of virus are present *Shivlinga*’s mat communities and, remarkably their relative abundance is highest at 66 °C (in VWM). Virus-affiliated reads made up 0.3% and 0.2% of the metagenomes obtained from the two VWM sample-replicates, but only 0.01–0.04% of those obtained from the other mat communities. Predominant members of the viral component of VWM metagenomes were temperate lactococcal phages, including r1t and *Listeria* phi-A118; notably, thermophilic phages that are commonly found in other hydrothermal ecosystems^48^ were absent. Whereas the Bacteria are the dominant organisms in *Shivlinga*’s microbiome, the ecophysiological importance of eukaryotic microbes could be greater than suggested by their low number of metagenomic reads. Some indications of this are detailed below.

Microscopic analyses revealed dense but localized populations of diatoms in many of the mat communities, including those located at 52 °C (Fig. 3C,D). Diatoms (Bacillariophyta) were also detected metagenomically in all of the microbial mats sampled, albeit in low numbers. Their relative abundance ranged from a minimum of 0.001%, for VWM, to a maximum of 0.05%, for DG3, along the drying thermal gradient and 0.6%, for WG5, along the wet thermal gradient (relative abundances values for diatoms were 0.2% for DG4, WG6 and WG7). The genera *Odontella*, *Phaeodactylum* and *Thalassiosira* consistently predominate diatom populations in all the eight mat communities, regardless of the hydrothermal gradient.

Microscopy revealed localized but dense populations of fungi in mat communities present at the 33–52 °C sites. According to metagenomic analyses, fungi were also present in all of the other mat communities, albeit at low levels. Along the drying thermal gradient, their relative abundance ranged from a minimum of 0.02%, for VWM, to a maximum of 0.2%, for DG4, and along the wet thermal gradient from 0.02% (VWM) to 0.7%, for WG5. Furthermore, 63 fungal genera were found to be present in VWM (66 °C), while greater numbers of genera were detected in the communities growing at the distal ends of each gradient; e.g. 82 genera in DG4, 106 in WG5, and 142 in WG7. *Aspergillus*, *Gibberella*, *Neurospora*, *Saccharomyces*, *Schizosaccharomyces* and *Ustilago* constituted the major portions of the fungal populations across both the gradients.

Sequences matching Chlorophyta (green algae) were present in the metagenomes of all the mat communities, and their relative abundance ranged from 0.005% (for VWM) to a maximum of 0.1% (for DG4, WG5, WG6 and WG7). The genera *Chlamydomonas*, *Chlorella*, *Micromonas*, *Ostreococcus* and *Volvox* are the major green algal component of all the mat communties, whereas *Acetabularia*, *Bryopsis*, *Dunaliella*, *Nephroselmis*, *Pyramimonas* and *Scenedesmus*, sparse at high temperatures, increase towards the distal ends of the gradients. Remarkably, the eukaryotic components of each mat metagenome were not dominated by a microorganism, but by the bryophyte *Physcomitrella* (spreading earthmoss) which has a simple life-cycle, and is considered to be the most-ancient of all land plants^49^. It is an early colonizer of exposed mud/ sediment/ soil around the edges of water bodies, and is widely distributed in temperate regions of the world. At the *Shivlinga* site, *Physcomitrella* is most abundant in VWM (66 °C), where it constitutes 0.1% of the metagenomes. Notably, in the VWM metagenomes, known thermophilic genera such as *Archaeoglobus*, *Calditerrivibrio*, *Desulfotomaculum*, *Hydrogenivirga*, *Pyrococcus*, *Thermoanaerobacter* and *Thermocrinis*, each has <0.1% of reads ascribed to them. In the other mat communities, *Physcomitrella* constituted 0.02–0.04% of the metagenomes. In VWM, the most prevalent eukaryote after *Physcomitrella* is another photosynthetic organism, *Cyanidioschyzon* which is a unicellular, ~2 μm-long red alga that often occurs in sulfur-containing, highly acidic hot springs at around 45 °C^50^. For all other mat communities, the second-most prevalent eukaryote is the amoeba *Dictyostelium* which is found in soils, predates bacteria, and is commonly known as slime mold^51^. The other protists detected in *Shivlinga*’s mat communities included the flagellate cryptomonad alga *Guillardia*, the glaucophyte *Cyanophora*, and the chloroplast-bearing amoeba *Paulinella*. Notably, all these genera were present in sample sites of ≤46 °C.

**Figure 5:**
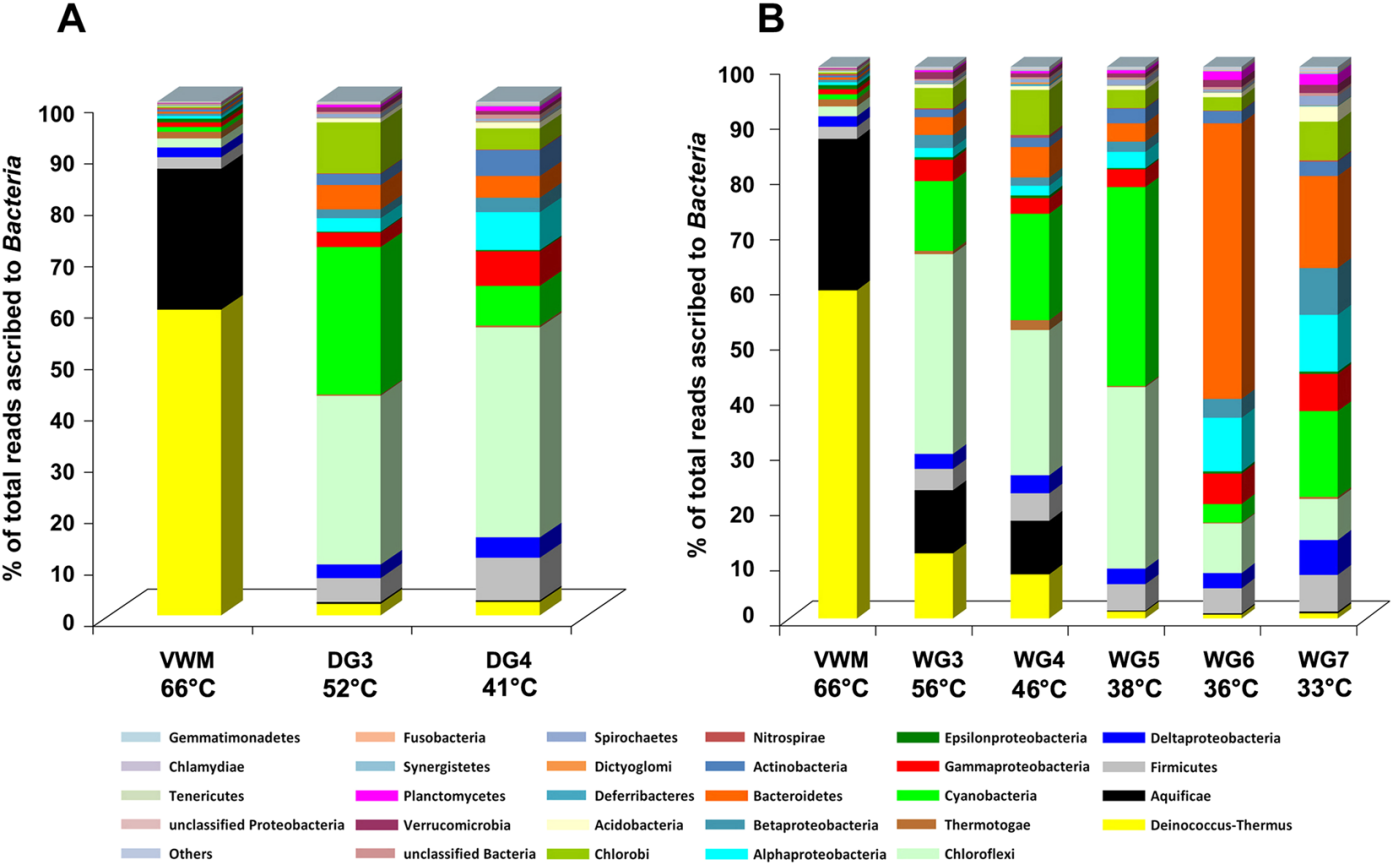
Mean percentage of reads ascribed to bacterial phyla, and classes within the *Proteobacteria*, in the duplicate metagenomes obtained from each mat community of (**A**) the drying thermal gradient and (**B**) the wet thermal gradient. The 21 phyla and five proteobacterial classes represented account for >0.1% reads in at least one of the 16 metagenomes analyzed. The category ‘others’ encompasses the phyla that accounted for <0.1% reads in every metagenome analyzed. Statistical significance of the fluctuations in the relative abundance of the taxa along the hydrothermal gradients can be seen in Supplementary Figs. 4 and 5 where their mean relative abundance within each mat community has been plotted alongside the two original relative abundance values obtained from the duplicate metagenomes (shown as vertical range bar in Supplementary Figs. 4 and 5).

Along the drying, as well as the wet, thermal gradients (Fig. 5A; Supplementary Fig. 4 and Fig. 5B; Supplementary Fig. 5), mean relative abundances of the 26 major groups of Bacteria (i.e. 21 phyla and five proteobacterial classes) varied considerably. *Deinococcus*-*Thermus* and *Aquificae* dominate VWM, and are less prevalent in the communities located at the distal (cooler) ends of each gradient. Along the drying thermal gradient, this decline in the prevalence is very sharp for both phyla; relative abundance of *Deinococcus*-*Thermus* declined from 60% in VWM to ~2% in DG3 and DG4. In the drying thermal gradient, there was a concurrent increase of *Chloroflexi*; *Firmicutes*; *Alpha*, *Beta*, *Gamma* and *Delta* classes of *Proteobacteria*; *Actinobacteria*; *Acidobacteria* and *Planctomycetes*, which continues through DG3 and DG4. Notably, *Cyanobacteria*, *Chlorobi*, *Bacteroidetes*, *Spirochaetes* and *Verrucomicrobia* are more abundant in DG3 (relative to VWM), but declined in DG4 (relative to WG3). Contrary to the above trends, *Epsilonproteobacteria* and *Thermotogae* were low in DG3 (relative to their VWM levels) but increased again in DG4.

Phylum-level population fluctuations are more irregular along the wet thermal gradient; the relative abundances of *Alphaproteobacteria*, *Chlamydiae* and *Planctomycetes* continue to increase along the water transit, in the vent-to-apron direction. The frequencies of *Actinobacteria*, *Cyanobacteria* and *Spirochaetes* also increase up to the 38 °C site. Similarly, *Acidobacteria*, *Bacteroidetes*, *Chlorobi*, *Deferribacteres*, *Deltaproteobacteria*, *Dictyoglomi*, *Firmicutes*, *Fusobacteria*, *Gemmatimonadetes*, *Nitrospirae*, *Synergistetes* and *Tenericutes* increase up to the 46 °C site. By contrast, the frequencies of *Aquificae* and *Epsilonproteobacteria* continue to decrease along the gradient, down to the 38 °C sample site, and *Deinococcus*-*Thermus* decreases down to the 36 °C site. Frequencies of *Betaproteobacteria*, *Chloroflexi*, *Gammaproteobacteria* and *Verrucomicrobia* increase till the 56 °C site. Remarkably, the relative abundance of *Thermotogae* does not vary with temperature along the wet thermal gradient. Consistent with these trends, only *Aquificae*, *Deinococcus-Thermus* and *Epsilonproteobacteria* exhibited significant positive correlation with both temperature and flow-rate of the spring-water (Supplementary Table 6). Whereas correlation of these three phyla with pH was significantly negative, only *Aquificae* showed significantly negative correlation with distance from the vent. *Actinobacteria*, *Alphaproteobacteria*, *Chlamydiae*, *Firmicutes*, *Fusobacteria*, *Gammaproteobacteria*, *Gemmatimonadetes*, Other phyla, *Planctomycetes*, *Spirochaetes*, Unclassified Bacteria and Unclassified *Proteobacteria*, all exhibited significant negative correlations with both temperature and flow rate. All these groups, remarkably, had significant positive correlations with pH and distance from the vent. *Acidobacteria*, *Bacteroidetes*, *Betaproteobacteria*, *Chlorobi*, *Chloroflexi*, *Cyanobacteria*, *Deferribacteres*, *Deltaproteobacteria*, *Dictyoglomi*, *Nitrospirae*, *Synergistetes*, *Tenericutes*, *Thermotogae* and *Verrucomicrobia* showed no significant correlation with temperature, although *Acidobacteria*, *Betaproteobacteria*, *Deltaproteobacteria*, *Synergistetes* and *Verrucomicrobia* had positive correlations with pH as well as distance from the vent – of these, *Acidobacteria* and *Betaproteobacteria* additionally exhibited significant negative correlations with flow rate. *Tenericutes* had significant positive and negative correlations with pH and flow rate respectively. Supplementary Tables 7–12 show the calculations for all the correlation coefficients and their Benjamini-Hochberg-corrected *P* values.

### Thermodynamic constraints on microbial colonization

Thermodynamic constraints on the microbial colonization of *Shivlinga* sites were identified based on relative abundance of taxa along the hydrothermal gradients. These, in turn, were derived from the percentage of metagenomic reads ascribed to individual taxa within each community. Diversity within *Shivlinga*’s microbial communities increases towards the distal ends of the thermal gradients, and community composition changes from *Aquificae*/*Deinococcus*-*Thermus*- to *Chlorobi*/*Chloroflexi*/Cyanobacteria-dominated, and eventually to a *Bacteroidetes*/*Proteobacteria*/*Firmicutes*-dominated one (Fig. 5). Although the VWM community on *Shivlinga*’s vent-water is biomass-dense, its microbial diversity is lower than that of the mat communities located at the distal end of each thermal gradient. This is likely due to the habitability barrier imposed by the high vent-water-temperature that, in turn, is exacerbated by the low atmospheric pressure. At an altitude of 4438 m, atmospheric pressure is ~6.4 KPa, which is close to the 2.5–5 KPa threshold that is known to prevent *in vitro* growth of bacteria adapted to atmospheric pressure at sea level (i.e. ~101.3 kPa)^52^. Low pressure not only reduces the boiling point of water but can also contribute to the entropic destabilization of biomacromolecular systems (conversely, high pressures appear to mitigate against the chaotropicity of MgCl_2_ in deep-sea brine systems^5^). Conditions at the *Shivlinga* site, therefore, destabilize biomacromolecules, and elicit cellular stress responses which impose high energetic cost on microbial systems and/or can ultimately cause cell-system failure^53,54^. For VW and VWM (the communities at the highest temperatures) an environment-driven selection for thermotolerant/thermophilic taxa would be expected. Accordingly, relative abundance of 19 out of the 26 major bacterial phyla/proteobacterial classes present along the wet thermal gradient was lower in VWM (66 °C) than in WG3 (56 °C). Only *Aquificae*, *Deferribacteres*, *Deinococcus*-*Thermus*, *Dictyoglomi*, *Epsilonproteobacteria*, *Nitrospirae* and *Thermotogae*, which are made up mostly of thermophiles, were more prevalent in VWM than in WG3 (Fig. 5B). This is indicative of a substantial thermodynamic barrier to microbial colonization at ~60 °C. The 19 groups, which include *Alphaproteobacteria*, *Betaproteobacteria and Gammaproteobacteria*, *Bacteroidetes*, *Chlorobi*, *Chloroflexi*, *Cyanobacteria*, and *Verrucomicrobia*, and for which prevalence was lower at 66 °C than at 56 °C, are made up mostly of mesophilic or thermotolerant members incapable of laboratory growth at >60 °C. The presence of these taxa at *in situ* temperatures of >60 °C, suggests that (a) hitherto unidentified environmental factor(s) is (are) acting to mitigate the cellular stresses induced by high temperature. Furthermore, the relative abundances of most of the major phyla/classes (20 out of 26) were lower at 56 °C, in WG3, than at 46 °C, in WG4 (Fig. 5B; Supplementary Fig. 5). This suggests that temperatures between 56 °C and 46 °C impose an additional thermodynamic barrier to habitability for many of the microbial taxa. In microbiomes for which habitability of some taxa is constrained by other parameters, a comparable drop in diversity is observed for xerophiles and halophiles at 0.720 water activity^55^. Furthermore, at the distal end of the wet thermal gradient, the relative abundances of 16 out of the 26 major groups were lesser at 46 °C (WG4) than at 38 °C (WG5), implying that there is another colonization barrier at 40–42 °C.

**Figure 6:**
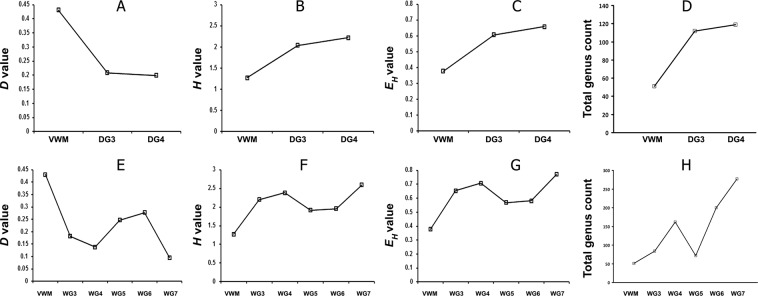
Simpson Dominance (**A**,**E**), Shannon Diversity (**B**,**F**) and Shannon Equitability (**C**,**G**) Indices, and total genus count (**D**,**H**), along the drying (**A**–**D**), and the wet (**E**–**H**), thermal gradients.

Simpson Dominance (*D*), Shannon–Wiener Diversity (*H*), and Shannon–Wiener Evenness (*E*_*H*_) Indices were calculated based on the metagenomic data for each mat community (Supplementary Tables 13–20); these values were then compared along each gradient. The trends revealed provide further evidence for temperature barriers to habitability. For VWM (66 °C), the *D* value was greater than for any other mat community, whereas *H* and *E*_*H*_ were low relative to those for WG3 and DG3 (56 and 52 °C respectively) (Fig. 6). This indicates that, for both the wet, and the drying, thermal gradients, there is a colonization-barrier for many taxa at ~60 °C. However, along the wet thermal gradient, from 56 to 46 °C (WG3 to WG4), and along the drying thermal gradient, from 52 °C to 41 °C (DG3 to DG4), increases in *H* and *E*_*H*_, were much lower than those recorded in the respective gradients, across the 60 °C barrier (Fig. 6). This indicated that colonization-barriers at lower temperatures (~50 °C for the wet thermal gradient and 46–50 °C for the drying thermal gradient) are weaker than that at ~60 °C. Trends of the above indices further revealed that below 46 °C, along the wet thermal gradient, diversity did not correlate with temperature, so other biotic and/or abiotic parameters could be acting as determinants of community composition in this territory.

For each mat community, the most-abundant genera that collectively accounted for ≥50% of all classifiable reads were identified from the results of the metagenome-searches against nr protein database. The number and identities of such genera were then compared along the thermal gradients to interpret the temperature-constrained community dynamics. One genus, *Thermus*, accounted for a mean 50.6% of VWM metagenomes, and the genera *Sulfurihydrogenibium*, *Meiothermus* and *Aquifex* constituted 21.6%, 2.2% and 2.1%, respectively. For the DG3 and DG4 communities, 50% of their metagenomic reads were made up of 12 and 50 genera, respectively (Supplementary Table 21), and for WG3, WG4, WG5, WG6 and WG7, 50% of reads represented one, 11, 17, 35, 68 and 81 genera, respectively (Supplementary Table 22). In order to confirm the identifications of genera present in the individual mat communities, genus-level analysis of their duplicate metagenomes was carried out by searching against the 16 S rRNA gene sequence database of RDP. Using this approach (which is distinct from the amplified 16 S rRNA gene sequence-based metataxonomic approach, used only for the vent-water community), diverse bacterial, but no archaeal, genera were identified in all the eight mat communities (Supplementary Tables 23–30). The numbers of genera detected in this way varied along the two thermal gradients, and were consistent with the variations in microbial diversity indices (Fig. 6). For the drying thermal gradient, genus count was considerably less at 66 °C than at 52 °C or 41 °C (Fig. 6D). For the wet thermal gradient, genus count was far less at 66 °C than at 56 °C or 46 °C; unexpectedly, however, genus count was higher at 46 °C than at 38 °C, even though it was much lower at 38 °C than at 36 °C or 33 °C (Fig. 6H). The trends in microbial diversity that were revealed from the direct classifications of the metagenomic reads were consistent with the results of diversity analyses based on assembly of contigs and binning of population genomes from the mat metagenomes (Supplementary Note 2).

### Key metabolic attributes of the *Shivlinga* mat communities

**Figure 7:**
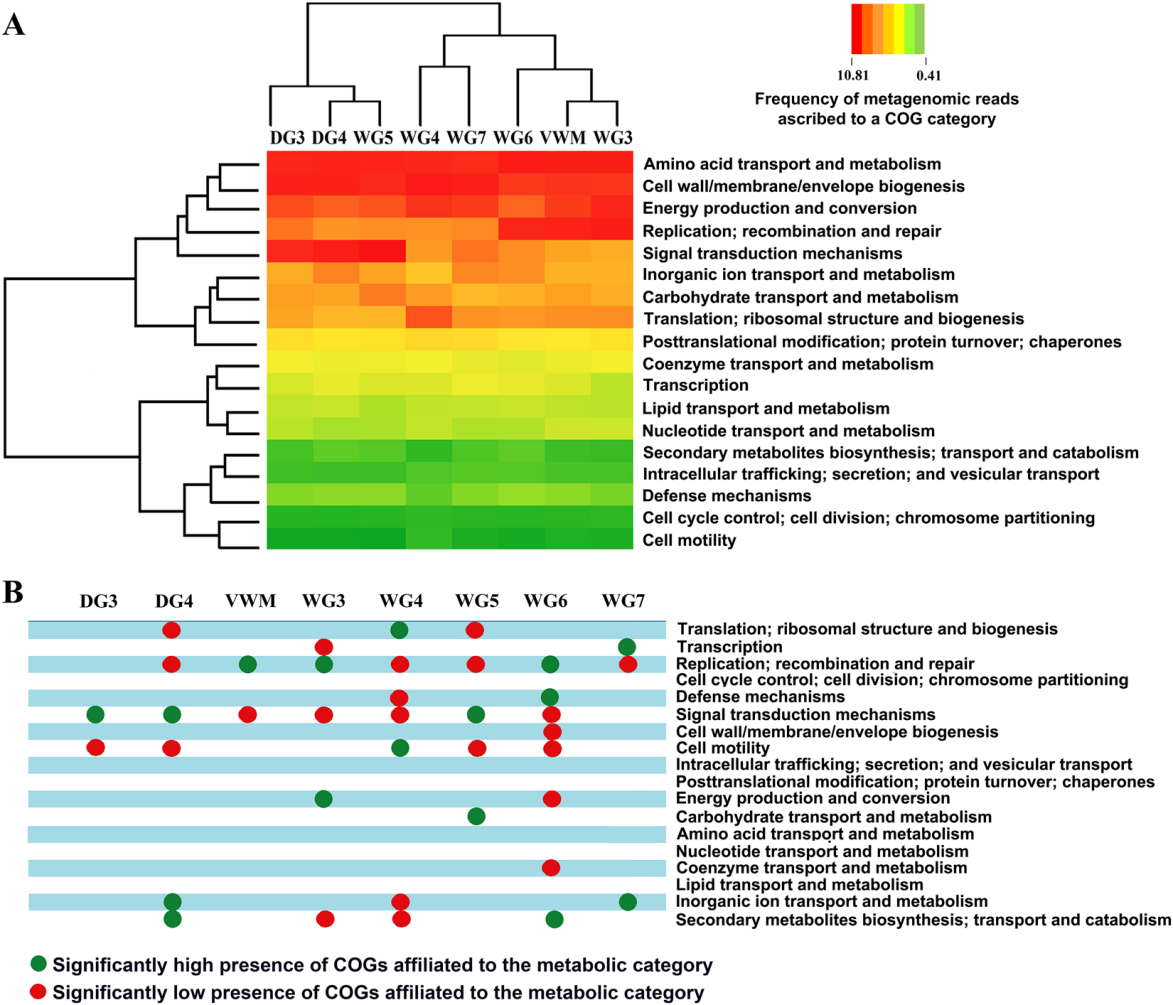
Functional analysis of the metagenomes isolated from the eight microbial mat communities of *Shivlinga*: (**A**) heat map comparing the richness of the metabolic/functional categories across the communities, determined in terms of the number of Clusters of Orthologous Groups (COGs) of Proteins that are ascribed to the categories in individual communities; a two-dimensional clustering is also shown, involving the eight mat communities on one hand and the 18 functional categories of COGs on the other; color gradient of the heat map varied from high (red) to low (green), through moderate (yellow), richness of the categories across the communities; (**B**) Statistically significant high (green circles) or low (red circles) richness of the functional categories across the communities, as determined by Chi Square test with p < 0.001.

Analysis of the metagenomic data of individual mat communities for comparative richness of genes [or Clusters of Orthologous Groups (COGs) of Proteins] under various metabolic/ functional categories was followed by hierarchical clustering of the mat communities in terms of their enrichment of various COG categories. This revealed a dichotomy between DG3, DG4 and WG5 on one side, and VWM, WG3, WG4, WG6 and WG7 on the other (Fig. 7A). DG4 and WG5 clustered on the basis of their similarities with respect to high/low presence of COGs affiliated to the categories Cell motility; Replication, recombination and repair; Signal transduction mechanisms; and Translation, ribosomal structure and biogenesis. DG3 joined this cluster based on significantly high and low presence of COGs affiliated to Signal transduction mechanisms and Cell motility, respectively (Fig. 7B). In the other major cluster, the closeness of VWM and WG3 (Fig. 7A) is explained by their similarities in having high and low presence of COGs affiliated to the categories Replication, recombination and repair, and Signal transduction mechanisms, respectively (Fig. 7B). WG6 joined the VWM-WG3 cluster on the basis of significantly high presence of COGs affiliated to Replication, recombination and repair (Fig. 7B). WG4 and WG7, in turn, associated on the basis low presence of COGs for Replication, recombination and repair. VWM, WG3, WG4 and WG6 were further unified by the significantly low presence of COGs affiliated to Signal transduction mechanisms. Despite the significantly low presence of COGs affiliated to Signal transduction mechanisms in a number of mat communities, number species-level matches as well as relative abundance for genes encoding bacterial two component kinases such as histidine kinase, serine threonine protein kinase, diguanylate cyclase and PAS sensor protein were considerably high for all the communities. This implied that the geothermal adaptations of complex microbial mat communities involve efficient response regulations to a wide range of environmental signals.

The merged metagenomic readsets of individual mat communities were searched for genes putatively encoding reverse gyrase enzymes, which are typical of hyperthermophilic bacteria and archaea, but never found in mesophilic microorganisms^56^. As expected, metagenomic reads matching reverse gyrases from 30 different bacterial species were identified in VWM, but in no other community of the drying thermal gradient. Along the wet thermal gradient, such reads were found exclusively in communities living at ≥46 °C.

Metagenomic reads matching genes that encode key enzymes of the autotrophic Calvin–Benson–Bassham and Wood–Ljungdahl pathways, namely ribulose 1,5-bisphosphate carboxylase large chain (*rbcL*) and acetyl-coenzyme A synthetase (*acsA*), were identified in all the mat communities. Even as autotrophy is ubiquitous throughout the ecosystem, Wood–Ljungdahl pathway is apparently utilized by majority of the community members across *Shivlinga*’s thermal gradients. The numbers of species-level matches identified for *acsA*-related reads were an order of magnitude greater than those for *rbcL*; across the communities, species-level matches for *acsA* ranged between 112 (in WG5) and 477 (in WG7) while those for *rbcL* ranged from 18 (in DG4 and WG5) to 54 (in WG7). Furthermore, metagenomic reads corresponding to genes encoding the large subunit of ATP citrate lyase (AclA), a key enzyme of the reverse tricarboxylic acid (rTCA) or Arnon–Buchanan cycle - which is more energy-efficient, oxygen-sensitive, and phylogenetically ancient than the Calvin cycle or the Wood–Ljungdahl pathway^57,58^ - were detected only in the communities of the higher-temperature sites having typically low *in situ* oxygen, i.e. VWM and DG3 along the drying thermal gradient, and VWM, WG3 and WG4 along the wet thermal gradient.

Metagenomic reads matching genes for the gluconeogenic enzyme phosphoenolpyruvate carboxykinase (PEPCK) were detected in all the mat communities. Number of species-level matches for the PEPCK-related reads increased steadily from 32 in VWM to 102 in DG4, along the drying thermal gradient; and from 32 in VWM to 175 in WG7, along the wet thermal gradient. PEPCK governs the interconversion of carbon-metabolites at the phosphoenolpyruvate–pyruvate–oxaloacetate junction of major chemoorganoheterotrophic pathways, thereby controlling carbon flux among various catabolic, anabolic, and energy-supplying processes^59^. Variations in the number of species-level matches for PEPCK-related reads, therefore, were considered to be reflective of a steady rise in organoheterotrophic inputs in community productivity along both the hydrothermal gradients.

In view of the considerable abundance of dissolved sulfide, elemental sulfur, thiosulfate and sulfate in *Shivlinga*’s vent-water, occurrence and phylogenetic diversity of two key sulfur-chemolithotrophic genes, namely, the thiol esterase-encoding *soxB* of bacteria and the thiosulfate:quinone oxidoreductase-encoding *tqoAB* of archaea^60^, were investigated in the metagenomes of all the mat communities. While *tqoAB* was not present in any metagenome, *soxB* was detected in all of them. Number of species-level matches for *soxB*-related reads increased steadily from 4 in VWM to 16 in DG4, along the drying thermal gradient; and from 4 in VWM to 39 in WG7, along the wet thermal gradient. Sox-based sulfur-chemolithotrophs being predominantly aerobic^60^, these trends were consistent with the increase in oxygen tension along the vent-to-apron trajectories of the hydrothermal gradients.

Metagenomic reads matching genes encoding cytochrome *c* oxidase subunit 1 (*cox1*), the key enzyme of aerobic respiration^61^, are widespread in the *Shivlinga* ecosystem. Number of species-level matches for *cox1*-affiliated reads increased progressively from 32 in VWM to 193 in DG4, along the drying thermal gradient; and from 32 in VWM to 267 in WG7, along the wet thermal gradient. Metagenomic reads matching genes encoding dissimilatory sulfite reductase alpha and beta subunits (*dsrAB*), which is central to the energy-conserving reduction of sulfite to sulfide in anaerobic sulfite/sulfate-reducing prokaryotes^62^, were detected only in some of the mat communities. The number of species-level matches for *dsrAB* reads were 6, 0 and 3 in VWM, DG3 and DG4, respectively; and 6, 9, 17, 0, 2 and 16 in VWM, WG3, WG4, WG5, WG6 and WG7, respectively. No gene for N-oxide (NO/N_2_O) or nitrate (NO_3_) reduction was found in any mat community. Collectively, these data suggest that *Shivlinga*’s mat communities are adept in utilizing whatever low oxygen is there in their watery to semi-watery habitat. This hypothesis is further supported by the considerable occurrence and diversity (across the thermal gradients) of genes involved in the biogenesis of *cbb3* cytochrome oxidase, which is known to be instrumental in respiration under very low oxygen tension^63^. For instance, the number of species-level matches for metagenomic reads ascribable to genes encoding the *cbb3* cytochrome oxidase biogenesis protein CcoG^64^ increased progressively from 0 in VWM to 44 in DG4, along the drying thermal gradient; and from 0 in VWM to 87 in WG7, along the wet thermal gradient.

## Discussion

### An extreme yet highly habitable ecosystem

Diverse lines of evidence indicated that many of *Shivlinga*’s native microorganisms can bypass thermodynamic hurdles to habitation. Phylogenetic relatives of a wide variety of mesophilic bacteria colonizing the high-temperature locations, presence of psychrophilic bacteria at 33–52 °C sites, localized but dense populations of diatoms within the mat communities of 52 °C site, and occurrence of established populations of mosses, fungi, green and red algae, and slime mold at sites of >50 °C, collectively indicate that this ecosystem is highly habitable, biodiverse and complex. The taxonomically diversified and biomass-dense microbiome of this otherwise extreme site (thermal stress, high UV, low pressure) is further complemented by the abundance of taxa at temperatures below their recognized minima for growth, and the abundance of taxa that are ubiquitous throughout the ecosystem irrespective of temperature. For instance, the genera *Chloroflexus*, *Roseiflexus*, *Sulfurihydrogenibium*, *Synechococcus* and *Thermus* remained prevalent over wide ranges of temperature along both the gradients; these genera are known to be physiologically interdependent. *Sulfurihydrogenibium* scavenges dissolved oxygen from hot spring waters, thereby generating anoxic condition for organisms such as *Chloroflexus* and *Roseiflexus* that, under anaerobic conditions in the presence of light, harvest energy via photoheterotrophy and switch back to chemoheterotrophy when it is dark^65^. At the same time, *Synechococcus*, by virtue of producing glycolate and fermenting glycogen, has been shown to promote photoheterotrophic growth of *Roseiflexus* in laboratory mixed cultures^66^. *Roseiflexus*, in turn, is capable of synthesizing glycosides and wax esters that can be utilized for gluconeogenesis by other members of the community^67^.

Studies of microbial ecology in other extreme habitats have characterized many interactions between bacteria and eukaryotic microbes which boost the metabolism and enhance the stress biology of one or more partners^54,68^. For instance, fungal (and bacterial) saprotrophs can make nutrients available to other microbes, many microbes may co-metabolize complex substrates, and photosynthetic bacteria and algae may release compatible solutes to be used by other microorganisms of the community. So, despite the relatively small numbers of eukaryotes in the *Shivlinga* habitat, we believe that the ecological success of bacteria (as reflected by their >97% relative abundance in all mat communities) is reliant, in part at least, on the former. The presence of the moss *Physcomitrella* in the *Shivlinga* microbiome, with highest relative abundance at the 66 °C site, is also noteworthy. Worldwide, mosses are known to colonize geothermal sites which are characterized by distinct temperature and moisture regimes. Substratum temperatures of up to 75 °C have been recorded at depths of 2.5–5 cm beneath mosses, and temperatures of up to 60 °C have been recorded on moss-covered surfaces^69,70^. Like fungi, mosses also exhibit a preference for vegetative propagation (over sexual reproduction) to conserve adaptations to environmental challenges such as high temperature^71,72^. We could not find any evidence for either the sexual stage (the diploid sporophyte) or the intermediate stage (the haploid gametophyte) of *Physcomitrella* at the *Shivlinga* site. We suspect, therefore, that *Physcomitrella* exists within the *Shivlinga* mats only in its plesiomorphic (ancestral) form, which is characterized by algae-like, thalloid structures that do not have diverse tissue types and are known as juvenile gametophytes^49^. This plesiomorphic form is made up of microscopic filaments known as protonema that are formed by chains of haploid cells^49^. A number of studies have shown that *Physcomitrella* efficiently renders homologous recombination-based error-free DNA damage repair, an attribute that is likely to confer adaptive advantage in high-temperature habitats that are generally associated with higher rates of DNA damage^73^. Together with a plausible vegetative/asexual lifestyle, error-free DNA damage repair capabilities can act to reduce genetic variability in *Physcomitrella* and, ultimately, slow down evolution via conservation of genomes^74,75^. These attributes may help this bryophyte to colonize *Shivlinga* via retention of the plesiomorphic form (a phenomenon termed stasigenesis).

### Phylogenetic relatives of mesophilic genera in *Shivlinga*’s high-temperature sites

When all the genus-level annotation data obtained via searching the metagenomes against the nr protein sequence and RDP 16 S rRNA gene sequence databases were collated, at least 26 genera were confirmed as present in every mat community that was sampled (Supplementary Tables 31 and 32), while at least 18 additional genera were ubiquitous along the wet, but not the drying, thermal gradient (Supplementary Table 32). It is remarkable that these bacteria have colonized the entire *Shivlinga* system despite the various thermodynamic constraints (see above). Furthermore, these genera are not all known to encompass thermophilic members, and many of them, for example *Clostridium*, *Lactobacillus*, *Pseudomonas* and *Salinibacter*, are archetypal ‘weeds’ that have a robust stress biology and can maintain dominant positions within microbial communities^76^. The *Shivlinga* site may, therefore, provide a useful model system for future studies of microbial weed ecology under hydrothermal conditions.

In the genus-level analyses of the metagenomic sequence data, a considerable number of the genera were also found to be present at temperatures outside their windows for growth *in vitro*. For instance, entities affiliated to at least 11 genera of typical thermophiles were detected at temperatures below the minima recognized for laboratory growth of the cultured strains of those genera (Supplementary Table 33). These were *Dictyoglomus*, *Hydrogenobaculum*, *Meiothermus*, *Persephonella*, *Petrotoga*, *Sulfurihydrogenibium*, *Thermodesulfatator*, *Thermodesulfobacterium*, *Thermotoga*, *Thermus* and *Thermosipho*. Conversely, phylogenetic relatives of at least 40 such bacterial genera were present in mat communities at >50 °C, no cultured members of which have any report of laboratory growth at >45 °C (see Table 2 and its references given in Supplementary references). The composition of the vent-water community was also consistent with this finding. For instance, no strain of four out of the 12 genera identified in the 70 °C VW community (*Achromobacter*, *Alcaligenes*, *Paenibacillus* and *Propionibacterium*) grow at >45 °C, according to *in vitro* studies. Moreover, strains of some of the reportedly mesophilic genera detected at the high-temperature ends of *Shivlinga*’s hydrothermal gradients - for example, *Dolichospermum*, *Flavobacterium*, *Magnetospirillum*, *Planktothrix*, *Prochlorothrix*, *Thiohalocapsa*, *Treponema* and *Xylella* - are not even known to grow in the laboratory at temperatures >30 °C. Interestingly, psychrophilic microbes such as *Chryseobacterium* and *Nitrosomonas*^77^ were present in all the mat communities except VWM and WG3, and their frequencies increased with decrease in temperature. We conclude, therefore, that the *Shivlinga* hot spring system is not dominated by thermophiles, but hosts an ecophysiologically diversified microbiome that includes several phylogenetic relatives of mesophilic taxa.

**Table 2:** Microbial genera^1^ that are comprised primarily of mesophilic strains (no laboratory growth reported at >45 °C) and phylogenetic relatives of which were detected in *Shivlinga*’s mat communities growing at >50 °C.

Phyla	Genera	Highest temperature at which culture-based growth has been reported	Reference^2^ pertaining to the highest temperature at which culture-based growth was reported	VWM (66 °C)	DG3 (52 °C)	DG4 (41 °C)	WG3 (56 °C)	WG4 (46 °C)	WG5 (38 °C)	WG6 (36 °C)	WG7 (33 °C)
*Bacteroidetes*	*Alistipes*	45 °C	Rautio *et al*. (2003)	**+**	**+**	**+**	**+**	**+**	**−**	**+**	**+**
	*Cytophaga*	35 °C	Christensen (1980)	**+**	**+**	**+**	**−**	**+**	**+**	**+**	**+**
	*Hymenobacter*	40 °C	Subhash *et al*. (2014)	**−**	**+**	**+**	**+**	**+**	**+**	**+**	**+**
	*Flexithrix*	40 °C	Hosoya and Yokota (2007)	**−**	**+**	**+**	**+**	**+**	**+**	**+**	**+**
	*Bacteroides*	45 °C	Hatamoto *et al*. (2014)	**−**	**−**	**+**	**+**	**+**	**−**	**+**	**+**
	*Porphyromonas*	37 °C	Shah and Collins (1988)	**−**	**−**	**+**	**+**	**+**	**−**	**+**	**+**
	*Flavobacterium*	30 °C	Bernardet and Bowman (2015)	**−**	**+**	**−**	**+**	**+**	**+**	**+**	**+**
*Actinobacteria*	*Streptomyces*	42 °C	Han *et al*. (2015)	**−**	**+**	**+**	**+**	**+**	**+**	**+**	**+**
	*Propionibacterium*^3^	45 °C	Koussemon *et al*. (2001)	**−**	**+**	**−**	**+**	**+**	**−**	**−**	**+**
	*Actinoplanes*	37 °C	Sun *et al*. (2009)	**+**	**+**	**−**	**+**	**+**	**−**	**−**	**−**
	*Enterorhabdus*	40 °C	Clavel *et al*. (2010)	**−**	**−**	**−**	**+**	**+**	**−**	**−**	**−**
	*Kitasatospora*	42 °C	Zhang *et al*. (1997)	**−**	**+**	**−**	**+**	**+**	**−**	**−**	**+**
*Proteobacteria*	*Pseudomonas*	41 °C	Wang *et al*. (2016)	**+**	**+**	**+**	**+**	**+**	**+**	**+**	**+**
	*Acinetobacter*	44 °C	Rooney *et al*. (2016)	**−**	**+**	**−**	**−**	**+**	**+**	**+**	**−**
	*Thiobacillus*	42 °C	Brinkhoff *et al*. (1999)	**+**	**−**	**−**	**+**	**−**	**−**	**−**	**−**
	*Acidithiobacillus*	45 °C	Kelly and Wood (2000)	**+**	**+**	**−**	**−**	**+**	**−**	**−**	**−**
	*Campylobacter*	42 °C	Koziel *et al*. (2014)	**+**	**+**	**−**	**+**	**+**	**−**	**−**	**+**
	*Thiohalocapsa*	30 °C	Anil Kumar *et al*. (2009)	**+**	**−**	**−**	**−**	**−**	**−**	**−**	**−**
	*Xanthomonas*	45 °C	Rautio *et al*. (2003)	**−**	**−**	**+**	**+**	**+**	**−**	**+**	**+**
	*Helicobacter*	35 °C	Christensen (1980)	**−**	**+**	**+**	**+**	**+**	**+**	**+**	**+**
	*Magnetospirillum*	30 °C	Schleifer *et al*. (1991)	**−**	**−**	**−**	**+**	**+**	**−**	**−**	**+**
	*Comamonas*	44 °C	Chang *et al*. (2002)	**−**	**−**	**−**	**+**	**−**	**−**	**−**	**−**
	*Neisseria*	42 °C	Wolfgang *et al*. (2011)	**−**	**−**	**−**	**+**	**−**	**−**	**−**	**−**
	*Halochromatium*	35 °C	Imhoff *et al*. (1998)	**−**	**−**	**−**	**+**	**−**	**−**	**−**	**−**
	*Halorhodospira*	35 °C	Hirschler-Rea *et al*. (2003)	**−**	**−**	**−**	**+**	**+**	**−**	**−**	**−**
	*Thiohalospira*	35 °C	Sorokin *et al*. (2008)	**−**	**−**	**−**	**+**	**−**	**−**	**−**	**−**
	*Xylella*	28 °C	Wells *et al*. (1987)	**−**	**−**	**+**	**+**	**+**	**−**	**+**	**+**
	*Marichromatium*	35 °C	Sucharita *et al*. (2010)	**−**	**−**	**−**	**+**	**+**	**−**	**−**	**−**
	*Paracoccus*	40 °C	Kim *et al*. (2010)	**−**	**+**	**+**	**−**	**−**	**−**	**+**	**+**
	*Methylobacterium*	37 °C	Wood *et al*. (1998)	**−**	**+**	**−**	**−**	**−**	**+**	**+**	**+**
*Cyanobacteria*	*Gloeobacter*	37 °C	Castenholz (2001)	**−**	**−**	**−**	**+**	**+**	**−**	**−**	**−**
	*Cylindrospermopsis*	35 °C	Briand *et al*. (2004)	**−**	**−**	**−**	**+**	**−**	**−**	**−**	**−**
	*Nostoc*	25 °C	Castenholz (2001)	**−**	**−**	**+**	**+**	**+**	**+**	**−**	**+**
	*Prochlorothrix*	30 °C	Castenholz (2001)	**−**	**+**	**−**	**+**	**+**	**+**	**+**	**+**
	*Dolichospermum*	30 °C	Castenholz (2001)	**−**	**+**	**−**	**+**	**+**	**−**	**+**	**+**
	*Planktothrix*	30 °C	Castenholz (2001)	**−**	**+**	**−**	**+**	**+**	**−**	**+**	**+**
*Spirochaetes*	*Treponema*	30 °C	Evans *et al*. (2009)	**+**	**+**	**+**	**−**	**+**	**+**	**+**	**+**
	*Leptospira*	37 °C	Smythe *et al*. (2013)	**−**	**+**	**+**	**+**	**+**	**+**	**+**	**+**
*Tenericutes*	*Acholeplasma*	37 °C	Tully *et al*. (1994)	**+**	**−**	**+**	**−**	**−**	**−**	**+**	**+**
*Firmicutes*	*Streptococcus*	45 °C	Sherman *et al*. (1931)	**+**	**−**	**+**	**−**	**+**	**−**	**−**	**+**
	*Paenibacillus*^3^	37 °C	Shida *et al*. (1997)	+	**−**	**−**	+	+	+	+	+

^1^Identified in RDP searches of the metagenomes.

^2^References used in this table have been given in Supplementary References.

^3^These genera were also identified in the V3 sequence-based OTU analysis of the VW community.

### Insights into the biophysics of the *Shivlinga* ecosystem

Extremes of temperature constrain microbial function at the level of macromolecules, the cell system, ecosystems, and the biosphere. The ecology of *Shivlinga’s* microbiome is undoubtedly influenced by the high temperature. Interestingly, however, and although microbial diversity decreases at around 50 and 60 °C, several phyla/genera inhabit locations having temperatures above their *in vitro* growth limits. This is analogous to the habitability of otherwise hostile chaotropic brines (>2.50 M MgCl_2_) that is facilitated by kosmotropic (compensatory) effects of sodium and sulfate^5,7^. Although *Shivlinga* is a high-temperature and low-pressure habitat for microbes, its spring-water and sinters contain high concentrations of borates, sodium, bicarbonate, thiosulfate and sulfate. In addition, the system has lower, but significant, concentrations of sulfite, sulfide and lithium. Borates, sodium, bicarbonate, thiosulfate, sulfate, sulfite, and sulfide are kosmotropic^11^, and it is possible that their cumulative kosmotropic activity enables colonization of high-temperature sites by the mesophilic taxa, a phenomenon which parallels the way in which kosmotropes facilitate colonization of chaotropic environments^5,7,10,19^. We believe that the kosmotropic ions within the *Shivlinga*’s system might also enhance biotic activity of the thermotolerant microbes present.

## Methods

### Site description

Within the Puga geothermal area, which is a part of the greater Ladakh-Tibet borax-spring zone^44^, surface expressions of geothermal activity, including the *Shivlinga* microbialite spring (GPS coordinates: 33 ° 13′ 46.2″ N and 78 ° 21′ 18.7″ E), are largely confined to the eastern part of the 15 km-long and 1 km-wide east-west orientated valley. Information on the geography, geology, and geothermal activity of Puga can be found in Supplementary Note 1. Of the number of distinct axes of *Shivlinga*'s spring-water transit/dissipation, two trajectories representing a progressively-drying, and a wet, thermal gradient were investigated for their geomicrobiological characteristics. Collation of the microbiological and geochemical data revealed three distinct geomicrobiological zones within the *Shivlinga* system. The first geomicrobial zone of the *Shivlinga* microbiome, ***the vent and the microbialite body*** together form a unit that is elevated above the ground level. The chimney-shaped microbialite is made up of hardened hydrothermal mineral deposits (sinters); the water vented from within the microbialite, at the time of sampling (23 July 2013), was characterized by 70 °C temperature, pH 7.0, and 25 mL s^−1^ flow-rate. The vent-water community (VW) was sampled from the vent opening located at the microbialite summit (Fig. 1C,I). A microbial mat is anchored to the inner surface of the vent’s rim for the entirety of its circumference, and has grown inwards across the surface of the water. Most of the filamentous structures that form this vent-water mat (VWM) are white, but are interspersed with minute green-hued elements (Fig. 1C,I). VW and VWM represented the initial sample-points for both the wet, and the drying, thermal gradients, for which all subsequently-sampled mat communities were named using the prefixes WG and DG, respectively. Starting from where VWM attached to the sintered rim of the vent, a 1-cm-thick, green mat containing occasional red patches (community DG3) spread radially over the flat mineral surface that circumvents the opening at the top of *Shivlinga*’s body (Fig. 1C,I). The next community surrounding the vent (sampled as DG4) forms another cm-thick mat which is pigmented purple with a green shimmer; it forms an unbroken ring extending from the DG3 community to the edge of *Shivlinga*’s summit (Fig. 1C,I). In contrast to VWM, DG3 and DG4 are not in contact with a flowing body of water; instead they are located on the moist sinters of the flat summit which surrounds the vent and progressively desiccates towards its outer edge (scraping off sections of the mats revealed no flowing water beneath). VW, VWM, DG3 and DG4 together form a continuum of microbial communities along this drying thermal gradient (Fig. 1C,I). Across a ~10-cm arc of *Shivlinga*’s circular top, the vent-water spills down the south-west-facing side of the microbialite body. The filamentous structures of VWM extend across the rim, along the spring-water flow, and down the side of *Shivlinga* where they were designated as community WG3 and sampled at a point that is 22 cm beneath the summit (Fig. 1C,I). WG3, like VWM, appears white, but is interspersed with apparently greater proportion of green filaments.

***The microbialite’s base (including the sloping bedrock)***, the second geomicrobiological zone of the *Shivlinga* site, has a lower temperature (38–46 °C), lesser flow-rates, pH of ~8, and two morphologically distinct microbial mats. This bedrock, on which the microbialite is situated, is a conical section of sedimentary limestone which protrudes from the regolith. The original surface of this bedrock has been eroded by the spring-water, and coated with new mineral deposits and/or microbial mats. The WG3 streamer, which follows the spring-water down one side of the microbialite body, does not extend beyond the base of *Shivlinga*. Instead, it gives way to a green mat containing occasional yellow patches (community WG4), which in turn is restricted to the first 10’s cms of the slope along the trajectory of the spring-water transit (Fig. 1C,D,I,J). Further along the water flow, a stretch of rust-colored mat (community WG5) covers the rest of the limestone outcrop (Fig. 1D,J).

***The apron*** is the outer (and last) geomicrobiological zone of the *Shivlinga* microbiome; it starts where the slope of the bedrock ends and extends across a circular area of the regolith that is ~1.5 m in radius around the bedrock. Its surface is composed of a loose, heterogeneous mixture of fluvioglacial sediments, aeolian sand, clay and scree which overlay the bedrock. The terrain of the apron is only gently inclined, so the spring-waters (pH ~8.5) flow very slowly (Table 1) along channels in the regolith (these are 1 to 2-cm-deep and 5 to 50-cm-wide) before dissipating into the dry loose valley-fill material. Across the inner half of the apron, the floors of the outflow channels are covered with intensely red nodules (Fig. 1B,E,H,K) formed by the nucleation of hydrothermal minerals onto microbial cells and extracellular organic substances (community WG6). Within the outer half of the apron, 3 to 10 mm-thick tufts of dark grey streamers (community WG7) grow along the outflow (Fig. 1B,F,H,L). VW, VWM, WG3, WG4, WG5, WG6 and WG7 together form a continuum of microbial communities along this wet thermal gradient.

### Sampling and collection of *in situ* data

At the discrete sample-sites within the *Shivlinga* system, temperatures were measured with a mercury-column glass thermometer and pH values were measured using Neutralit indicator strips (Merck, Germany). The discharge rate of *Shivlinga*’s vent-water was determined (in mL s^−1^) using the soluble-tracer dilution technique described previously^78^. Flow rates of spring-water over the sampled mat communities were determined (in cm s^−1^) using a modified insoluble-dye tracer technique^79^.

*Shivlinga*’s vent-water was collected at the same time in separate bottles for chemical and microbiological analyses using separate, 25 mL sterile pipettes for each sampling event. For analyses of water chemistry, VW samples were passed through a 0.22 µm syringe filter. Every 100 mL batch of VW sample meant for the quantification of metallic elements was acidified to pH ≤ 2 by adding 69% (w/v) HNO_3_ (400 μl). To stop all biotic activity within the VW samples meant for bicarbonate estimation, saturated HgCl_2_ solution was added at a ratio of 1:20 (v/v, HgCl_2_:sample). For microbiology, 1000 mL vent-water was passed through a sterile 0.22 µm cellulose acetate filter (Sartorius Stedim Biotech, Germany), following which the filter was put into an 8-mL cryovial containing 50 mM Tris:EDTA (5 mL, pH 7.8) and immediately placed in dry ice.

Every mat community was sampled once for microscopic studies and twice for metagenome analysis: the duplicate sets of metagenomic data generated were used to evaluate the statistical significance of the differences in relative abundance of taxa between communities. For each mat community, in each of the three sampling rounds (one for microscopy and two for metagenomics), fresh and intact material (2.5 cm^2^ and located 5 mm from the other two mat portions sampled) was scraped off using a sterile scalpel, without disturbing the mat’s internal structure or the substratum. The stable structure of the mats and consistent physicochemical conditions of the environment recorded over the years, together with the fresh and intact appearance of the mat samples used for all analyses were consistent with a relative absence of necromass.

Mat samples were put into sterile Petri plates containing 3 mL 70% (v/v) ethanol:acetic acid:formaldehyde (9:1:1 v/v/v) or a 15 mL cryovial containing 5 mL Tris:EDTA (50 mM; pH 7.8), depending on whether they were required for microscopic or metagenomic analysis, respectively. The flakes of precipitating mineral salts were not removed from the mat samples meant for microscopic analysis. Petri plates were sealed with Parafilm (Bemis Company Inc., USA) and packed in polyethylene bags, and the cryovials were sealed using Parafilm and then placed in dry ice. Dry ice in the sample-transportation boxes was replenished *en route* to the laboratory at Bose Institute (Kolkata). Samples destined for metagenomic analysis were stored in the laboratory at −20 °C, while those for microscopic, chemical or mineralogical studies were kept at 4 °C.

### Analyses of vent-water chemistry

Concentrations of boron, calcium, lithium, magnesium, and potassium were determined on a Thermo iCAP Q ICPMS (Thermo Fisher Scientific, USA); standard curves were prepared using ICPMS standards supplied by Sigma Aldrich (USA) and VHG Labs Inc. (USA). Based on replicate analyses of the standards, deviations from actual concentrations were less than 2%, 1%, 2.1%, 1% and 2.5% for boron, calcium, lithium, magnesium and potassium, respectively. Concentrations of sodium were determined using an Agilent 240 AA atomic absorption spectrometer (Agilent Technologies, USA); standard curves were prepared from Sigma Aldrich AAS standards. Based on multiple analyses of the standard, deviations from actual sodium concentrations were <3%. Silicon concentration was determined using a UV-visible spectrophotometer (CARY 100, Varian Deutschland GmbH, Germany), as described previously^80^. Chloride was quantified by precipitation titration with silver nitrate (0.1 N) using a Titrino 799GPT auto titrator (Metrohm AG, Switzerland). Bicarbonate concentrations were calculated from the dissolved inorganic carbon (DIC) content and total alkalinity of the water samples according to Lewis and Wallace^81^. Total alkalinity was determined using the Gran titration method (and the Titrino 799GPT auto titrator), and dissolved inorganic carbon was determined by CM 5130 carbon coulometer (UIC Inc., USA). Thiosulfate and sulfate concentrations in the vent-water were determined by iodometric titration, and gravimetric precipitation using barium chloride, respectively^82,83^. Sulfite was analyzed spectrophotometrically, using pararosaniline hydrochloride (Sigma Aldrich) as the indicator^84^. Immediately after collecting the vent-water samples, dissolved sulfides (ΣHS^−^, which includes H_2_S, HS^−^ and/or S_x_^2−^) were precipitated from them as CdS using cadmium nitrate [Cd(NO_3_)_2_] in crimp-sealed butyl septum bottles leaving no head space. After the bottles were brought back to the laboratory, spectrophotometric measurement of sulfides was carried out based on the principle that N, N-dimethyl-p-phenylene diamine dihydrochloride and H_2_S react stoichiometrically in the presence of FeCl_3_ and HCl to form a blue-colored complex^85^. Sulfate, sulfite and thiosulfate were also quantified in the CdS-precipitated, ΣHS^−^-free VW sample using a Metrohm ion chromatograph (Basic IC plus 883) equipped with a suppressed conductivity detector (Metrohm, IC detector 1.850.9010) and a MetrosepASupp 5 (150/4.0) anion exchange column (Metrohm AG).

### Microscopy of mats and mineralogy of sinters

For phase contrast microscopy or laser scanning confocal microscopy, 1-mm^2^ portions of the mat samples were put onto grease-free glass slides, teased apart gently with sterile needles after adding 50 mM phosphate buffer (pH 7.0) or a few drops of DPX Mountant (Sigma Aldrich), and examined using an BX-50 phase contrast microscope (Olympus Corporation, Japan) or a LSM 510 Meta confocal microscope (Carl Zeiss AG, Germany). Electronic images of the autofluorescence of microbial cells were recorded using confocal microscopy at various excitation and emission-detection wavelengths. To analyze the mineral salts precipitated on the mat samples, the latter were also subjected to EDS following SEM on an FEI Quanta 200 microscope (Field Electron and Ion Company, USA). To give contrast to the biological components of the microbe-mineral assemblages, samples were prefixed with vapors of 1% (w/v) osmium tetroxide in deionized water.

Elemental composition of *Shivlinga*’s sinters was determined via standard inorganic tests, and then EDS, EPMA, and XRD. EDS was carried out using an EDAX system attached to the FEI Quanta 200. Quantitative analyses were carried out and atomic density ratios were derived using the GENESIS software controlling the EDAX. XRD was carried out using an XPert Pro X-ray Diffractometer (PANalytical, the Netherlands) equipped with a copper target, operating at 40 kV and 30 mA, and scanned from 4 to 70° 2θ. For EPMA, grain-mount slides of the sinter materials were prepared and elemental composition analyzed using an SX100 EPMA machine (CAMECA, France); electron beam size of 1 µm, accelerating voltage of 15 kV and current of 12 nA were used in the EPMA. The sinter sample slides were also imaged under the EPMA machine via electron backscattering to identify any encrusted microorganisms.

### Isolation of total environmental DNA from *Shivlinga*’s vent-water

The filter through which 1000 mL of *Shivlinga*’s vent-water was passed during sampling was cut into pieces with sterile scissors, within the 50-mM-Tris:EDTA (pH 7.8)-containing 8 mL cryovial in which it had come from the sample site. The cryovial was vortexed for 30 min, following which the filter fragments were discarded and the remaining Tris:EDTA was distributed equally to five 1.5 mL microfuge tubes. These were centrifuged (10,800 *g* for 30 min at 4 °C), and then 900 μl Tris:EDTA was removed by pipette from the top of each tube and discarded. The remaining Tris:EDTA (100 μl) in each tube was vortexed for 15 min and the contents of all five tubes pooled by placing into a single microfuge tube. The pooled Tris:EDTA (500 μl) was again centrifuged (10,800 *g* for 30 min at 4 °C), following which 400 μl was removed from the top and discarded. The remaining 100 μl contained all of the microbial cells that were present in the original 1000 mL VW sample. DNA was isolated from this 100 μl cell suspension by the QIAamp DNA Mini Kit (Qiagen, Germany), following manufacturer’s protocol.

### Assessment of metataxonomic diversity in the *Shivlinga* vent-water community

V3 regions of all 16S rRNA genes present in the total environmental DNA extracted from the VW sample were PCR-amplified using Bacteria-/Archaea-specific universal oligonucleotide primers and sequenced by Ion Torrent Personal Genome Machine (Ion PGM) (Thermo Fisher Scientific), following the fusion primer protocol described previously^25,26^. For bacterial 16 S rRNA genes, amplification was carried out using the universal forward primer 341f (5′-CCTACGGGAGGCAGCAG-3′) prefixed with an Ion Torrent adapter and a unique sample-specific barcode or multiplex identifier; the reverse primer had a trP1 adapter followed by the universal reverse primer 515r (5′- TATTACCGCGGCTGCTGG-3′). To amplify archaeal 16S rRNA genes, we used the universal forward primer 344 f (5′-AATTGGANTCAACGCCGG-3′) prefixed with an Ion Torrent adapter and a unique sample-specific barcode or multiplex identifier; the reverse primer had a trP1 adapter followed by the universal reverse primer 522r (5′-TCGRCGGCCATGCACCWC-3′). Prior to sequencing, size distribution and DNA concentration within the V3 amplicon pool was checked using a Bioanalyzer 2100 (Agilent Technologies) and adjusted to 26 pM. Amplicons were then attached to the surface of Ion Sphere Particles (ISPs) using an Ion Onetouch 200 Template kit (Thermo Fisher Scientific). Manually enriched, templated-ISPs were then sequenced by PGM on an Ion 316 Chip for 500 flows.

Before retrieval from the sequencing machine, all reads were filtered by the inbuilt PGM software to remove low quality, and polyclonal, sequences; sequences matching the PGM 3′ adaptor were also trimmed. The sequence file was deposited to the NCBI Sequence Read Archive (SRA) with the run accession number SRR2904995 under the BioProject accession number PRJNA296849. Reads were filtered a second time for high quality value (QV 20) and length threshold of 100 bp; OTUs were created at 97% identity level using the various modules of UPARSE^86^, and singletons were discarded. A Perl programming-script, available within UPARSE, was used to determine the Abundance-based Coverage Estimator, and Shannon and Simpson Indices. Rarefaction analysis was carried out using the Vegan Package in R^87^. The consensus sequence of every OTU was taxonomically classified by the RDP Classifier tool located at http://rdp.cme.msu.edu/classifier/classifier.jsp.

### Extraction, sequencing and analysis of metagenomes from the mat communities

Total community DNA (metagenome) was extracted separately from each of the duplicate samples available for all the mat communities using PowerMax Soil DNA Isolation Kit (MoBio, Carlsbad, CA, USA). Deep-shotgun sequencing of the 16 metagenomes obtained was carried out as described previously^25^. The Ion PGM or the Ion Proton platforms (Thermo Fisher Scientific) were employed for this purpose, using 400-bp read chemistry on the Ion 318 chip or 200-bp read chemistry on the PI V2 chip, respectively. All metagenomic readsets (mean read-length 138–231 nucleotides) were deposited to the NCBI SRA under the BioProject PRJNA296849 with the run accession numbers provided in Supplementary Tables 4 and 5.

The two metagenomic readsets sequenced from the sample replicates of each mat community were annotated separately by searching against the nr protein sequence database of NCBI, using the Organism Abundance tool of MG-RAST^88^. In the process, two independent values were obtained for the relative abundance of every taxon within a community. Subsequently, mean relative abundance of each taxon was calculated using the duplicate values, and then used for comparisons between communities along the hydrothermal gradients. To determine the significance of population fluctuation for a taxon between community ‘x’ and community ‘y’, its mean relative abundance values in ‘x’ and ‘y’ were compared in combination with the actual ranges of the data (i.e. the independent relative abundance values). From ‘x’ to ‘y’, relative abundance of a taxon was considered to have increased significantly when the lower range of its relative abundance in ‘y’ was greater than the upper range of the corresponding value in ‘x’. Similarly, a taxon was considered to have declined significantly from ‘x’ to ‘y’ when the upper range of its relative abundance in ‘y’ was smaller than the lower range of the corresponding value in ‘x’. In this way it could be ascertained whether the inferred relative abundance fluctuations were significant.

Within the MG-RAST pipeline, sequences were trimmed so as to contain no more than five bases below the Phred quality score of 15; taxonomic classification of reads was carried out using the Organism Abundance tool following the Best Hit Classification algorithm. For classification down to the phylum-, class- or genus-level, readsets were searched by BlastX against the nr protein sequence database with a minimum alignment length of 45 bp (15 amino acids) and a minimum identity cut-off point of 60%. Percentage allocation of metagenomic reads to individual taxa (whether at the phylum-, class- or genus-level) was considered as a direct measure of the relative abundance of those taxa within the community in question^25,89^. Furthermore, to corroborate the genus-level identifications, each readset was searched for 16 S rRNA genes against the RDP database using BlastN with minimum alignment length of 50 bp and 70% minimum identity cut-off. A read was assigned to a genus only when it shared >94% 16S rRNA sequence identity with a known species of that genus. Maximum e-value cut-off used in all the above analyses was 1e^−5^. Matching of DG3 sample-derived metagenomic reads with the 16 S rRNA gene sequences (FN556455 through FN556457) of some mesophilic *Paracoccus* strains isolated previously from DG3-equivalent (52 °C) *Shivlinga* mat samples validated that the above method was efficient in not only detecting microorganisms that are present in small quantities but also adept to classifying metagenomic reads up to the genus level. This procedure also corroborated that *Shivlinga*’s microbial communities, including the mesophilic components, were structurally stable over time.

### Statistical analysis of associations between variables

Pair-wise Pearson correlation coefficients (*r*) were determined to quantify the level of association between variations in the mean relative abundances of individual phyla along the wet thermal gradient and the *in situ* parameters such as temperature, pH, distance from the vent and flow-rate. In these single-inference statistical procedures, a confidence level corresponding to *P *value < 0.05 was considered as indicative of significant correlation between a pair of variables – this implied that there was a probability of 0.05 for the null hypothesis to be rejected mistakenly and the inference to be actually insignificant. However, these confidence levels (*P* values) were applicable to the individual statistical tests and could not be considered simultaneously in multiple-comparison procedures involving individual families of tests (such as those for individual phyla versus temperature), which were desirable in the current study. Towards the latter end, *P* values were subjected to correction for multiple testing using Benjamini-Hochberg method for controlling false discovery rate^90^; this was carried out separately for mean relative abundances of individual phyla along the wet thermal gradient versus temperature, pH, distance from vent center, or flow rate. Benjamini-Hochberg critical value was determined as (*i* ÷ *m*) * *Q* where *i* = rank of the individual *P* value, *m* = total number of tests, and *Q* = the false discovery rate (presently taken as 20%).

### Quantification of microbial diversity in the mat communities using metagenomic data

Microbial diversity within each mat community was estimated, as described previously^25^, using the mean relative abundance value for each bacterial phylum/proteobacterial class present to calculate Simpson Dominance and, Shannon–Wiener Diversity and Evenness Indices^91^. First, to quantify the extent to which the different bacterial phyla/proteobacterial classes dominated a given community, Simpson Dominance Index was determined using Eq. 1 (below). Here, the n_i_/n ratio, denoted as p_i_, gave the proportion of representation of the i^th^ bacterial phylum/proteobacterial class in the community, while S denoted the total number of such taxa present. The mean percentage of all metagenomic reads ascribable to a bacterial phylum/proteobacterial class (Supplementary Tables 34 and 35) was taken as its n_i_/n value (Supplementary Tables 13–20). Shannon Diversity Index (*H*) was calculated using Eq. 2 (below): here, the p_i_ [or the (n_i_ / n)] value of each phylum was taken as above; then each p_i_ value was multiplied by its natural logarithm (Ln p_i_); in the end, the (p_i_ X Ln p_i_) products obtained for all the bacterial phyla and proteobacterial classes were summed-up and multiplied by −1. In order to determine whether there is evenness within a community in terms of the prevalence (relative abundance) of bacterial phyla/proteobacterial classes, Shannon Equitability Index (*E*_*H*_) was calculated using Equation 3 (below). *E*_*H*_ was determined by dividing the *H* value of the community by *H*_max_, which is known to be equal to Ln S (S denoted the total number of phyla and proteobacterial class present).

1 $$D=\mathop{\sum }\limits_{{\rm{i}}=1}^{{\rm{S}}}{\left(\frac{{{\rm{n}}}_{{\rm{i}}}}{{\rm{n}}}\right)}^{2}=\sum \,{p}_{{\rm{i}}}^{2}$$

2 $$H=-\,\mathop{\sum \,}\limits_{{\rm{i}}=1}^{{\rm{s}}}{{\rm{p}}}_{{\rm{i}}}\,\mathrm{Ln}\,{{\rm{p}}}_{{\rm{i}}}$$

3 $${E}_{H}=\frac{H}{{H}_{max}}=\frac{H}{Ln\,S}$$

### Functional analysis of the mat metagenomes

For each mat community, a complete gene catalogue was prepared by searching, and functionally annotating, its merged metagenomic readset against the EggNOG (evolutionary genealogy of genes: Non-supervised Orthologous Groups) database^92^. Genes ascribable to Clusters of Orthologous Groups (COGs) of Proteins^93^ were shortlisted from the catalogue, and the COG-counts under individual functional categories were determined for the community. Whether individual COG-counts under different functional categories across the eight communities were significantly high or low was determined by Chi Square test. The contingency table for this test (Supplementary Table 36) was constructed with the help of an *in house* script (*P* value < 0.001 was used as the cut-off for estimating whether attendance of COGs within a functional category was high or low for a community). Here, pair-wise differential COG-count for two functional categories was placed in two rows (with a degree of freedom = 1) in the 2x2 contingency table and the Chi Square significance test performed for the two individual communities. Furthermore, hierarchical clustering^94^ was carried out to quantitatively decipher the relatedness between the mat communities in terms of their enrichment of various COG categories. Heat map depicting the results of hierarchical cluster analysis was drawn with an *in house* R script (Supplementary Note 3) using complete linkage method (Johnson’s maximum method)^95^.

In addition to the above analyses, diversities of ecophysiologically important functional genes within individual mat communities were separately determined by searching their total metagenomic reads against the Protein Subsystems database using the Functional Abundance tool and Hierarchical Classification algorithm of MG-RAST^88^ [with minimum alignment length of 45 bp (15 amino acids) and minimum identity cutoff of 60%]; total number of species-level matches for each of the relevant functional genes was also identified. In this way, diversities of true thermophiles (organisms that grow in the laboratory exclusively at ≥80 °C), autotrophs, heterotrophs, sulfur-chemolithotrophs, aerobes, and anaerobes were estimated within each community, and then compared across the hydrothermal gradients.

## Supplementary information

Supplementary Information.

## Data Availability

All sequence data sets generated under this study are available in the NCBI SRA repository under the BioProject accession number PRJNA296849 (https://www.ncbi.nlm.nih.gov/sra).
