# Supplementary material for: Microbiome and ecology of a hot spring-microbialite system on the Trans-Himalayan Plateau
Source: Sci Rep. 2020 Apr 3;10:5917. doi: 10.1038/s41598-020-62797-z (PMC7125080; doi:10.1038/s41598-020-62797-z)
Supplement: Supplementary file 1 — Supplementary Information. [file 41598_2020_62797_MOESM1_ESM.docx]

**Supplementary Information for the paper**

**Microbiome and ecology of a hot spring-microbialite system on the Trans-Himalayan Plateau**

Chayan Roy^1^, Moidu Jameela Rameez^1^, Prabir Kumar Haldar^1^, Aditya Peketi^2^, Nibendu Mondal^1^, Utpal Bakshi^3^, Tarunendu Mapder^4^, Prosenjit Pyne^1^, Svetlana Fernandes^2^, Sabyasachi Bhattacharya^1^, Rimi Roy^1,$^, Subhrangshu Mandal^1^, William Kenneth O’Neill^5^, Aninda Mazumdar^2^, Subhra Kanti Mukhopadhyay^6^, Ambarish Mukherjee^7^, Ranadhir Chakraborty^8^, John Edward Hallsworth^5^ and Wriddhiman Ghosh^1^*

**Addresses:**

^1^ Department of Microbiology, Bose Institute, P-1/12 CIT Scheme VIIM, Kolkata 700054, India.

^2^ Gas Hydrate Research Group, Geological Oceanography, CSIR-National Institute of Oceanography, Dona Paula, Goa - 403004, India.

^3^ Microbiome Program, Center for Individualized Medicine, Mayo Clinic, Rochester, MN-55905, USA.

^4^ ARC CoE for Mathematical and Statistical Frontiers, School of Mathematical Sciences, Queensland University of Technology, Brisbane, QLD 4000, Australia.

^5^ Institute for Global Food Security, School of Biological Sciences, Queen’s University Belfast, 19 Chlorine Gardens, Belfast, BT9 5DL, Northern Ireland.

^6^ Department of Microbiology, University of Burdwan, Burdwan, West Bengal 713104, India.

^7^ Department of Botany, University of Burdwan, Burdwan, West Bengal 713104, India.

^8^ Department of Biotechnology, University of North Bengal, Siliguri, West Bengal 734013, India.

**Present Addresses:**

^$^ Department of Botany, Jagannath Kishore College, Purulia - 723101, West Bengal, India.

*** Correspondence: Email:** wriman@jcbose.ac.in; Wriman@rediffmail.com

**Phone:** +91-33-25693246

**Fax:** +91-33-23553886

### Contents

**Supplementary Notes**

**Supplementary Note 1.** Geography, geology and geothermal activity of the study-site.

**Supplementary Note 2.** Phylogenomic diversity analysis for the individual *Shivlinga* mats.

**Supplementary Note 3.** The R script used to draw the heat map depicting the results of hierarchical cluster analysis.

**Supplementary Figures**

**Supplementary Fig. 1. Location of Puga Valley in eastern Ladakh.** (**A**) map showing the location of Leh, the **capital of the union territory of Ladakh** (red shape numbered as 1), and Puga Valley (red shape numbered as 2); (**B**) satellite image showing the position of *Shivlinga* (red shape numbered as 1) at the eastern extremity of Puga Valley, in context with the nearest village Sumdo (red shape numbered as 2) and the road that leads to Puga Valley from Leh (indicated by green arrows), running all along the Indus river and veering south across the river just before the village of Mahe (red shape numbered as 3); (**C**) zoomed-in satellite view of the eastern-extremity of Puga Valley showing the *Shivlinga* site and the Sumdo village. A, B and C, were sourced from the publicly available databases http://maps.google.com/ and http://earth.google.com/, respectively.

**Supplementary Fig. 2.** Photographic monitoring of the mineralization process over a mat portion situated at the margin of the spring-water flow, on the upper surface of the vent’s rim: (**A**) picture taken on Day 1, thin layers of white mineral covering the green mat; (**B**) picture taken on Day 7, soft white spherules and shrub-like bodies of accreted minerals cover the mat (minute green bodies can be seen protruding out of the spherules); (**C**), picture taken on Day 14, further growth of the green mat over the white mineral deposits; (**D**), picture taken on Day 21, the green mat grows completely out of the mineral covering. The green scale bar (= 10 mm) shown in panel C applies to all four panels.

**Supplementary Fig. 3.** Rarefaction curve showing the proportionality between OTU-level metataxonomic diversity revealed and the number of amplified 16S rRNA gene sequence (V3 region) reads analyzed for the sample VW. Number of reads used in OTU building are plotted along the X-axis, while number of OTUs (including singletons) created at the 97% sequence identity level are plotted along the Y-axis.

**Supplementary Fig. 4. Fluctuations in the relative abundance of bacterial phyla**/**proteobacterial classes along the drying thermal gradient.** For each phylum/class within a community, its mean relative abundance has been plotted alongside the two original relative abundance values obtained from the duplicate metagenomes (shown as vertical range bar). To compare the widely unequal relative abundance values comprehensively, their log to the base 10 are plotted along the Y-axis. Only those taxa which had > 1% read share in at least one of the six metagenomic readsets analyzed are shown.

**Supplementary Fig. 5. Fluctuations in the relative abundance of bacterial phyla**/**proteobacterial classes along the wet thermal gradient.** For each phylum/class within a community, its mean relative abundance has been plotted alongside the two original relative abundance values obtained from the duplicate metagenomes (shown as vertical range bar). In order to compare the widely unequal relative abundance values comprehensively, their log to the base 10 are plotted along the Y-axis. Only those taxa which had > 1% read share in at least one of the twelve metagenomic readsets analyzed are shown.

**Supplementary Tables**

**Supplementary Table 1.** Maximum and minimum temperature, pH, and flow-rate recorded at the *Shivlinga* sample-sites during explorations prior to July 2013.

**Supplementary Table 2.** Elemental composition of sinters deposited on the top, sides, interior, base, and apron, of *Shivlinga*.

**Supplementary Table 3.** Summary statistics of metataxonomic analysis of the *Shivlinga* vent water community (VW).

**Supplementary Tables 4 and 5.** Summary of metagenomic investigation of the mat communities of the drying, and the wet, thermal gradients, respectively.

**Supplementary Table 6-12.** Pair-wise Pearson correlation coefficients (CC) calculated between the fluctuations in the relative abundances of individual phyla/classes along the wet thermal gradient and, variations in temperature, pH, distance from the vent, or flow-rate of the spring-water, along the same trajectory. CC values (*r*) which were significant according to Benjamini-Hochberg-corrected probability (*P*) values are shown in Table S5. Calculations for all the CC, and corresponding Benjamini-Hochberg-corrected P, values are shown in Tables S6-S11.

**Supplementary Tables 13-20.** Calculation of ecological indices for VWM, DG3, DG4, WG3, WG4, WG5, WG6 and WG7, respectively, using phylum-/class-level distribution of shotgun metagenomic reads.

**Supplementary Table 21.** Genera collectively accounting for average 50% of all classifiable read (after searching against the nr protein sequence database) in the metagenomes of the mat communities VWM, DG3 and DG4.

**Supplementary Table 22.** Genera collectively accounting for average 50% of all classifiable read (after searching against the nr protein sequence database) in the metagenomes of WG3, WG4, WG5, WG6 and WG7.

**Supplementary Tables 23-30.** Total number of genera identified in VWM, DG3, DG4, WG3, WG4, WG5, WG6 and WG7, respectively, after searching their duplicate metagenomic readsets against the RDP database.

**Supplementary Tables 31 and 32.** Microbial genera that were detected in all the mat communities of the progressively-drying, and the wet, thermal gradients, respectively.

**Supplementary Table 33.** Microbial genera comprised primarily of thermophilic species but present even at low-temperature (33-46°C) sites.

**Supplementary Tables 34 and 35.** Phylum- or class-level percentage distribution of the total bacterial metagenomic reads identified in the mat samples of the drying, and the wet, thermal gradients respectively.

**Supplementary Table 36.** The contingency table that was used to determine by Chi-square test whether individual COG-counts under different functional categories^1^ across the eight communities were significantly high or low.

**Supplementary References**

Supplementary references used in Table 2 of the main text and the Supplementary Table 33.

**Supplementary Notes**

**Supplementary Note 1**

**Geography, geology, and geothermal activity of the study-site**

The Puga geothermal area, which is a part of the greater Ladakh-Tibet borax-spring zone (Harinarayana *et al*., 2006), is located in the Eastern part of the Ladakh district of Jammu and Kashmir, the northern-most state of India. It is connected to the district headquarter Leh by a 180 km metalled road, which runs along the river Indus for the first ~150 km from Leh before veering south across the Indus to reach the valley of Puga (average altitude ~4500 m from the sea level). Puga is situated just south of the tectonically-active collision junction between the Indian and Asian continental crusts involved in Himalayan orogeny (Gansser, 1964). All the surface expressions of geothermal activity, including *Shivlinga*, are restricted to the eastern part of the 15 km-long and 1 km-wide east-west orientated valley. While several lines of geochemical evidence suggest a direct association of the Puga hot springs with underlying magmatic sources (Chowdhury *et al*., 1974; Chowdhury *et al*., 1984; Saxena and D’Amore, 1984; Shanker *et al*., 1999), geophysical studies show the subsurface to be underlain by two geothermal reservoirs at two sequential depths (Harinarayana*et al*., 2006, Azeez and Harinarayana, 2007). A shallow heat-reservoir (having temperature ~160°C) is present at a depth of ~450 m, in the fractured basement rocks (breccia) saturated with hot water. The main geothermal reservoir (having temperature up to 260°C) commences at a depth of ~2 km and extends vertically across the upper Himalayan crust up to a depth of ~8 km. Beneath these levels, i.e. in the mid-to-lower Himalayan crust, lie the magma chambers or partial melts, which resulted from the high pressures and temperatures generated from the collision and subduction of the Indian continental plate with the Asian plate during Himalayan orogeny (Shanker *et al*., 1976; Shanker, 1988; Harinarayana *et al*., 2006). These molten rocks are the ultimate source of the high heat-flow (540 mW m^-2^) and geothermal activity (manifested as gentle to vigorous hot springs having surface temperatures 45 to 85°C, sulfur condensates, borax deposits, mud pools and fumaroles) in the region (Shanker *et al*., 1976; Harinarayana *et al*., 2006).

**References used in Supplementary Note 1**

Azeez KKA, and Harinarayana T. Magnetotelluric evidence of potential geothermal resource in Puga, Ladakh, NW Himalaya. Curr Sci. 2007;93:323-329.

Chowdhury AN, Bose BB, Pal J, Yudhisthir, Sengupta NR. Studies of some minor and rare elements in hot spring deposit from Puga, Ladakh. Geol Surv IndiaSpec Publ. 1984;12:585–591.

Chowdhury AN, Handa BK, Das AK. High lithium, rubidium and cesium contents of thermal spring water, spring sediments and borax deposits in Puga valley, Kashmir, India. Geochem J. 1974;8:61-65.

Gansser A. Geology of the Himalayas. Interscience: London. 1964.

Harinarayana T, Azeez KKA, Murthy DN, Veeraswamy K, Rao SPE, Manoj C, et al. Exploration of geothermal structure in Puga geothermal field, Ladakh Himalayas, India by magnetotelluric studies. J Appl Geophys. 2006;58:280–295.

Saxena VK, and D’Amore F. Aquifer chemistry of the Puga and Chumathang high temperature geothermal systems in India. J Volcanol Geotherm. 1984;21:333–346.

Shanker R. Heat flow map of India and discussions on its geological and economic significance. Indian Miner. 1988;42:89-110.

Shanker R, Padhi RN, Arora CL, Prakash G, Thussu JL, Dua KJS. Geothermal exploration of the Puga and Chhumathang geothermal fields, Ladakh, India. In: Proceedings of the 2^nd^ United Nations symposium on the development and use of geothermal resources; May 20–29, 1975; San Francisco, CA. Washington, DC: U.S. Energy Research and Development Administration. 1976.

Shanker R, Absar A, Srivastava GC, Pandey SN. Source and significance of anomalously high cesium in geothermal fluid at Puga, Ladakh, India. In Proceedings of the 21st New Zealand Geothermal Workshop. 1999;21:79-82.

**Supplementary Note 2**

**Phylogenomic diversity analysis for the individual *Shivlinga* mats corroborated the trends of community dynamics along the hydrothermal gradients**

The duplicate metagenomic readsets of each community were co-assembled using GS *De Novo* Assembler (http://www.454.com) with all default parameters excepting that the minimum contig size was 1000 nucleotide. These contigs were classified down to phylum level using the software package Phyla-Amphora (Wang *et al*., 2013): all the protein coding sequences present in the contigs were identified, and then the conserved marker gene sequences were taxonomically annotated by searching against the custom database obtained from http://wolbachia.biology.virginia.edu/WuLab/Software.html. The numbers of contigs affiliated to phyla present in the mat communities were compared along the two thermal gradients.

Furthermore, the contigs obtained for each mat community was clustered into population genomes by differential coverage binning. For this purpose, the duplicate readsets were separately mapped onto the contigs using the BWA Software 0.7.10 (Dubrin, 2009); the resulting BAM files were sorted and indexed using Samtools 1.2 (Handsaker *et al*., 2009); eventually, the BAM files and the corresponding contigs were parsed into GroopM 0.3.0 (Imelfort *et al*., 2014) to generate an interactive groopm database. This led to the creation of core bins, which were then refined to eliminate putative chimeric bins from the groopm database. No further recruitment of unbinned contigs was made at this stage. Finally, the binned contigs were extracted, and evaluated using CheckM 0.7.0 (Parks *et al*., 2015) to determine the completeness, contamination percentage and heterogeneity for each bin. The numbers of population genome bins obtained from each mat community were compared along the two thermal gradients.

Diversity analyses based on contig assembly from the mat metagenomes were consistent with the trends revealed from direct classifications of the metagenomic reads (statistics of the contig assembly procedure are given in Table A). For VWM (66°C), the total number of taxonomically-classified contigs was far lower than in DG3 or WG3 (located at 52°C and 56°C, respectively). However, along the drying thermal gradient, the number of taxonomically-classified contigs in DG3 (52°C) was remarkably higher than in DG4 (41°C) (Table B). This loss of diversity may be attributable to colonization constraints imposed by dehydration. Again, the phylum-level break-up of the classifiable contigs obtained from each mat community of this gradient showed that, with the fall in temperature from 66°C to 52°C, contig count increased for almost all the phyla present. The sole exceptions were *Aquificae*, *Deinoccus-Thermus* and *Thermotogae* that continued to decrease until the end of the gradient (41°C) (Table B). Notably, contig counts were higherin DG3 (52°C) than in DG4 (41°C) for all phyla, except *Proteobacteria*. Along the wet thermal gradient, the total number of classified contigs in WG3 (56°C) was lower than that in WG4 (46°C) (Table B); below 46°C however, classified-contig count first decreased in WG5 (38°C), but subsequently increased in WG6 (36°C) and then increased further in WG7 (33°C). Phylum-level break-up of the classifiable contigs obtained from each mat community of this gradient showed that, with the drop in temperature from 66°C to 46°C (VWM to WG4), contig count increased progressively for all phyla including *Aquificae* and *Deinoccus-Thermus*. The sole exception was *Thermotogae*, for which contig count decreased between 66°C and 56°C, and then increased sharply again at 46°C. Increase in contig count with decreasing temperature was expected for the mesophile-dominated phyla, but for the thermophile-dominated *Aquificae*, *Deinoccus-Thermus* and *Thermotogae* this trend was unexpected. In this context it is noteworthy that when the contigs ascribed to *Aquificae*, *Deinoccus-Thermus* or *Thermotogae* in each mat community were clustered into population-genome bins (on the basis of differential coverage and GC content), bin count for *Aquificae* increased from three at the 66°C site (VWM) to five in the 46°C site (WG4), while those for *Thermotogae* increased from one in VWM to three in WG4. Below 46°C, however, *Aquificae* bin count went down to one each for WG5, WG6 and WG7; whereas for *Thermotogae*, bin count was zero, one and one for WG5, WG6 and WG7, respectively. For any mat community, the contigs ascribed to *Deinoccus-Thermus* did not cluster into population-genome bins having acceptable quality parameters (see Methods). These data indicate that only moderately thermophilic species of *Aquificae* and *Thermotogae* colonize the 46°C site of the wet thermal gradient. Furthermore, as temperature dropped below 46°C, along the wet thermal gradient, contig counts for the phyla present, generally decreased in WG5 (38°C), and then increased sharply in WG6 and WG7 (36°C and 33°C, respectively). The sole exception was *Verrucomicrobia* for which contig count increased throughout the gradient (Table B). It appears, therefore, that parameters other than temperature act as key determinants of community composition at sample sites below 46°C.

The number of population-genome bins, a direct function of the total phylogenomic diversity present, for the 66°C sitewas only 13 as compared to 120 and 85 for the 52°C (DG3) and 56°C sites (WG3), respectively (Table A). This underscored the presence of a thermodynamic hurdle for bacterial colonization at ~60°C. Along the wet thermal gradient there was again a small increase in bin count (from 85 to 92) commensurate with the fall in temperature from 56°C to 46°C. For the drying thermal gradient, however, bin count decreased (from 120 to 94) with the fall in temperature from 52°C to 41°C (Table B). This again illustrates that dehydration can constrain colonization along the drying thermal gradient. Along the wet thermal gradient, bin count dropped at 38°C, rose sharply at 36°C, and then dropped slightly at 33°C; so bin count did not correlate with temperature below 46°C.

**References used in Supplementary Note 2**

Imelfort M, Parks D, Woodcroft BJ, Dennis P, Hugenholtz P, Tyson GW. GroopM: an automated tool for the recovery of population genomes from related metagenomes. PeerJ. 2014;2:e603.

Li H, Durbin R. Fast and accurate short read alignment with Burrows-Wheeler transform. Bioinformatics. 2009;25:1754-1760.

Li H, Handsaker B, Wysoker A, Fennell T, Ruan J, Homer N, et al. Genome Project Data Processing S: The Sequence Alignment/Map format and SAMtools. Bioinformatics. 2009;25:2078-2079.

Parks DH, Imelfort M, Skennerton CT, Hugenholtz P, Tyson GW. CheckM: assessing the quality of microbial genomes recovered from isolates, single cells, and metagenomes. Genome Res. 2015;25:1043-1055.

Wang Z, Wu M. A phylum-level bacterial phylogenetic marker database. Mol Biol Evol. 2013;30:1258-1262.

**Table A.** Results of co-assembly of the duplicate metagenomic readsets of every mat community, and differential-coverage-based binning of the obtained contigs.

| **Sample** | **No. of**  **reads**  **used in assembly^1^** | **No. of**  **bases**  **used in assembly^1^** | **No. of reads participating in assembly** | **Size of the assembled sequence** | **No. of >1000-base pair (bp) contigs** | **No. of >100 Kb contigs** | **Size of largest contig (in bp)** | **Mean contig size**  **(in bp)** | **No. of contigs taking part in bin formation** | **No. of bins formed** |
| --- | --- | --- | --- | --- | --- | --- | --- | --- | --- | --- |
| **VWM** | 11,254,012 | 1,908,849,578 | 5,266,783 | 71,063,234 | 62,227 | 0 | 65,333 | 1,142 | 41,793 | 13 |
| **DG3** | 9,648,653 | 1,694,499,349 | 5,396,127 | 75,243,783 | 34,334 | 4 | 129,117 | 2,192 | 32,704 | 120 |
| **DG4** | 8,715,943 | 1,556,519,247 | 5,116,823 | 60,252,725 | 28,641 | 7 | 198,928 | 2,104 | 26,057 | 94 |
| **WG3** | 2,521,391 | 492,364,192 | 1,309,310 | 39,642,539 | 16,979 | 0 | 54,144 | 2,335 | 14,142 | 85 |
| **WG4** | 24,014,870 | 3,633,127,285 | 22,182,958 | 125,303,784 | 47,133 | 9 | 143,403 | 2,659 | 41,981 | 92 |
| **WG5** | 2,975,340 | 592,395,606 | 1,658,938 | 35,814,819 | 17,369 | 0 | 46,712 | 2,062 | 15,225 | 81 |
| **WG6** | 17,193,650 | 2,619,830,340 | 16,024,268 | 82,086,031 | 30,263 | 2 | 164,113 | 2,713 | 28,629 | 169 |
| **WG7** | 17,811,683 | 2,932,487,022 | 10,208,241 | 215,172,945 | 119,905 | 19 | 278,584 | 1,795 | 117,027 | 158 |

^1^ After removing <30 bp reads.

**Table B.** Phylum-level classification^1^ of the >1000-bp contigs obtained from co-assembly of the duplicate metagenomic readsets of every mat community.

|  | **VWM** | **DG3** | **DG4** | **VWM** | **WG3** | **WG4** | **WG5** | **WG6** | **WG7** |
| --- | --- | --- | --- | --- | --- | --- | --- | --- | --- |
| **Classification of the >1000-bp contigs** | | | | | | | | | |
| **Number of >1000-bp contigs obtained from co-assembly** | 62,227 | 34,334 | 28,641 | 62,227 | 16,979 | 47,133 | 17,369 | 30,263 | 119,905 |
| **Total number of classified contigs** | 2371 | 6802 | 4805 | 2371 | 5105 | 12133 | 3558 | 5208 | 16826 |
| *Bacteroidetes* contigs | 66 | 728 | 534 | 66 | 234 | 1072 | 134 | 1122 | 3505 |
| *Proteobacteria* contigs | 109 | 249 | 558 | 109 | 382 | 1070 | 62 | 948 | 2889 |
| *Cyanobacteria* contigs | 38 | 1661 | 726 | 38 | 652 | 1179 | 668 | 565 | 2494 |
| *Chlorobi* contigs | 194 | 1647 | 1410 | 194 | 1068 | 1490 | 1393 | 1024 | 2233 |
| *Chloroflexi* contigs | 46 | 1524 | 1323 | 46 | 916 | 1773 | 915 | 971 | 1908 |
| *Acidobacteria* contigs | 9 | 30 | 19 | 9 | 22 | 78 | 12 | 23 | 1073 |
| *Planctomycetes* contigs | 7 | 73 | 29 | 7 | 18 | 110 | 30 | 129 | 828 |
| *Spirochaetes* contigs | 3 | 257 | 71 | 3 | 5 | 358 | 186 | 17 | 609 |
| *Firmicutes* contigs | 65 | 118 | 45 | 65 | 88 | 706 | 33 | 113 | 519 |
| *Verrucomicrobia* contigs | 0 | 99 | 7 | 0 | 9 | 23 | 41 | 123 | 214 |
| *Deinococcus-Thermus* contigs | 372 | 281 | 41 | 372 | 728 | 700 | 30 | 67 | 167 |
| *Aquificae* contigs | 705 | 44 | 9 | 705 | 921 | 1981 | 11 | 29 | 97 |
| *Chlamydiae* contigs | 3 | 25 | 3 | 3 | 7 | 43 | 11 | 25 | 75 |
| *Thermotogae* contigs | 746 | 18 | 10 | 746 | 30 | 1399 | 10 | 14 | 75 |
| *Actinobacteria* contigs | 3 | 39 | 14 | 3 | 13 | 49 | 18 | 24 | 61 |
| *Fibrobacteres* contigs | 4 | 4 | 0 | 4 | 3 | 32 | 2 | 7 | 44 |
| *Fusobacteria* contigs | 1 | 5 | 5 | 1 | 9 | 59 | 2 | 6 | 32 |
| *Tenericutes* contigs | 0 | 0 | 1 | 0 | 0 | 11 | 0 | 1 | 3 |

^1^ Contigs were classified on the basis of the annotation of the conserved protein-coding marker genes present within them.

**Supplementary Note 3**

**The R script used to draw the heat map depicting the results of hierarchical cluster analysis**

library(gplots)

library("RColorBrewer")

aa<- read.csv("input.csv", sep=",")

row.names(aa) <- aa$Name

aa<- aa[,2:21]

aa_matrix <- data.matrix(aa)

hc.rows <- hclust(dist(aa_matrix))

plot(hc.rows)

hc.cols <- hclust(dist(t(aa_matrix)))

heatmap.2(as.matrix(aa_matrix), scale="none",key=TRUE, col=brewer.pal(9,"YlGn"), symkey=FALSE, density.info="none", trace="none", cexRow=0.5, lhei = c(2, 8))

**Supplementary Figures**


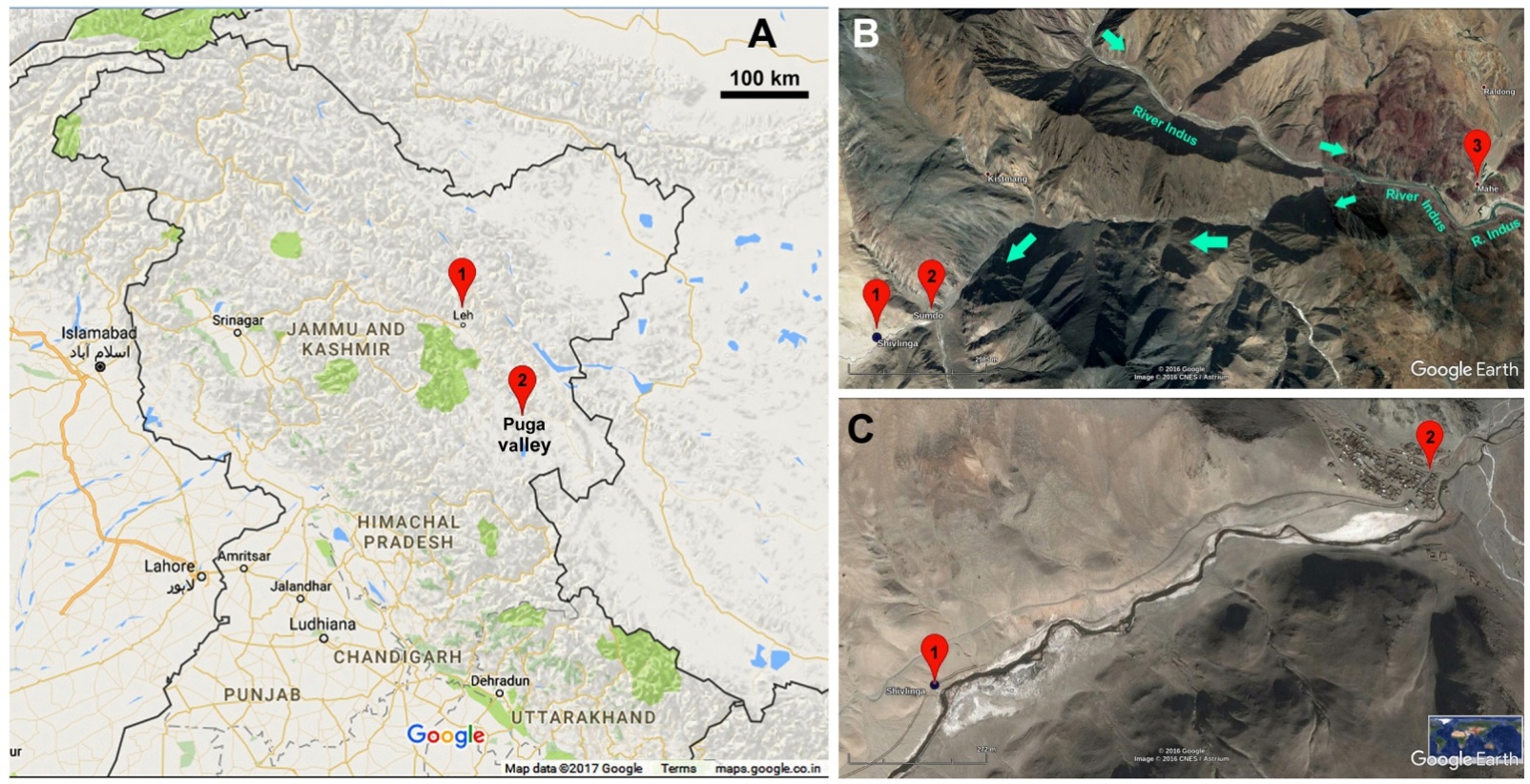


**Supplementary Fig. 1. Location of Puga Valley in eastern Ladakh.** (**A**) map showing the location of Leh, the capital of the union territory of Ladakh (red shape numbered as 1), and Puga Valley (red shape numbered as 2); (**B**) satellite image showing the position of *Shivlinga* (red shape numbered as 1) at the eastern extremity of Puga Valley, in context with the nearest village Sumdo (red shape numbered as 2) and the road that leads to Puga Valley from Leh (indicated by green arrows), running all along the Indus river and veering south across the river just before the village of Mahe (red shape numbered as 3); (**C**) zoomed-in satellite view of the eastern-extremity of Puga Valley showing the *Shivlinga* site and the Sumdo village. A, B and C, were sourced from the publicly available databases http://maps.google.com/ and http://earth.google.com/, respectively.


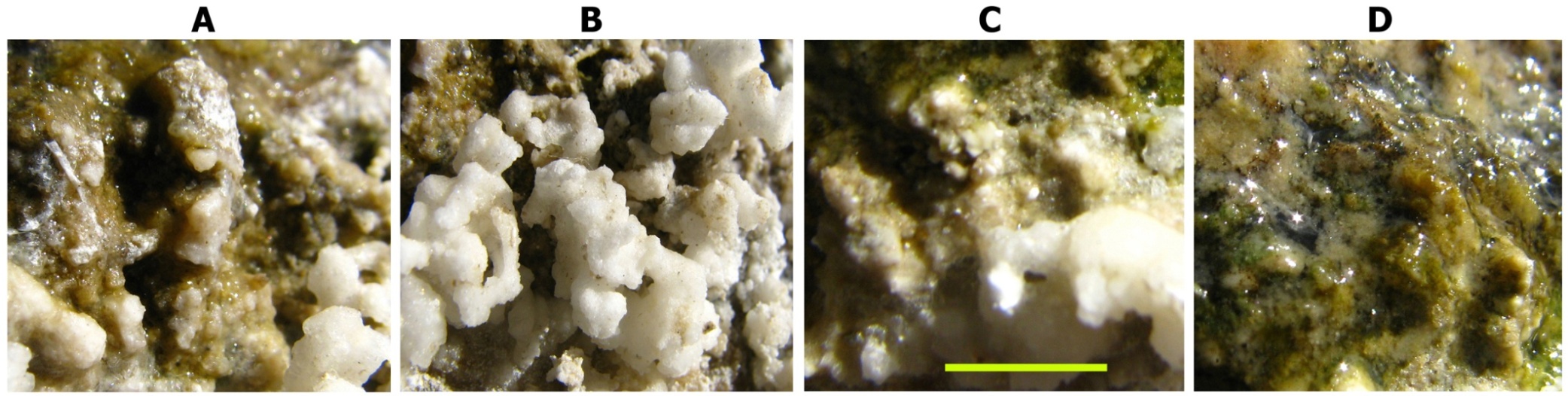


**Supplementary Fig. 2.** Photographic monitoring of the mineralization process over a mat portion situated at the margin of the spring-water flow, on the upper surface of the vent’s rim: (**A**) picture taken on Day 1, thin layers of white mineral covering the green mat; (**B**) picture taken on Day 7, soft white spherules and shrub-like bodies of accreted minerals cover the mat (minute green bodies can be seen protruding out of the spherules); (**C**), picture taken on Day 14, further growth of the green mat over the white mineral deposits; (**D**), picture taken on Day 21, the green mat grows completely out of the mineral covering. The green scale bar (= 10 mm) shown in panel C applies to all four panels.


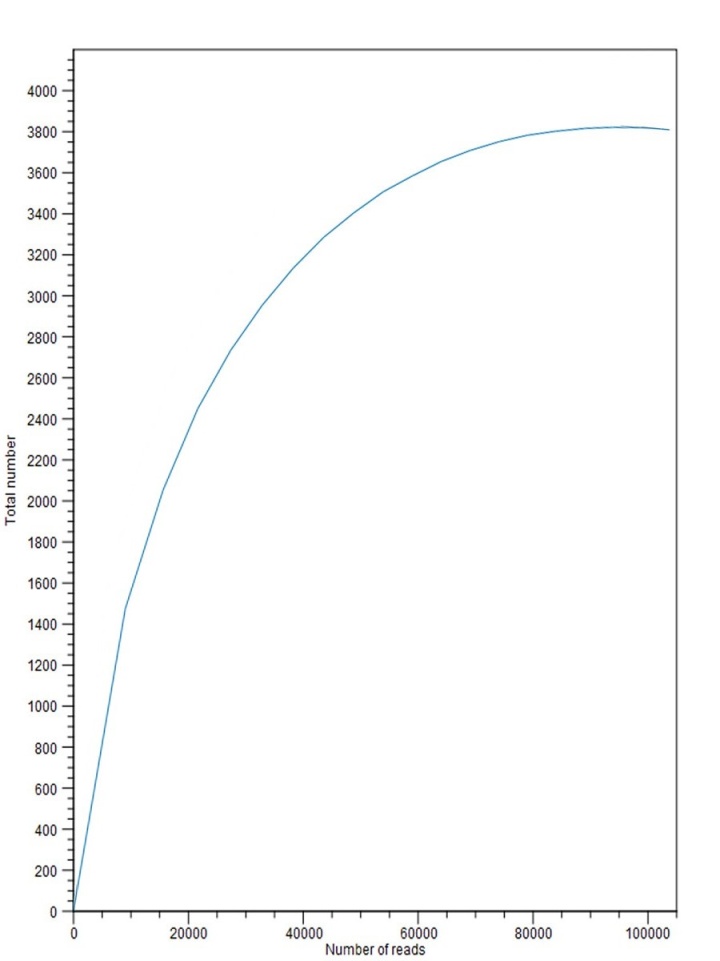


**Supplementary Fig. 3.** Rarefaction curve showing the proportionality between OTU-level metataxonomic diversity revealed and the number of amplified 16S rRNA gene sequence (V3 region) reads analyzed for the sample VW. Number of reads used in OTU building are plotted along the X-axis, while number of OTUs (including singletons) created at the 97% sequence identity level are plotted along the Y-axis.


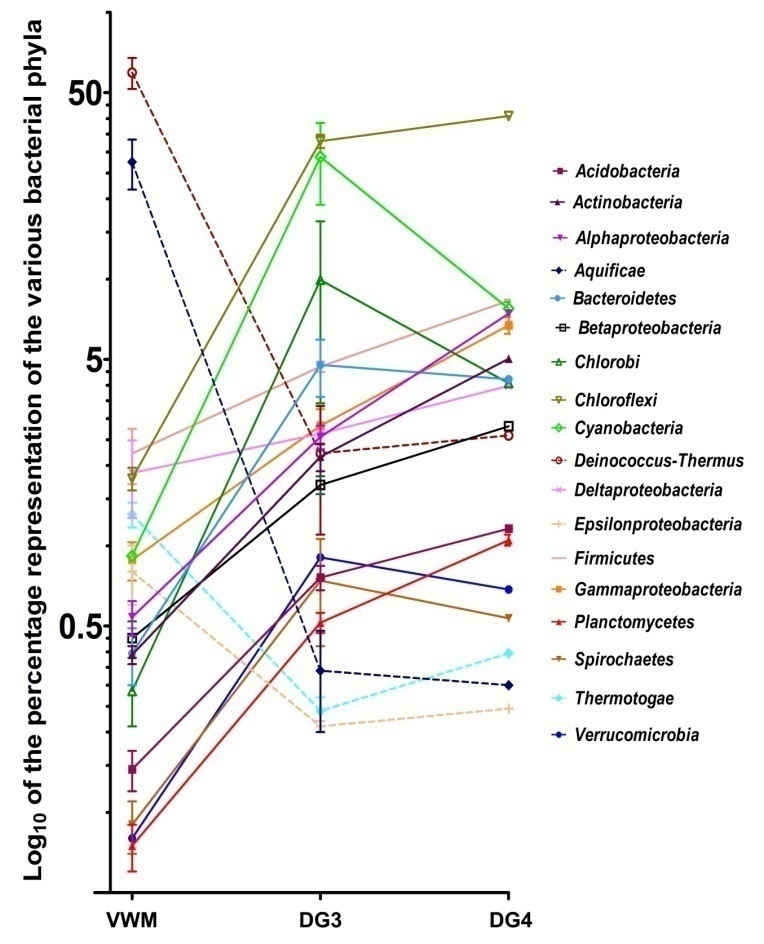


**Supplementary Fig. 4. Fluctuations in the relative abundance of bacterial phyla**/**proteobacterial classes along the drying thermal gradient.** For each phylum/class within a community, its mean relative abundance has been plotted alongside the two original relative abundance values obtained from the duplicate metagenomes (shown as vertical range bar). To compare the widely unequal relative abundance values comprehensively, their log to the base 10 are plotted along the Y-axis. Only those taxa which had > 1% read share in at least one of the six metagenomic readsets analyzed are shown.


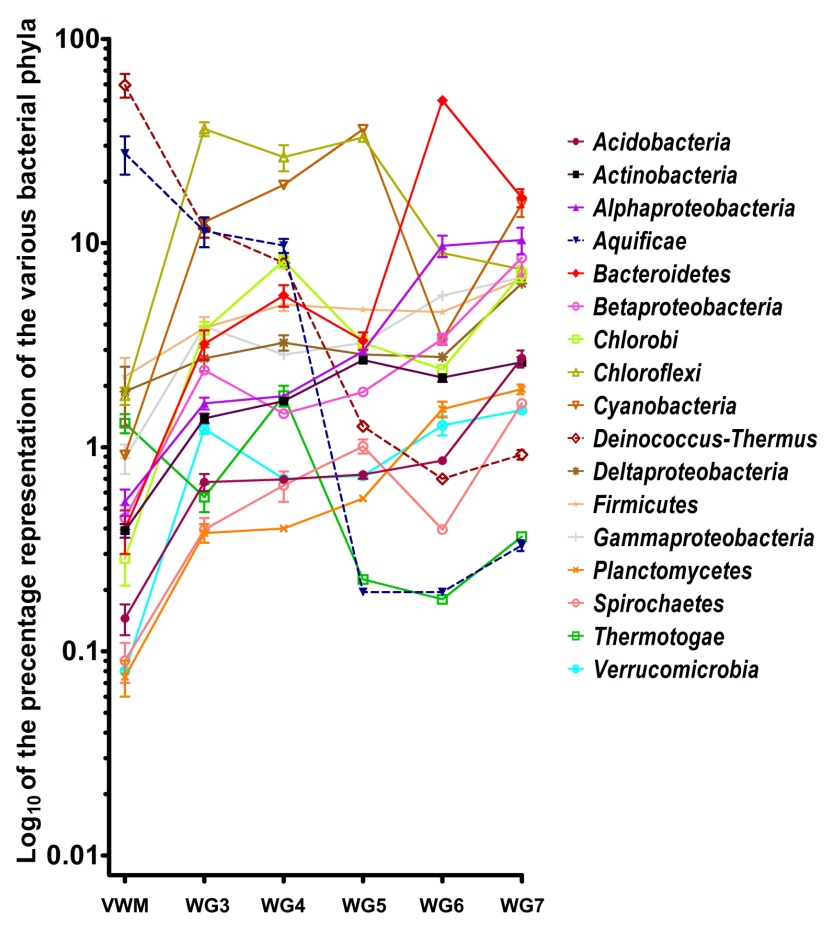


**Supplementary Fig. 5. Fluctuations in the relative abundance of bacterial phyla**/**proteobacterial classes along the wet thermal gradient.** For each phylum/class within a community, its mean relative abundance has been plotted alongside the two original relative abundance values obtained from the duplicate metagenomes (shown as vertical range bar). In order to compare the widely unequal relative abundance values comprehensively, their log to the base 10 are plotted along the Y-axis. Only those taxa which had > 1% read share in at least one of the twelve metagenomic readsets analyzed are shown.

**Supplementary Tables**

**Supplementary Table 1.** Maximum and minimum temperature, pH, and flow-rate recorded at the *Shivlinga* sample-sites during explorations prior to 23 July 2013 (previous dates of measurement are given in parenthesis after each value).

| **Name of the Sample** | **Maximum and minimum temperatures (in °C)** | **Maximum and minimum moisture (in % w/w)^*^** | **Maximum and minimum**  **flow rate (in cm s^-1^)^#^** | **Maximum and minimum**  **pH of the spring-water** |
| --- | --- | --- | --- | --- |
| **VW** | 73 (23.08.2008)  and  68 (12.08.2010) | NA | 26.5 (12.08.2010)  and  24.0 (23.08.2008) | 7.2 (12.08.2010)  and  6.8 (23.08.2008) |
| **VWM** | 69 (23.08.2008)  and  64 (12.08.2010) | NA | 26.5 (12.08.2010)  and  24.0 (23.08.2008) | 7.2 (12.08.2010)  and  6.8 (23.08.2008) |
| **DG3** | 54 (23.08.2008)  and  50 (12.08.2010) | 42.5 (12.08.2010)  and  40.0 (23.08.2008) | NA | 7.4 (12.08.2010)  and  7.1 (23.08.2008) |
| **DG4** | 44 (23.08.2008)  and  39 (12.08.2010) | 30.0 (12.08.2010)  and  27.2 (23.08.2008) | NA | 7.6 (12.08.2010)  and  7.2 (23.08.2008) |
| **WG3** | 58 (23.08.2008)  and  53 (12.08.2010) | NA | 23.0 (12.08.2010)  and  20.0 (23.08.2008) | 7.6 (12.08.2010)  and  7.3 (23.08.2008) |
| **WG4** | 48(23.08.2008)  and  45 (12.08.2010) | NA | 16.0 (12.08.2010)  and  13.0 (23.08.2008) | 8.0 (12.08.2010)  and  7.8 (23.08.2008) |
| **WG5** | 40 (23.08.2008)  and  37 (12.08.2010) | NA | 12.0 (12.08.2010)  and  9.5 (23.08.2008) | 8.3 (12.08.2010)  and  8.0 (23.08.2008) |
| **WG6** | 37 (23.08.2008)  and  34 (12.08.2010) | NA | 8.0 (12.08.2010)  and  6.0 (23.08.2008) | 8.4 (12.08.2010)  and  8.0 (23.08.2008) |
| **WG7** | 34 (23.08.2008)  and  31 (12.08.2010) | NA | 5.0 (12.08.2010)  and  4.5 (23.08.2008) | 8.5 (12.08.2010)  and  8.2 (23.08.2008) |

* Measuring moisture content was applicable only to the samples DG3 and DG4 of the progressively-drying thermal gradient as these mats grew on moist sinter surfaces off the outflow channel.Flow rates were measured for all the other communities as they grew directly on the hot water outflow.

^#^ Flow rate of water only at the vent (i.e. at sample sites of VW and VWM) was measured in ml s^-1^.

**Supplementary Table 2.** Elemental composition (amount measured as percent of total on a w/w basis*) of sinters deposited on the top, sides, interior, base, and apron, of *Shivlinga*.

| **Chemical element** | **Sinter sample 1 from one top-edge of *Shivlinga*’s body^1^** | **Sinter sample 2 from one side-surface of *Shivlinga*’s body^1^** | **Sinter sample 3 from the bedrock slope around *Shivlinga*’s base^1^** | **Sinter sample 4 from *Shivlinga*’s apron^1^** | **Sinter sample 5 from 5cm-deep within *Shivlinga*’s side^2^** |
| --- | --- | --- | --- | --- | --- |
| B | 26.23 | 25.22 | 22.85 | 21.08 | 20.04 |
| O | 29.76 | 30 | 31.89 | 32.75 | 28.33 |
| C | 13.62 | 10.2 | 5.47 | 12 | 8.8 |
| Na | 10.1 | 12.7 | 11.82 | 9.69 | 9.37 |
| Si | 1.26 | 1.37 | 0.73 | 1.52 | 3.2 |
| K | 3.04 | 2.56 | 0.9 | 0.31 | 2.39 |
| Ca | 4.07 | 3.98 | 3.6 | 3.22 | 3.28 |
| Cl | 0.58 | 0.63 | 0.46 | 0.29 | 0.43 |
| Al | 5.59 | 6.88 | 12.07 | 2.24 | 14.53 |
| Au | 5.1 | 5.74 | 5.08 | 0.82 | 6.6 |
| S | 0.07 | 0.18 | 1.7 | 0.15 | 3.18 |
| Fe | 0.08 | 0.07 | 0.7 | 3.56 | 0.09 |
| Mn | 0.13 | 0.25 | 0.53 | 1.53 | 0.16 |
| Zn | 0.09 | 0.15 | 0.46 | 1.74 | 0.2 |
| Mo | 0.79 | 0.61 | 0.84 | 1.29 | 0.36 |
| Pb | 0.62 | 0.57 | 0.77 | 1.68 | 0.44 |
| Ag | 0.43 | 0.33 | 0.66 | 3.27 | 0.25 |
| Ni | 0.31 | 0.24 | 0.23 | 1.75 | 0.1 |
| Co | 0.29 | 0.27 | 0.42 | 1.9 | 0.13 |

* Given values are means of five replicate analyses each by EDS and EPMA; hence totals may not add up to exactly 100%; standard deviations were <10% of the means.

^1^ All these four samples were recently-formed sinters occurring as mm- to cm-sized white amorphous spherules and shrubs.

^2^ Only this sample involved relatively older and harder sinters.

**Supplementary Table 3.** Summary statistics of metataxonomic analysis^1^ of the Shivlinga vent-water community (VW).

| **BioSample Accession Number** | SAMN04241741 |
| --- | --- |
| **Run Accession Number** | SRR2904995 |
| **Total reads** | 243,609 |
| **Reads after Quality & Length filtering** | 199,783 |
| **Reads after Dereplication** | 20,987 |
| **Total OTUs (minus singletons)** | 64 |
| **Singletons Only** | 6,890 |
| **ACE estimation (minus singletons)** | 64 |
| **Shannon index (minus singletons)** | 1.47278 |
| **Simpson index (minus singletons)** | 0.886 |

^1^ V3 regions of all bacterial 16S rRNA genes present in the total environmental DNA isolated from the VW sample was amplified and sequenced using Ion PGM. OTUs were clustered at the 97% sequence similarity level.

**Supplementary Table 4.** Summary of metagenomic investigation of the mat communities of the progressively-drying thermal gradient.

|  | **VWM** | | **DG3** | | **DG4** | |
| --- | --- | --- | --- | --- | --- | --- |
| **Sampling** | **1^st^ sample** | **2^nd^ sample** | **1^st^ sample** | **2^nd^ sample** | **1^st^ sample** | **2^nd^ sample** |
| **Run**  **accession no.** | SRR2625861 | SRR2626156 | SRR2625863 | SRR2626157 | SRR2625864 | SRR2626159 |
| **Data volume**  **(no. of mega bases)** | 362 | 1,240 | 400 | 1,294 | 475 | 1,082 |
| **Read**  **throughput** | 1,875,410 | 7,784,680 | 1,902,386 | 7,746,267 | 2,233,295 | 6,482,648 |
| **Mean read-length (no. of bases)** | 193 | 159 | 210 | 167 | 213 | 167 |
| **No, of reads finally annotated after quality filtering^1^** | 955,353 | 6,644,298 | 808,927 | 4,903,766 | 1,060,639 | 3,647,816 |
| **No. of reads ascribed to *Archaea*^1^** | 4,340  (0.45 %) | 50,772  (0.76 %) | 4,470  (0.55 %) | 27,519  (0.56 %) | 10,078  (0.95 %) | 34,532  (0.95 %) |
| **No. of reads ascribed to *Bacteria*^1^** | 874,680  (91.56 %) | 5,847,280  (88 %) | 594859  (73.54 %) | 3642131  (74.27 %) | 795944  (75.04 %) | 2724833  (74.7 %) |
| **No. of reads ascribed to *Eukarya*^1^** | 2,263  (0.24 %) | 14,628  (0.22 %) | 4927  (0.61 %) | 48950  (1 %) | 9095  (0.86 %) | 31302  (0.86 %) |
| **No. of reads ascribed to Viruses^1^** | 2,823  (0.3 %) | 11,593  (0.17 %) | 64  (0.01 %) | 1072  (0.02 %) | 116  (0.01 %) | 471  (0.01 %) |
| **No. of unannotable reads^1^** | 71,247  (7.46 %) | 720,025  (10.84 %) | 204607  (25.29 %) | 1184094  (24.15 %) | 245406  (23.14 %) | 856678  (23.48 %) |

^1^ As determined using the Organism Abundance tool of MG-RAST. Reads were searched by BlastX against the *nr* protein database following the Best Hit Classification approach with minimum alignment length of 15 amino acids and minimum identity cutoff of 60%. Values in parenthesis express read counts as percentages of the total number of annotable reads.

**Supplementary Table 5.** Summary of metagenomic investigation of the mat communities^1^ of the wet thermal gradient.

|  | **WG3** | | **WG4** | | **WG5** | | **WG6** | | **WG7** | |
| --- | --- | --- | --- | --- | --- | --- | --- | --- | --- | --- |
| **Sampling** | **1^st^ sample** | **2^nd^ sample** | **1^st^ sample** | **2^nd^ sample** | **1^st^ sample** | **2^nd^ sample** | **1^st^ sample** | **2^nd^ sample** | **1^st^ sample** | **2^nd^ sample** |
| **Run**  **accession no.** | SRR2625866 | SRR2626428 | SRR2625865 | SRR2626160 | SRR2625867 | SRR2626164 | SRR2626012 | SRR2626185 | SRR2626152 | SRR2626309 |
| **Data volume**  **(no. of mega bases)** | 253 | 240 | 1,721 | 1,912 | 318 | 274 | 1,486 | 1,134 | 1,700 | 1,232 |
| **Read**  **throughput** | 1,484,481 | 1,036,910 | 10,202,786 | 13,812,084 | 1,785,852 | 1,189,488 | 9,055,702 | 8,137,948 | 10,301,770 | 7,509,913 |
| **Mean read-length (no. of bases)** | 170 | 231 | 169 | 138 | 178 | 231 | 164 | 139 | 165 | 164 |
| **No, of reads finally annotated after quality filtering^2^** | 967,344 | 1,028,280 | 5,465,737 | 9,061,326 | 867,988 | 984,610 | 3,151,051 | 4,043,750 | 3,876,194 | 4,062,948 |
| **No. of reads ascribed to *Archaea*^2^** | 5380  (0.56 %) | 8051  (0.78 %) | 39779  (0.73 %) | 81826  (0.9 %) | 5144  (0.59 %) | 5991  (0.61 %) | 14455  (0.46 %) | 19263  (0.48 %) | 30515  (0.79 %) | 33729  (0.83 %) |
| **No. of reads ascribed to *Bacteria*^2^** | 700263  (72.39 %) | 767239  (74.61 %) | 4147079  (75.87 %) | 7076793  (78.1 %) | 644915  (74.3 %) | 738345  (74.99 %) | 2432437  (77.19 %) | 3113661  (77 %) | 3139039  (80.98 %) | 3296247  (81.13 %) |
| **No. of reads ascribed to *Eukarya*^2^** | 3798  (0.39 %) | 4583  (0.45 %) | 30495  (0.56 %) | 56683  (0.63 %) | 8825  (1.02 %) | 9152  (0.93 %) | 29725  (0.94 %) | 38760  (0.96 %) | 42558  (1.1 %) | 47061  (1.16 %) |
| **No. of reads ascribed to Viruses^2^** | 304  (0.03 %) | 337  (0.03 %) | 874  (0.02 %) | 1691  (0.02 %) | 153  (0.02 %) | 259  (0.03 %) | 779  (0.02 %) | 1430  (0.04 %) | 651  (0.02 %) | 978  (0.02 %) |
| **No. of unannotable reads^2^** | 257599  (26.63 %) | 248070  (24.12 %) | 1247510  (22.82 %) | 1844333  (20.35 %) | 208951  (24.07 %) | 230863  (23.45 %) | 673655  (21.38 %) | 870636  (21.53 %) | 663431  (17.12 %) | 684933  (16.86 %) |

^1^ VWM has been excluded from this table to avoid repetition of data already given in Table S1. Notably, however, VWM was also a part of the community continuum along the wet thermal gradient.

^2^ As determined using the Organism Abundance tool of MG-RAST. Reads were searched by BlastX against the *nr* protein database following the Best Hit Classification approach with minimum alignment length of 15 amino acids and minimum identity cutoff of 60%. Values in parenthesis express read counts as percentages of the total number of annotable reads.

**Supplementary Table 6.** Significant pair-wise Pearson correlation coefficients (CC) [and their corresponding Benjamini-Hochberg-corrected probability (*P*) values]* determined between phylum-/class-level population-fluctuations along the wet thermal gradient and variations in temperature, pH, distance from the vent, and flow-rate of the spring-water along the same trajectory.

|  | **Temperature** | | **pH** | | **Distance** | | **Flow rate** | |
| --- | --- | --- | --- | --- | --- | --- | --- | --- |
|  | **CC** | ***P*** | **CC** | ***P*** | **CC** | ***P*** | **CC** | ***P*** |
| ***Deinococcus-Thermus*** | 0.875 | 0.022 | -0.872 | 0.024 | -0.621 | 0.188 | 0.787 | 0.063 |
| ***Aquificae*** | 0.961 | 0.002 | -0.944 | 0.005 | -0.767 | 0.075 | 0.904 | 0.013 |
| ***Firmicutes*** | -0.883 | 0.020 | 0.914 | 0.011 | 0.844 | 0.035 | -0.863 | 0.027 |
| ***Deltaproteobacteria*** | -0.646 | 0.166 | 0.687 | 0.132 | 0.828 | 0.042 | -0.680 | 0.137 |
| ***Chloroflexi*** | -0.034 | 0.949 | 0.021 | 0.968 | -0.357 | 0.488 | 0.150 | 0.777 |
| ***Thermotogae*** | 0.571 | 0.237 | -0.506 | 0.306 | -0.581 | 0.227 | 0.557 | 0.251 |
| ***Cyanobacteria*** | -0.447 | 0.374 | 0.437 | 0.386 | 0.123 | 0.816 | -0.306 | 0.556 |
| ***Gammaproteobacteria*** | -0.812 | 0.050 | 0.814 | 0.049 | 0.886 | 0.019 | -0.830 | 0.041 |
| ***Epsilonproteobacteria*** | 0.816 | 0.047 | -0.783 | 0.066 | -0.533 | 0.276 | 0.709 | 0.114 |
| ***Alphaproteobacteria*** | -0.795 | 0.059 | 0.791 | 0.061 | 0.944 | 0.005 | -0.874 | 0.023 |
| ***Betaproteobacteria*** | -0.669 | 0.146 | 0.690 | 0.129 | 0.898 | 0.015 | -0.723 | 0.104 |
| ***Bacteroidetes*** | -0.566 | 0.242 | 0.544 | 0.265 | 0.585 | 0.222 | -0.640 | 0.171 |
| ***Actinobacteria*** | -0.963 | 0.002 | 0.950 | 0.004 | 0.790 | 0.061 | -0.908 | 0.012 |
| ***Nitrospirae*** | 0.354 | 0.492 | -0.274 | 0.599 | -0.378 | 0.460 | 0.351 | 0.495 |
| ***Chlorobi*** | -0.496 | 0.317 | 0.564 | 0.243 | 0.389 | 0.446 | -0.445 | 0.376 |
| ***Acidobacteria*** | -0.681 | 0.137 | 0.710 | 0.114 | 0.881 | 0.020 | -0.722 | 0.105 |
| ***Deferribacteres*** | 0.071 | 0.894 | 0.012 | 0.982 | -0.158 | 0.765 | 0.087 | 0.870 |
| ***Dictyoglomi*** | 0.199 | 0.706 | -0.123 | 0.816 | -0.334 | 0.517 | 0.227 | 0.665 |
| ***Spirochaetes*** | -0.751 | 0.085 | 0.773 | 0.071 | 0.795 | 0.059 | -0.735 | 0.096 |
| **Unclassified Bacteria** | -0.970 | 0.001 | 0.970 | 0.001 | 0.866 | 0.026 | -0.942 | 0.005 |
| ***Verrucomicrobia*** | -0.695 | 0.125 | 0.696 | 0.125 | 0.713 | 0.112 | -0.677 | 0.140 |
| ***Planctomycetes*** | -0.813 | 0.049 | 0.817 | 0.047 | 0.967 | 0.002 | -0.884 | 0.019 |
| ***Synergistetes*** | -0.615 | 0.194 | 0.671 | 0.144 | 0.703 | 0.119 | -0.617 | 0.192 |
| ***Fusobacteria*** | -0.764 | 0.077 | 0.802 | 0.055 | 0.846 | 0.034 | -0.811 | 0.050 |
| **Others** | -0.822 | 0.044 | 0.851 | 0.032 | 0.921 | 0.009 | -0.854 | 0.030 |
| **Unclassified *Proteobacteria*** | -0.817 | 0.047 | 0.819 | 0.046 | 0.902 | 0.014 | -0.824 | 0.044 |
| ***Tenericutes*** | -0.685 | 0.133 | 0.722 | 0.105 | 0.652 | 0.161 | -0.721 | 0.106 |
| ***Chlamydiae*** | -0.897 | 0.015 | 0.909 | 0.012 | 0.956 | 0.003 | -0.929 | 0.007 |
| ***Gemmatimonadetes*** | -0.795 | 0.059 | 0.825 | 0.043 | 0.878 | 0.021 | -0.804 | 0.054 |

* Positive and negative CC values are shaded light green and pink respectively; CC values not found to be significant according to Benjamini-Hochberg-corrected *P* values are not shown (gray cells).

**Supplementary Table 7.** Raw data used for determining the pair-wise Pearson correlation coefficients between phylum-/class-level population-fluctuations along the wet thermal gradient and variations in temperature, pH, distance from the vent, and flow-rate of the spring-water along the same trajectory.

|  | **VWM** | **WG3** | **WG4** | **WG5** | **WG6** | **WG7** |
| --- | --- | --- | --- | --- | --- | --- |
| **Temperature** | 66 | 56 | 46 | 38 | 36 | 33 |
| **pH** | 7.1 | 7.5 | 8 | 8.2 | 8.3 | 8.5 |
| **Distance** | 8 | 22 | 65 | 120 | 190 | 290 |
| **Flow rate** | 25 | 22 | 15 | 11 | 7 | 5 |
| ***Deinococcus-Thermus*** | 59.465 | 11.825 | 7.995 | 1.265 | 0.7 | 0.92 |
| ***Aquificae*** | 27.48 | 11.445 | 9.715 | 0.195 | 0.195 | 0.33 |
| ***Firmicutes*** | 2.22 | 3.84 | 4.99 | 4.73 | 4.59 | 6.665 |
| ***Deltaproteobacteria*** | 1.875 | 2.72 | 3.25 | 2.845 | 2.765 | 6.335 |
| ***Chloroflexi*** | 1.785 | 36.22 | 26.345 | 32.895 | 8.95 | 7.425 |
| ***Thermotogae*** | 1.31 | 0.57 | 1.79 | 0.225 | 0.18 | 0.365 |
| ***Cyanobacteria*** | 0.915 | 12.66 | 19.275 | 36.055 | 3.375 | 15.565 |
| ***Gammaproteobacteria*** | 0.885 | 3.905 | 2.84 | 3.25 | 5.53 | 6.78 |
| ***Epsilonproteobacteria*** | 0.74 | 0.465 | 0.495 | 0.2 | 0.4 | 0.385 |
| ***Alphaproteobacteria*** | 0.54 | 1.64 | 1.78 | 2.95 | 9.71 | 10.345 |
| ***Betaproteobacteria*** | 0.45 | 2.385 | 1.46 | 1.865 | 3.395 | 8.475 |
| ***Bacteroidetes*** | 0.395 | 3.205 | 5.545 | 3.325 | 49.98 | 16.68 |
| ***Actinobacteria*** | 0.39 | 1.385 | 1.685 | 2.67 | 2.195 | 2.61 |
| ***Nitrospirae*** | 0.29 | 0.205 | 0.475 | 0.13 | 0.12 | 0.22 |
| ***Chlorobi*** | 0.285 | 3.74 | 8.195 | 3.245 | 2.41 | 7.07 |
| ***Acidobacteria*** | 0.145 | 0.675 | 0.695 | 0.735 | 0.86 | 2.735 |
| ***Deferribacteres*** | 0.13 | 0.14 | 0.35 | 0.07 | 0.1 | 0.165 |
| ***Dictyoglomi*** | 0.11 | 0.085 | 0.285 | 0.06 | 0.045 | 0.085 |
| ***Spirochaetes*** | 0.09 | 0.395 | 0.65 | 1.01 | 0.395 | 1.64 |
| **Unclassified *Bacteria*** | 0.09 | 0.305 | 0.35 | 0.415 | 0.47 | 0.53 |
| ***Verrucomicrobia*** | 0.08 | 1.23 | 0.7 | 0.725 | 1.28 | 1.52 |
| ***Planctomycetes*** | 0.075 | 0.38 | 0.4 | 0.56 | 1.535 | 1.925 |
| ***Synergistetes*** | 0.06 | 0.11 | 0.155 | 0.105 | 0.095 | 0.225 |
| ***Fusobacteria*** | 0.06 | 0.1 | 0.14 | 0.085 | 0.16 | 0.195 |
| **Others** | 0.05 | 0.155 | 0.195 | 0.155 | 0.245 | 0.385 |
| **Unclassified *Proteobacteria*** | 0.04 | 0.055 | 0.055 | 0.09 | 0.07 | 0.125 |
| ***Tenericutes*** | 0.03 | 0.04 | 0.055 | 0.035 | 0.06 | 0.055 |
| ***Chlamydiae*** | 0.025 | 0.07 | 0.085 | 0.085 | 0.135 | 0.17 |
| ***Gemmatimonadetes*** | 0.01 | 0.07 | 0.08 | 0.07 | 0.085 | 0.17 |

**Supplementary Table 8.** Pearson correlation co-efficient (CC) values (*r*) and corresponding probability (*P*) values between phylum-/class-level population fluctuations in the mats of the wet thermal gradient and the variations in environmental parameters along the same trajectory.

|  | **Pearson CC values (*r*)** | | | | ***P*values obtained for individual *r* values** | | | |
| --- | --- | --- | --- | --- | --- | --- | --- | --- |
|  | **Temperature** | **pH** | **Distance** | **Flow rate** | **Temperature** | **pH** | **Distance** | **Flow rate** |
| ***Deinococcus-Thermus*** | 0.875 | -0.872 | -0.621 | 0.787 | 0.022 | 0.024 | 0.188 | 0.063 |
| ***Aquificae*** | 0.961 | -0.944 | -0.767 | 0.904 | 0.002 | 0.005 | 0.075 | 0.013 |
| ***Firmicutes*** | -0.883 | 0.914 | 0.844 | -0.863 | 0.020 | 0.011 | 0.035 | 0.027 |
| ***Deltaproteobacteria*** | -0.646 | 0.687 | 0.828 | -0.680 | 0.166 | 0.132 | 0.042 | 0.137 |
| ***Chloroflexi*** | -0.034 | 0.021 | -0.357 | 0.150 | 0.949 | 0.968 | 0.488 | 0.777 |
| ***Thermotogae*** | 0.571 | -0.506 | -0.581 | 0.557 | 0.237 | 0.306 | 0.227 | 0.251 |
| ***Cyanobacteria*** | -0.447 | 0.437 | 0.123 | -0.306 | 0.374 | 0.386 | 0.816 | 0.556 |
| ***Gammaproteobacteria*** | -0.812 | 0.814 | 0.886 | -0.830 | 0.050 | 0.049 | 0.019 | 0.041 |
| ***Epsilonproteobacteria*** | 0.816 | -0.783 | -0.533 | 0.709 | 0.047 | 0.066 | 0.276 | 0.114 |
| ***Alphaproteobacteria*** | -0.795 | 0.791 | 0.944 | -0.874 | 0.059 | 0.061 | 0.005 | 0.023 |
| ***Betaproteobacteria*** | -0.669 | 0.690 | 0.898 | -0.723 | 0.146 | 0.129 | 0.015 | 0.104 |
| ***Bacteroidetes*** | -0.566 | 0.544 | 0.585 | -0.640 | 0.242 | 0.265 | 0.222 | 0.171 |
| ***Actinobacteria*** | -0.963 | 0.950 | 0.790 | -0.908 | 0.002 | 0.004 | 0.061 | 0.012 |
| ***Nitrospirae*** | 0.354 | -0.274 | -0.378 | 0.351 | 0.492 | 0.599 | 0.460 | 0.495 |
| ***Chlorobi*** | -0.496 | 0.564 | 0.389 | -0.445 | 0.317 | 0.243 | 0.446 | 0.376 |
| ***Acidobacteria*** | -0.681 | 0.710 | 0.881 | -0.722 | 0.137 | 0.114 | 0.020 | 0.105 |
| ***Deferribacteres*** | 0.071 | 0.012 | -0.158 | 0.087 | 0.894 | 0.982 | 0.765 | 0.870 |
| ***Dictyoglomi*** | 0.199 | -0.123 | -0.334 | 0.227 | 0.706 | 0.816 | 0.517 | 0.665 |
| ***Spirochaetes*** | -0.751 | 0.773 | 0.795 | -0.735 | 0.085 | 0.071 | 0.059 | 0.096 |
| **Unclassified *Bacteria*** | -0.970 | 0.970 | 0.866 | -0.942 | 0.001 | 0.001 | 0.026 | 0.005 |
| ***Verrucomicrobia*** | -0.695 | 0.696 | 0.713 | -0.677 | 0.125 | 0.125 | 0.112 | 0.140 |
| ***Planctomycetes*** | -0.813 | 0.817 | 0.967 | -0.884 | 0.049 | 0.047 | 0.002 | 0.019 |
| ***Synergistetes*** | -0.615 | 0.671 | 0.703 | -0.617 | 0.194 | 0.144 | 0.119 | 0.192 |
| ***Fusobacteria*** | -0.764 | 0.802 | 0.846 | -0.811 | 0.077 | 0.055 | 0.034 | 0.050 |
| **Others** | -0.822 | 0.851 | 0.921 | -0.854 | 0.044 | 0.032 | 0.009 | 0.030 |
| **Unclassified *Proteobacteria*** | -0.817 | 0.819 | 0.902 | -0.824 | 0.047 | 0.046 | 0.014 | 0.044 |
| ***Tenericutes*** | -0.685 | 0.722 | 0.652 | -0.721 | 0.133 | 0.105 | 0.161 | 0.106 |
| ***Chlamydiae*** | -0.897 | 0.909 | 0.956 | -0.929 | 0.015 | 0.012 | 0.003 | 0.007 |
| ***Gemmatimonadetes*** | -0.795 | 0.825 | 0.878 | -0.804 | 0.059 | 0.043 | 0.021 | 0.054 |

**Supplementary Table 9.** Benjamini-Hochberg correction^1^ of the *P* values obtained for individual Pearson correlation co-efficient (CC) values (*r*) between the phylum-/class-level population fluctuations along the wet thermal gradient and the variations in temperature along the same trajectory.

|  | **CC value (*r*)** | ***P* value** | **Rank** | **Benjamini-Hochberg critical value** |
| --- | --- | --- | --- | --- |
| **Unclassified *Bacteria*** | -0.970 | 0.001 | 1 | 0.006896552 |
| ***Actinobacteria*** | -0.963 | 0.002 | 2 | 0.013793103 |
| ***Aquificae*** | 0.961 | 0.002 | 3 | 0.020689655 |
| ***Chlamydiae*** | -0.897 | 0.015 | 4 | 0.027586207 |
| ***Firmicutes*** | -0.883 | 0.020 | 5 | 0.034482759 |
| ***Deinococcus-Thermus*** | 0.875 | 0.022 | 6 | 0.04137931 |
| **Others** | -0.822 | 0.044 | 7 | 0.048275862 |
| **Unclassified *Proteobacteria*** | -0.817 | 0.047 | 8 | 0.055172414 |
| ***Epsilonproteobacteria*** | 0.816 | 0.047 | 9 | 0.062068966 |
| ***Planctomycetes*** | -0.813 | 0.049 | 10 | 0.068965517 |
| ***Gammaproteobacteria*** | -0.812 | 0.050 | 11 | 0.075862069 |
| ***Gemmatimonadetes*** | -0.795 | 0.059 | 12 | 0.082758621 |
| ***Alphaproteobacteria*** | -0.795 | 0.059 | 13 | 0.089655172 |
| ***Fusobacteria*** | -0.764 | 0.077 | 14 | 0.096551724 |
| ***Spirochaetes*** | -0.751 | 0.085 | 15 | 0.103448276 |
| ***Verrucomicrobia*** | -0.695 | 0.125 | 16 | 0.110344828 |
| ***Tenericutes*** | -0.685 | 0.133 | 17 | 0.117241379 |
| ***Acidobacteria*** | -0.681 | 0.137 | 18 | 0.124137931 |
| ***Betaproteobacteria*** | -0.669 | 0.146 | 19 | 0.131034483 |
| ***Deltaproteobacteria*** | -0.646 | 0.166 | 20 | 0.137931034 |
| ***Synergistetes*** | -0.615 | 0.194 | 21 | 0.144827586 |
| ***Thermotogae*** | 0.571 | 0.237 | 22 | 0.151724138 |
| ***Bacteroidetes*** | -0.566 | 0.242 | 23 | 0.15862069 |
| ***Chlorobi*** | -0.496 | 0.317 | 24 | 0.165517241 |
| ***Cyanobacteria*** | -0.447 | 0.374 | 25 | 0.172413793 |
| ***Nitrospirae*** | 0.354 | 0.492 | 26 | 0.179310345 |
| ***Dictyoglomi*** | 0.199 | 0.706 | 27 | 0.186206897 |
| ***Deferribacteres*** | 0.071 | 0.894 | 28 | 0.193103448 |
| ***Chloroflexi*** | -0.034 | 0.949 | 29 | 0.2 |

^1^ Phyla/classes were arranged in the ascending order of their *P* values (the one with the lowest *P* was ascribed rank 1); Benjamini-Hochberg critical values were determined as described in the Methods section; correlations were considered significant up to that phylum/class rank (see the phylum/class names that are in red font) where Benjamini-Hochberg critical value was still greater than the *P* value.

**Supplementary Table 10.** Benjamini-Hochberg correction^1^ of the *P* values obtained for individual Pearson correlation co-efficient (CC) values (*r*) between the phylum-/class-level population fluctuations along the wet thermal gradient and the variations in pH along the same trajectory.

|  | **CC value (*r*)** | ***P* value** | **Rank** | **Benjamini-Hochberg critical value** |
| --- | --- | --- | --- | --- |
| **Unclassified *Bacteria*** | 0.970 | 0.001 | 1 | 0.006896552 |
| ***Actinobacteria*** | 0.950 | 0.004 | 2 | 0.013793103 |
| ***Aquificae*** | -0.944 | 0.005 | 3 | 0.020689655 |
| ***Firmicutes*** | 0.914 | 0.011 | 4 | 0.027586207 |
| ***Chlamydiae*** | 0.909 | 0.012 | 5 | 0.034482759 |
| ***Deinococcus-Thermus*** | -0.872 | 0.024 | 6 | 0.04137931 |
| **Others** | 0.851 | 0.032 | 7 | 0.048275862 |
| ***Gemmatimonadetes*** | 0.825 | 0.043 | 8 | 0.055172414 |
| ***unclassified Proteobacteria*** | 0.819 | 0.046 | 9 | 0.062068966 |
| ***Planctomycetes*** | 0.817 | 0.047 | 10 | 0.068965517 |
| ***Gammaproteobacteria*** | 0.814 | 0.049 | 11 | 0.075862069 |
| ***Fusobacteria*** | 0.802 | 0.055 | 12 | 0.082758621 |
| ***Alphaproteobacteria*** | 0.791 | 0.061 | 13 | 0.089655172 |
| ***Epsilonproteobacteria*** | -0.783 | 0.066 | 14 | 0.096551724 |
| ***Spirochaetes*** | 0.773 | 0.071 | 15 | 0.103448276 |
| ***Tenericutes*** | 0.722 | 0.105 | 16 | 0.110344828 |
| ***Acidobacteria*** | 0.710 | 0.114 | 17 | 0.117241379 |
| ***Verrucomicrobia*** | 0.696 | 0.125 | 18 | 0.124137931 |
| ***Betaproteobacteria*** | 0.690 | 0.129 | 19 | 0.131034483 |
| ***Deltaproteobacteria*** | 0.687 | 0.132 | 20 | 0.137931034 |
| ***Synergistetes*** | 0.671 | 0.144 | 21 | 0.144827586 |
| ***Chlorobi*** | 0.564 | 0.243 | 22 | 0.151724138 |
| ***Bacteroidetes*** | 0.544 | 0.265 | 23 | 0.15862069 |
| ***Thermotogae*** | -0.506 | 0.306 | 24 | 0.165517241 |
| ***Cyanobacteria*** | 0.437 | 0.386 | 25 | 0.172413793 |
| ***Nitrospirae*** | -0.274 | 0.599 | 26 | 0.179310345 |
| ***Dictyoglomi*** | -0.123 | 0.816 | 27 | 0.186206897 |
| ***Chloroflexi*** | 0.021 | 0.968 | 28 | 0.193103448 |
| ***Deferribacteres*** | 0.012 | 0.982 | 29 | 0.2 |

^1^ Phyla/classes were arranged in the ascending order of their *P* values (the one with the lowest *P* was ascribed rank 1); Benjamini-Hochberg critical values were determined as described in the Methods section; correlations were considered significant up to that phylum/class rank (see the phylum/class names that are in red font) where Benjamini-Hochberg critical value was still greater than the *P* value.

**Supplementary Table 11.** Benjamini-Hochberg correction^1^ of the *P* values obtained for individual Pearson correlation co-efficient (CC) values (*r*) between the phylum-/class-level population fluctuations along the wet thermal gradient and the variations in the distance of the sample-sites from the vent center.

|  | **CC value (*r*)** | ***P* value** | **Rank** | **Benjamini-Hochberg critical value** |
| --- | --- | --- | --- | --- |
| ***Planctomycetes*** | 0.967 | 0.002 | 1 | 0.006896552 |
| ***Chlamydiae*** | 0.956 | 0.003 | 2 | 0.013793103 |
| ***Alphaproteobacteria*** | 0.944 | 0.005 | 3 | 0.020689655 |
| **Others** | 0.921 | 0.009 | 4 | 0.027586207 |
| **Unclassified *Proteobacteria*** | 0.902 | 0.014 | 5 | 0.034482759 |
| ***Betaproteobacteria*** | 0.898 | 0.015 | 6 | 0.04137931 |
| ***Gammaproteobacteria*** | 0.886 | 0.019 | 7 | 0.048275862 |
| ***Acidobacteria*** | 0.881 | 0.020 | 8 | 0.055172414 |
| ***Gemmatimonadetes*** | 0.878 | 0.021 | 9 | 0.062068966 |
| **Unclassified *Bacteria*** | 0.866 | 0.026 | 10 | 0.068965517 |
| ***Fusobacteria*** | 0.846 | 0.034 | 11 | 0.075862069 |
| ***Firmicutes*** | 0.844 | 0.035 | 12 | 0.082758621 |
| ***Deltaproteobacteria*** | 0.828 | 0.042 | 13 | 0.089655172 |
| ***Spirochaetes*** | 0.795 | 0.059 | 14 | 0.096551724 |
| ***Actinobacteria*** | 0.790 | 0.061 | 15 | 0.103448276 |
| ***Aquificae*** | -0.767 | 0.075 | 16 | 0.110344828 |
| ***Verrucomicrobia*** | 0.713 | 0.112 | 17 | 0.117241379 |
| ***Synergistetes*** | 0.703 | 0.119 | 18 | 0.124137931 |
| ***Tenericutes*** | 0.652 | 0.161 | 19 | 0.131034483 |
| ***Deinococcus-Thermus*** | -0.621 | 0.188 | 20 | 0.137931034 |
| ***Bacteroidetes*** | 0.585 | 0.222 | 21 | 0.144827586 |
| ***Thermotogae*** | -0.581 | 0.227 | 22 | 0.151724138 |
| ***Epsilonproteobacteria*** | -0.533 | 0.276 | 23 | 0.15862069 |
| ***Chlorobi*** | 0.389 | 0.446 | 24 | 0.165517241 |
| ***Nitrospirae*** | -0.378 | 0.460 | 25 | 0.172413793 |
| ***Chloroflexi*** | -0.357 | 0.488 | 26 | 0.179310345 |
| ***Dictyoglomi*** | -0.334 | 0.517 | 27 | 0.186206897 |
| ***Deferribacteres*** | -0.158 | 0.765 | 28 | 0.193103448 |
| ***Cyanobacteria*** | 0.123 | 0.816 | 29 | 0.2 |

^1^Phyla/classes were arranged in the ascending order of their *P* values (the one with the lowest *P* was ascribed rank 1); Benjamini-Hochberg critical values were determined as described in the Methods section; correlations were considered significant up to that phylum/class rank (see the phylum/class names that are in red font) where Benjamini-Hochberg critical value was still greater than the *P* value.

**Supplementary Table 12.** Benjamini-Hochberg correction^1^ of the *P* values obtained for individual Pearson correlation co-efficient (CC) values (*r*) between the phylum-/class-level population fluctuations along the wet thermal gradient and the variations in the flow-rate of hot water along the same trajectory.

|  | **CC value (*r*)** | ***P* value** | **Rank** | **Benjamini-Hochberg critical value** |
| --- | --- | --- | --- | --- |
| **Unclassified *Bacteria*** | -0.942 | 0.005 | 1 | 0.006896552 |
| ***Chlamydiae*** | -0.929 | 0.007 | 2 | 0.013793103 |
| ***Actinobacteria*** | -0.908 | 0.012 | 3 | 0.020689655 |
| ***Aquificae*** | 0.904 | 0.013 | 4 | 0.027586207 |
| ***Planctomycetes*** | -0.884 | 0.019 | 5 | 0.034482759 |
| ***Alphaproteobacteria*** | -0.874 | 0.023 | 6 | 0.04137931 |
| ***Firmicutes*** | -0.863 | 0.027 | 7 | 0.048275862 |
| **Others** | -0.854 | 0.030 | 8 | 0.055172414 |
| ***Gammaproteobacteria*** | -0.830 | 0.041 | 9 | 0.062068966 |
| **Unclassified *Proteobacteria*** | -0.824 | 0.044 | 10 | 0.068965517 |
| ***Fusobacteria*** | -0.811 | 0.050 | 11 | 0.075862069 |
| ***Gemmatimonadetes*** | -0.804 | 0.054 | 12 | 0.082758621 |
| ***Deinococcus-Thermus*** | 0.787 | 0.063 | 13 | 0.089655172 |
| ***Spirochaetes*** | -0.735 | 0.096 | 14 | 0.096551724 |
| ***Betaproteobacteria*** | -0.723 | 0.104 | 15 | 0.103448276 |
| ***Acidobacteria*** | -0.722 | 0.105 | 16 | 0.110344828 |
| ***Tenericutes*** | -0.721 | 0.106 | 17 | 0.117241379 |
| ***Epsilonproteobacteria*** | 0.709 | 0.114 | 18 | 0.124137931 |
| ***Deltaproteobacteria*** | -0.680 | 0.137 | 19 | 0.131034483 |
| ***Verrucomicrobia*** | -0.677 | 0.140 | 20 | 0.137931034 |
| ***Bacteroidetes*** | -0.640 | 0.171 | 21 | 0.144827586 |
| ***Synergistetes*** | -0.617 | 0.192 | 22 | 0.151724138 |
| ***Thermotogae*** | 0.557 | 0.251 | 23 | 0.15862069 |
| ***Chlorobi*** | -0.445 | 0.376 | 24 | 0.165517241 |
| ***Nitrospirae*** | 0.351 | 0.495 | 25 | 0.172413793 |
| ***Cyanobacteria*** | -0.306 | 0.556 | 26 | 0.179310345 |
| ***Dictyoglomi*** | 0.227 | 0.665 | 27 | 0.186206897 |
| ***Chloroflexi*** | 0.150 | 0.777 | 28 | 0.193103448 |
| ***Deferribacteres*** | 0.087 | 0.870 | 29 | 0.2 |

^1^ Phyla/classes were arranged in the ascending order of their *P* values (the one with the lowest *P* was ascribed rank 1); Benjamini-Hochberg critical values were determined as described in the Methods section; correlations were considered significant up to that phylum/class rank (see the phylum/class names that are in red font) where Benjamini-Hochberg critical value was still greater than the *P* value.

**Supplementary Table 13.** Calculation of ecological indices for VWM using phylum-/class-level distribution of shotgun metagenomic reads.

|  | **Dominance (*D*)*** | | **Shannon diversity index (*H*)****^#^** | |
| --- | --- | --- | --- | --- |
|  | **p_i_ = n_i_ / n** | **p_i_ ^2^** | **Ln p_i_** | **p_i_ (Ln p_i_)** |
| *Acidobacteria* | 0.001 | 0.203 | -6.536 | -0.009 |
| *Actinobacteria* | 0.004 | 0.000 | -5.547 | -0.022 |
| *Alphaproteobacteria* | 0.005 | 0.000 | -5.221 | -0.028 |
| *Aquificae* | 0.275 | 0.076 | -1.292 | -0.355 |
| *Bacteroidetes* | 0.004 | 0.603 | -5.534 | -0.022 |
| *Betaproteobacteria* | 0.005 | 0.000 | -5.404 | -0.024 |
| *Chlamydiae* | 0.000 | 0.003 | -8.294 | -0.002 |
| *Chlorobi* | 0.003 | 0.123 | -5.860 | -0.017 |
| *Chloroflexi* | 0.018 | 0.003 | -4.026 | -0.072 |
| *Cyanobacteria* | 0.009 | 0.723 | -4.694 | -0.043 |
| *Deferribacteres* | 0.001 | 0.000 | -6.645 | -0.009 |
| *Deinococcus-Thermus* | 0.595 | 0.354 | -0.520 | -0.309 |
| *Deltaproteobacteria* | 0.019 | 0.000 | -3.977 | -0.075 |
| *Dictyoglomi* | 0.001 | 0.000 | -6.812 | -0.007 |
| *Epsilonproteobacteria* | 0.007 | 0.000 | -4.906 | -0.036 |
| *Firmicutes* | 0.022 | 0.000 | -3.808 | -0.085 |
| *Fusobacteria* | 0.001 | 0.000 | -7.419 | -0.004 |
| *Gammaproteobacteria* | 0.009 | 0.323 | -4.727 | -0.042 |
| *Gemmatimonadetes* | 0.000 | 0.000 | -9.210 | -0.001 |
| *Nitrospirae* | 0.003 | 0.000 | -5.843 | -0.017 |
| Others | 0.001 | 0.000 | -7.601 | -0.004 |
| *Planctomycetes* | 0.001 | 0.063 | -7.195 | -0.005 |
| *Spirochaetes* | 0.001 | 0.000 | -7.013 | -0.006 |
| *Synergistetes* | 0.001 | 0.000 | -7.419 | -0.004 |
| *Tenericutes* | 0.000 | 0.000 | -8.112 | -0.002 |
| *Thermotogae* | 0.013 | 0.000 | -4.335 | -0.057 |
| unclassified *Bacteria* | 0.001 | 0.000 | -7.013 | -0.006 |
| unclassified *Proteobacteria* | 0.000 | 0.000 | -7.824 | -0.003 |
| *Verrucomicrobia* | 0.001 | 0.000 | -7.131 | -0.006 |
| ***D* = Σ p_i_ ^2^** |  | **0.431** |  |  |
| **Σ p_i_ (Ln p_i_)** |  |  |  | **-1.273** |
| ***H* = – Σ p_i_ (Ln p_i_)** |  |  |  | **1.273** |
| ***E_H_*= *H* / *H_max_*** |  |  |  | **0.378** |

* Mean percentage representations (relative abundance) of phyla/classes were directly used as their (n_i_ / n) values.

^#^ Shannon's equitability (*E_H_*) was obtained by dividing *H* by *H_max_*. *H_max_* is known to be Ln S, where S = total number of phyla/classes in the community. Thus, here *H_max_* = Ln 29 = 3.367.

**Supplementary Table 14.** Calculation of ecological indices for DG3 using phylum-/class-level distribution of shotgun metagenomic reads.

|  | **Dominance (*D*) *** | | **Shannon diversity index (*H*) ^#^** | |
| --- | --- | --- | --- | --- |
|  | **p_i_ = n_i_ / n** | **p_i_ ^2^** | **Ln p_i_** | **p_i_ (Ln p_i_)** |
| *Acidobacteria* | 0.008 | 0.000 | -4.880 | -0.037 |
| *Actinobacteria* | 0.022 | 0.000 | -3.837 | -0.083 |
| *Alphaproteobacteria* | 0.026 | 0.001 | -3.665 | -0.094 |
| *Aquificae* | 0.003 | 0.000 | -5.684 | -0.019 |
| *Bacteroidetes* | 0.048 | 0.002 | -3.044 | -0.145 |
| *Betaproteobacteria* | 0.017 | 0.000 | -4.080 | -0.069 |
| *Chlamydiae* | 0.001 | 0.063 | -7.195 | -0.005 |
| *Chlorobi* | 0.099 | 0.010 | -2.310 | -0.229 |
| *Chloroflexi* | 0.328 | 0.108 | -1.113 | -0.366 |
| *Cyanobacteria* | 0.287 | 0.083 | -1.247 | -0.358 |
| *Deferribacteres* | 0.001 | 0.003 | -7.070 | -0.006 |
| *Deinococcus-Thermus* | 0.022 | 0.000 | -3.808 | -0.085 |
| *Deltaproteobacteria* | 0.026 | 0.001 | -3.638 | -0.096 |
| *Dictyoglomi* | 0.001 | 0.000 | -7.419 | -0.004 |
| *Epsilonproteobacteria* | 0.002 | 0.000 | -6.166 | -0.013 |
| *Firmicutes* | 0.047 | 0.002 | -3.060 | -0.144 |
| *Fusobacteria* | 0.001 | 0.093 | -6.959 | -0.007 |
| *Gammaproteobacteria* | 0.028 | 0.001 | -3.568 | -0.101 |
| *Gemmatimonadetes* | 0.001 | 0.000 | -7.013 | -0.006 |
| *Nitrospirae* | 0.001 | 0.000 | -6.571 | -0.009 |
| Others | 0.002 | 0.403 | -6.470 | -0.010 |
| *Planctomycetes* | 0.005 | 0.003 | -5.269 | -0.027 |
| *Spirochaetes* | 0.007 | 0.000 | -4.906 | -0.036 |
| *Synergistetes* | 0.001 | 0.023 | -6.768 | -0.008 |
| *Tenericutes* | 0.000 | 0.123 | -7.958 | -0.003 |
| *Thermotogae* | 0.002 | 0.000 | -6.032 | -0.014 |
| unclassified *Bacteria* | 0.004 | 0.000 | -5.599 | -0.021 |
| unclassified *Proteobacteria* | 0.001 | 0.003 | -7.339 | -0.005 |
| *Verrucomicrobia* | 0.009 | 0.103 | -4.705 | -0.043 |
| ***D* = Σ p_i_ ^2^** |  | **0.208** |  |  |
| **Σ p_i_ (Ln p_i_)** |  |  |  | **-2.042** |
| ***H* = – Σ p_i_ (Ln p_i_)** |  |  |  | **2.042** |
| ***E_H_*= *H* / *H_max_*** |  |  |  | **0.606** |

* Mean percentage representations (relative abundance) of phyla/classes were directly used as their (n_i_ / n) values.

^#^ Shannon's equitability (*E_H_*) was obtained by dividing *H* by *H_max_*. *H_max_* is known to be Ln S, where S = total number of phyla/classes in the community. Thus, here *H_max_* = Ln 29 = 3.367.

**Supplementary Table 15.** Calculation of ecological indices for DG4 using phylum-/class-level distribution of shotgun metagenomic reads.

|  | **Dominance (*D*) *** | | **Shannon diversity index (*H*) ^#^** | |
| --- | --- | --- | --- | --- |
|  | **p_i_ = n_i_ / n** | **p_i_ ^2^** | **Ln p_i_** | **p_i_ (Ln p_i_)** |
| *Acidobacteria* | 0.012 | 0.000 | -4.457 | -0.052 |
| *Actinobacteria* | 0.050 | 0.003 | -2.991 | -0.150 |
| *Alphaproteobacteria* | 0.074 | 0.006 | -2.601 | -0.193 |
| *Aquificae* | 0.003 | 0.000 | -5.809 | -0.017 |
| *Bacteroidetes* | 0.042 | 0.002 | -3.168 | -0.133 |
| *Betaproteobacteria* | 0.028 | 0.001 | -3.574 | -0.100 |
| *Chlamydiae* | 0.001 | 0.156 | -6.685 | -0.008 |
| *Chlorobi* | 0.041 | 0.002 | -3.202 | -0.130 |
| *Chloroflexi* | 0.409 | 0.167 | -0.895 | -0.366 |
| *Cyanobacteria* | 0.077 | 0.006 | -2.559 | -0.198 |
| *Deferribacteres* | 0.001 | 0.103 | -6.859 | -0.007 |
| *Deinococcus-Thermus* | 0.026 | 0.001 | -3.654 | -0.095 |
| *Deltaproteobacteria* | 0.040 | 0.002 | -3.224 | -0.128 |
| *Dictyoglomi* | 0.001 | 0.103 | -6.859 | -0.007 |
| *Epsilonproteobacteria* | 0.002 | 0.003 | -6.012 | -0.015 |
| *Firmicutes* | 0.083 | 0.007 | -2.489 | -0.207 |
| *Fusobacteria* | 0.001 | 0.103 | -6.536 | -0.009 |
| *Gammaproteobacteria* | 0.067 | 0.004 | -2.702 | -0.181 |
| *Gemmatimonadetes* | 0.001 | 0.001 | -6.768 | -0.008 |
| *Nitrospirae* | 0.002 | 0.063 | -6.348 | -0.011 |
| Others | 0.002 | 0.203 | -6.190 | -0.013 |
| *Planctomycetes* | 0.009 | 0.000 | -4.699 | -0.043 |
| *Spirochaetes* | 0.005 | 0.003 | -5.231 | -0.028 |
| *Synergistetes* | 0.002 | 0.000 | -6.320 | -0.011 |
| *Tenericutes* | 0.001 | 0.023 | -7.339 | -0.005 |
| *Thermotogae* | 0.004 | 0.000 | -5.534 | -0.022 |
| unclassified *Bacteria* | 0.008 | 0.000 | -4.816 | -0.039 |
| unclassified *Proteobacteria* | 0.001 | 0.000 | -6.908 | -0.007 |
| *Verrucomicrobia* | 0.007 | 0.692 | -4.984 | -0.034 |
| ***D* = Σ p_i_ ^2^** |  | **0.199** |  |  |
| **Σ p_i_ (Ln p_i_)** |  |  |  | **-2.218** |
| ***H* = – Σ p_i_ (Ln p_i_)** |  |  |  | **2.218** |
| ***E_H_*= *H* / *H_max_*** |  |  |  | **0.659** |

* Mean percentage representations (relative abundance) of phyla/classes were directly used as their (n_i_ / n) values.

^#^ Shannon's equitability (*E_H_*) was obtained by dividing *H* by *H_max_*. *H_max_* is known to be Ln S, where S = total number of phyla/classes in the community. Thus, here *H_max_* = Ln 29 = 3.367.

**Supplementary Table 16.** Calculation of ecological indices for WG3 using phylum-/class-level distribution of shotgun metagenomic reads.

|  | **Dominance (*D*) *** | | **Shannon diversity index (*H*) ^#^** | |
| --- | --- | --- | --- | --- |
|  | **p_i_ = n_i_ / n** | **p_i_ ^2^** | **Ln p_i_** | **p_i_ (Ln p_i_)** |
| *Acidobacteria* | 0.007 | 0.001 | -4.998 | -0.034 |
| *Actinobacteria* | 0.014 | 0.000 | -4.279 | -0.059 |
| *Alphaproteobacteria* | 0.016 | 0.000 | -4.110 | -0.067 |
| *Aquificae* | 0.114 | 0.013 | -2.168 | -0.248 |
| *Bacteroidetes* | 0.032 | 0.001 | -3.440 | -0.110 |
| *Betaproteobacteria* | 0.024 | 0.001 | -3.736 | -0.089 |
| *Chlamydiae* | 0.001 | 0.000 | -7.264 | -0.005 |
| *Chlorobi* | 0.037 | 0.001 | -3.286 | -0.123 |
| *Chloroflexi* | 0.362 | 0.131 | -1.016 | -0.368 |
| *Cyanobacteria* | 0.127 | 0.016 | -2.067 | -0.262 |
| *Deferribacteres* | 0.001 | 0.000 | -6.571 | -0.009 |
| *Deinococcus-Thermus* | 0.118 | 0.014 | -2.135 | -0.252 |
| *Deltaproteobacteria* | 0.027 | 0.001 | -3.605 | -0.098 |
| *Dictyoglomi* | 0.001 | 0.003 | -7.070 | -0.006 |
| *Epsilonproteobacteria* | 0.005 | 0.003 | -5.371 | -0.025 |
| *Firmicutes* | 0.038 | 0.001 | -3.260 | -0.125 |
| *Fusobacteria* | 0.001 | 0.000 | -6.908 | -0.007 |
| *Gammaproteobacteria* | 0.039 | 0.002 | -3.243 | -0.127 |
| *Gemmatimonadetes* | 0.001 | 0.000 | -7.264 | -0.005 |
| *Nitrospirae* | 0.002 | 0.003 | -6.190 | -0.013 |
| Others | 0.002 | 0.001 | -6.470 | -0.010 |
| *Planctomycetes* | 0.004 | 0.000 | -5.573 | -0.021 |
| *Spirochaetes* | 0.004 | 0.003 | -5.534 | -0.022 |
| *Synergistetes* | 0.001 | 0.000 | -6.812 | -0.007 |
| *Tenericutes* | 0.000 | 0.000 | -7.824 | -0.003 |
| *Thermotogae* | 0.006 | 0.000 | -5.167 | -0.029 |
| unclassified *Bacteria* | 0.003 | 0.000 | -5.793 | -0.018 |
| unclassified *Proteobacteria* | 0.001 | 0.003 | -7.506 | -0.004 |
| *Verrucomicrobia* | 0.012 | 0.000 | -4.398 | -0.054 |
| ***D* = Σ p_i_ ^2^** |  | **0.182** |  |  |
| **Σ p_i_ (Ln p_i_)** |  |  |  | **-2.202** |
| ***H* = – Σ p_i_ (Ln p_i_)** |  |  |  | **2.202** |
| ***E_H_*= *H* / *H_max_*** |  |  |  | **0.654** |

* Mean percentage representations (relative abundance) of phyla/classes were directly used as their (n_i_ / n) values.

^#^ Shannon's equitability (*E_H_*) was obtained by dividing *H* by *H_max_*. *H_max_* is known to be Ln S, where S = total number of phyla/classes in the community. Thus, here *H_max_* = Ln 29 = 3.367.

**Supplementary Table 17.** Calculation of ecological indices for WG4 using phylum-/class-level distribution of shotgun metagenomic reads.

|  | **Dominance (*D*) *** | | **Shannon diversity index (*H*) ^#^** | |
| --- | --- | --- | --- | --- |
|  | **p_i_ = n_i_ / n** | **p_i_ ^2^** | **Ln p_i_** | **p_i_ (Ln p_i_)** |
| *Acidobacteria* | 0.007 | 0.003 | -4.969 | -0.035 |
| *Actinobacteria* | 0.017 | 0.000 | -4.083 | -0.069 |
| *Alphaproteobacteria* | 0.018 | 0.000 | -4.029 | -0.072 |
| *Aquificae* | 0.097 | 0.009 | -2.331 | -0.227 |
| *Bacteroidetes* | 0.055 | 0.003 | -2.892 | -0.160 |
| *Betaproteobacteria* | 0.015 | 0.000 | -4.227 | -0.062 |
| *Chlamydiae* | 0.001 | 0.003 | -7.070 | -0.006 |
| *Chlorobi* | 0.082 | 0.007 | -2.502 | -0.205 |
| *Chloroflexi* | 0.263 | 0.069 | -1.334 | -0.351 |
| *Cyanobacteria* | 0.193 | 0.037 | -1.646 | -0.317 |
| *Deferribacteres* | 0.004 | 0.000 | -5.655 | -0.020 |
| *Deinococcus-Thermus* | 0.080 | 0.006 | -2.526 | -0.202 |
| *Deltaproteobacteria* | 0.033 | 0.001 | -3.427 | -0.111 |
| *Dictyoglomi* | 0.003 | 0.003 | -5.860 | -0.017 |
| *Epsilonproteobacteria* | 0.005 | 0.003 | -5.308 | -0.026 |
| *Firmicutes* | 0.050 | 0.002 | -2.998 | -0.150 |
| *Fusobacteria* | 0.001 | 0.000 | -6.571 | -0.009 |
| *Gammaproteobacteria* | 0.028 | 0.001 | -3.561 | -0.101 |
| *Gemmatimonadetes* | 0.001 | 0.000 | -7.131 | -0.006 |
| *Nitrospirae* | 0.005 | 0.000 | -5.350 | -0.025 |
| Others | 0.002 | 0.003 | -6.240 | -0.012 |
| *Planctomycetes* | 0.004 | 0.000 | -5.521 | -0.022 |
| *Spirochaetes* | 0.007 | 0.000 | -5.036 | -0.033 |
| *Synergistetes* | 0.002 | 0.000 | -6.470 | -0.010 |
| *Tenericutes* | 0.001 | 0.003 | -7.506 | -0.004 |
| *Thermotogae* | 0.018 | 0.000 | -4.023 | -0.072 |
| unclassified *Bacteria* | 0.004 | 0.000 | -5.655 | -0.020 |
| unclassified *Proteobacteria* | 0.001 | 0.003 | -7.506 | -0.004 |
| *Verrucomicrobia* | 0.007 | 0.000 | -4.962 | -0.035 |
| ***D* = Σ p_i_ ^2^** |  | **0.138** |  |  |
| **Σ p_i_ (Ln p_i_)** |  |  |  | **-2.382** |
| ***H* = – Σ p_i_ (Ln p_i_)** |  |  |  | **2.382** |
| ***E_H_*= *H* / *H_max_*** |  |  |  | **0.708** |

* Mean percentage representations (relative abundance) of phyla/classes were directly used as their (n_i_ / n) values.

^#^ Shannon's equitability (*E_H_*) was obtained by dividing *H* by *H_max_*. *H_max_* is known to be Ln S, where S = total number of phyla/classes in the community. Thus, here *H_max_* = Ln 29 = 3.367.

**Supplementary Table 18.** Calculation of ecological indices for WG5 using phylum-/class-level distribution of shotgun metagenomic reads.

|  | **Dominance (*D*) *** | | **Shannon diversity index (*H*) ^#^** | |
| --- | --- | --- | --- | --- |
|  | **p_i_ = n_i_ / n** | **p_i_ ^2^** | **Ln p_i_** | **p_i_ (Ln p_i_)** |
| *Acidobacteria* | 0.007 | 0.002 | -4.913 | -0.036 |
| *Actinobacteria* | 0.027 | 0.001 | -3.623 | -0.097 |
| *Alphaproteobacteria* | 0.030 | 0.001 | -3.523 | -0.104 |
| *Aquificae* | 0.002 | 0.000 | -6.240 | -0.012 |
| *Bacteroidetes* | 0.033 | 0.001 | -3.404 | -0.113 |
| *Betaproteobacteria* | 0.019 | 0.000 | -3.982 | -0.074 |
| *Chlamydiae* | 0.001 | 0.002 | -7.070 | -0.006 |
| *Chlorobi* | 0.032 | 0.001 | -3.428 | -0.111 |
| *Chloroflexi* | 0.329 | 0.108 | -1.112 | -0.366 |
| *Cyanobacteria* | 0.361 | 0.130 | -1.020 | -0.368 |
| *Deferribacteres* | 0.001 | 0.000 | -7.264 | -0.005 |
| *Deinococcus-Thermus* | 0.013 | 0.000 | -4.370 | -0.055 |
| *Deltaproteobacteria* | 0.028 | 0.001 | -3.560 | -0.101 |
| *Dictyoglomi* | 0.001 | 0.000 | -7.419 | -0.004 |
| *Epsilonproteobacteria* | 0.002 | 0.000 | -6.215 | -0.012 |
| *Firmicutes* | 0.047 | 0.002 | -3.051 | -0.144 |
| *Fusobacteria* | 0.001 | 0.000 | -7.070 | -0.006 |
| *Gammaproteobacteria* | 0.033 | 0.001 | -3.427 | -0.111 |
| *Gemmatimonadetes* | 0.001 | 0.000 | -7.264 | -0.005 |
| *Nitrospirae* | 0.001 | 0.000 | -6.645 | -0.009 |
| Others | 0.002 | 0.000 | -6.470 | -0.010 |
| *Planctomycetes* | 0.006 | 0.000 | -5.185 | -0.029 |
| *Spirochaetes* | 0.010 | 0.000 | -4.595 | -0.046 |
| *Synergistetes* | 0.001 | 0.003 | -6.859 | -0.007 |
| *Tenericutes* | 0.000 | 0.002 | -7.958 | -0.003 |
| *Thermotogae* | 0.002 | 0.063 | -6.097 | -0.014 |
| unclassified *Bacteria* | 0.004 | 0.003 | -5.485 | -0.023 |
| unclassified *Proteobacteria* | 0.001 | 0.000 | -7.013 | -0.006 |
| *Verrucomicrobia* | 0.007 | 0.003 | -4.927 | -0.036 |
| ***D* = Σ p_i_ ^2^** |  | **0.247** |  |  |
| **Σ p_i_ (Ln p_i_)** |  |  |  | **-1.915** |
| ***H* = – Σ p_i_ (Ln p_i_)** |  |  |  | **1.915** |
| ***E_H_*= *H* / *H_max_*** |  |  |  | **0.569** |

* Mean percentage representations (relative abundance) of phyla/classes were directly used as their (n_i_ / n) values.

^#^ Shannon's equitability (*E_H_*) was obtained by dividing *H* by *H_max_*. *H_max_* is known to be Ln S, where S = total number of phyla/classes in the community. Thus, here *H_max_* = Ln 29 = 3.367.

**Supplementary Table 19.** Calculation of ecological indices for WG6 using phylum-/class-level distribution of shotgun metagenomic reads.

|  | **Dominance (*D*) *** | | **Shannon diversity index (*H*) ^#^** | |
| --- | --- | --- | --- | --- |
|  | **p_i_ = n_i_ / n** | **p_i_ ^2^** | **Ln p_i_** | **p_i_ (Ln p_i_)** |
| *Acidobacteria* | 0.009 | 0.000 | -4.756 | -0.041 |
| *Actinobacteria* | 0.022 | 0.000 | -3.819 | -0.084 |
| *Alphaproteobacteria* | 0.097 | 0.009 | -2.332 | -0.226 |
| *Aquificae* | 0.002 | 0.000 | -6.240 | -0.012 |
| *Bacteroidetes* | 0.500 | 0.250 | -0.694 | -0.347 |
| *Betaproteobacteria* | 0.034 | 0.001 | -3.383 | -0.115 |
| *Chlamydiae* | 0.001 | 0.002 | -6.608 | -0.009 |
| *Chlorobi* | 0.024 | 0.001 | -3.726 | -0.090 |
| *Chloroflexi* | 0.090 | 0.008 | -2.414 | -0.216 |
| *Cyanobacteria* | 0.034 | 0.001 | -3.389 | -0.114 |
| *Deferribacteres* | 0.001 | 0.000 | -6.908 | -0.007 |
| *Deinococcus-Thermus* | 0.007 | 0.000 | -4.962 | -0.035 |
| *Deltaproteobacteria* | 0.028 | 0.001 | -3.588 | -0.099 |
| *Dictyoglomi* | 0.000 | 0.000 | -7.706 | -0.003 |
| *Epsilonproteobacteria* | 0.004 | 0.000 | -5.521 | -0.022 |
| *Firmicutes* | 0.046 | 0.002 | -3.081 | -0.141 |
| *Fusobacteria* | 0.002 | 0.000 | -6.438 | -0.010 |
| *Gammaproteobacteria* | 0.055 | 0.003 | -2.895 | -0.160 |
| *Gemmatimonadetes* | 0.001 | 0.002 | -7.070 | -0.006 |
| *Nitrospirae* | 0.001 | 0.000 | -6.725 | -0.008 |
| Others | 0.002 | 0.003 | -6.012 | -0.015 |
| *Planctomycetes* | 0.015 | 0.000 | -4.177 | -0.064 |
| *Spirochaetes* | 0.004 | 0.000 | -5.534 | -0.022 |
| *Synergistetes* | 0.001 | 0.000 | -6.959 | -0.007 |
| *Tenericutes* | 0.001 | 0.000 | -7.419 | -0.004 |
| *Thermotogae* | 0.002 | 0.000 | -6.320 | -0.011 |
| unclassified *Bacteria* | 0.005 | 0.000 | -5.360 | -0.025 |
| unclassified *Proteobacteria* | 0.001 | 0.000 | -7.264 | -0.005 |
| *Verrucomicrobia* | 0.013 | 0.000 | -4.358 | -0.056 |
| ***D* = Σ p_i_ ^2^** |  | **0.277** |  |  |
| **Σ p_i_ (Ln p_i_)** |  |  |  | **-1.955** |
| ***H* = – Σ p_i_ (Ln p_i_)** |  |  |  | **1.955** |
| ***E_H_*= *H* / *H_max_*** |  |  |  | **0.581** |

* Mean percentage representations (relative abundance) of phyla/classes were directly used as their (n_i_ / n) values.

^#^ Shannon's equitability (*E_H_*) was obtained by dividing *H* by *H_max_*. *H_max_* is known to be Ln S, where S = total number of phyla/classes in the community. Thus, here *H_max_* = Ln 29 = 3.367.

**Supplementary Table 20.** Calculation of ecological indices for WG7 using phylum-/class-level distribution of shotgun metagenomic reads.

|  | **Dominance (*D*) *** | | **Shannon diversity index (*H*) ^#^** | |
| --- | --- | --- | --- | --- |
|  | **p_i_ = n_i_ / n** | **p_i_ ^2^** | **Ln p_i_** | **p_i_ (Ln p_i_)** |
| *Acidobacteria* | 0.027 | 0.001 | -3.599 | -0.098 |
| *Actinobacteria* | 0.026 | 0.001 | -3.646 | -0.095 |
| *Alphaproteobacteria* | 0.103 | 0.011 | -2.269 | -0.235 |
| *Aquificae* | 0.003 | 0.000 | -5.714 | -0.019 |
| *Bacteroidetes* | 0.167 | 0.028 | -1.791 | -0.299 |
| *Betaproteobacteria* | 0.085 | 0.007 | -2.468 | -0.209 |
| *Chlamydiae* | 0.002 | 0.000 | -6.377 | -0.011 |
| *Chlorobi* | 0.071 | 0.005 | -2.649 | -0.187 |
| *Chloroflexi* | 0.074 | 0.006 | -2.600 | -0.193 |
| *Cyanobacteria* | 0.156 | 0.024 | -1.860 | -0.290 |
| *Deferribacteres* | 0.002 | 0.002 | -6.407 | -0.011 |
| *Deinococcus-Thermus* | 0.009 | 0.000 | -4.689 | -0.043 |
| *Deltaproteobacteria* | 0.063 | 0.004 | -2.759 | -0.175 |
| *Dictyoglomi* | 0.001 | 0.002 | -7.070 | -0.006 |
| *Epsilonproteobacteria* | 0.004 | 0.002 | -5.560 | -0.021 |
| *Firmicutes* | 0.067 | 0.004 | -2.708 | -0.181 |
| *Fusobacteria* | 0.002 | 0.000 | -6.240 | -0.012 |
| *Gammaproteobacteria* | 0.068 | 0.005 | -2.691 | -0.182 |
| *Gemmatimonadetes* | 0.002 | 0.000 | -6.377 | -0.011 |
| *Nitrospirae* | 0.002 | 0.000 | -6.119 | -0.013 |
| Others | 0.004 | 0.000 | -5.560 | -0.021 |
| *Planctomycetes* | 0.019 | 0.000 | -3.950 | -0.076 |
| *Spirochaetes* | 0.016 | 0.000 | -4.110 | -0.067 |
| *Synergistetes* | 0.002 | 0.000 | -6.097 | -0.014 |
| *Tenericutes* | 0.001 | 0.000 | -7.506 | -0.004 |
| *Thermotogae* | 0.004 | 0.000 | -5.613 | -0.020 |
| unclassified *Bacteria* | 0.005 | 0.000 | -5.240 | -0.028 |
| unclassified *Proteobacteria* | 0.001 | 0.000 | -6.685 | -0.008 |
| *Verrucomicrobia* | 0.015 | 0.000 | -4.186 | -0.064 |
| ***D* = Σ p_i_ ^2^** |  | **0.096** |  |  |
| **Σ p_i_ (Ln p_i_)** |  |  |  | **-2.594** |
| ***H* = – Σ p_i_ (Ln p_i_)** |  |  |  | **2.594** |
| ***E_H_*= *H* / *H_max_*** |  |  |  | **0.770** |

* Mean percentage representations (relative abundance) of phyla/classes were directly used as their (n_i_ / n) values.

^#^ Shannon's equitability (*E_H_*) was obtained by dividing *H* by *H_max_*. *H_max_* is known to be Ln S, where S = total number of phyla/classes in the community. Thus, here *H_max_* = Ln 29 = 3.367.

**Supplementary Table 21.** Genera collectively accounting for average 50% of all classifiable reads (after searching against the nr protein sequence database) in the metagenomes of the mat communities VWM, DG3 and DG4 (names are in the descending order of their relative abundance within the community).

| **VWM*** | **DG3^$^** | **DG4^#^** |
| --- | --- | --- |
| *Thermus* | *Roseiflexus, Flavobacterium, Thermosynechococcus, Chloroflexus, Chloroherpeton, Cyanothece, Synechococcus, Oscillochloris, Chlorobium, Anabaena, Nostoc, Synechocystis* | *Roseiflexus, Chloroflexus, Oscillochloris, Herpetosiphon, Anaerolinea, Pseudomonas, Chloroherpeton, Synechococcus, Chlorobium, Bacillus, Cyanothece, Sphaerobacter, Anabaena, Nostoc, Xanthomonas, Paracoccus, Clostridium, Geobacter, Ktedonobacter, Deinococcus, Meiothermus, Thermus, Thermobaculum, Streptomyces, Rubrobacter, Candidatus Solibacter, Truepera, Chlorobaculum, Rhodobacter, Thermomicrobium, Burkholderia, Symbiobacterium, Anaeromyxobacter, Rhodothermus, Geobacillus, Myxococcus, Paenibacillus, Mycobacterium, Desulfovibrio, Thermosynechococcus, Dehalococcoides, Synechocystis, Pelodictyon, Stenotrophomonas, Microcoleus, Gloeobacter, Cytophaga, Bradyrhizobium, Candidatus Koribacter, Spirosoma* |

* Only one genus accounted for an average 50.64 % of all classifiable reads in VWM.

^$^ 12 genera accounted for an average 50.12 % of all classifiable reads in DG3.

^#^ 50 one genus accounted for an average 50.11 % of all classifiable reads in DG4.

**Supplementary Table 22.** Genera collectively accounting for average 50% of all classifiable reads (after searching against the nr protein sequence database) in the metagenomes of WG3, WG4, WG5, WG6 and WG7 (names are in the descending order of their relative abundance within the community).

| **WG3^1^** | **WG4^2^** | **WG5^3^** | **WG6^4^** | **WG7^5^** |
| --- | --- | --- | --- | --- |
| *Roseiflexus, Thermus, Sulfurihydrogenibium, Thermosynechococcus, Chloroflexus, Oscillochloris, Cyanothece, Chloroherpeton, Synechococcus,*  *Chlorobium, Aquifex* | *Roseiflexus, Thermosynechococcus, Sulfurihydrogenibium, Thermus, Chloroflexus, Chloroherpeton, Cyanothece, Oscillochloris, Chlorobium, Anaerolinea, Synechococcus, Thermotoga, Aquifex, Anabaena, Nostoc, Bacteroides, Meiothermus* | *Roseiflexus, Chloroflexus, Synechococcus, Cyanothece, Oscillochloris, Anabaena, Nostoc, Thermosynechococcus, Herpetosiphon, Synechocystis, Thermus, Sulfurihydrogenibium, Meiothermus, Aquifex, Thermotoga, Deinococcus, Geobacter, Oceanithermus, Chloroherpeton, Chlorobium, Anaerolinea, Pseudomonas, Bacillus, Flavobacterium, Gramella, Chitinophaga, Bacteroides, Pedobacter, Spirosoma, Cytophaga, Maribacter, Trichodesmium, Microcystis, Arthrospira, Microcoleus* | *Flavobacterium, Roseiflexus, Gramella, Chitinophaga, Bacteroides, Pedobacter, Chloroflexus, Spirosoma, Cytophaga, Maribacter, Dyadobacter, Polaribacter, Robiginitalea, Microscilla, Oscillochloris, Croceibacter, Capnocytophaga, Leeuwenhoekiella, Marivirga, Zunongwangia, Dokdonia, Chlorobium, Algoriphagus, Cellulophaga, Kordia, Bacillus, Chloroherpeton, Prevotella, Parabacteroides, Leadbetterella, Sphingobacterium, Herpetosiphon, Rhodobacter, Psychroflexus, Synechococcus, Nitrosomonas, Anaerolinea, Mucilaginibacter, Cyanothece, Candidatus Solibacter,*  *Clostridium, Porphyromonas, Rhodothermus, Chryseobacterium, Geobacter, Pseudomonas, Riemerella, Rhodopirellula, Burkholderia, Shewanella, Salinibacter, Sphingomonas, Anabaena, Nostoc, Planctomyces, Paludibacter, Roseobacter, Alistipes, Ruegeria, Magnetospirillum, Rhodospirillum*  *Desulfovibrio, Chlorobaculum, Xanthomonas, Vibrio, Streptomyces, Chthoniobacter, Sphaerobacter, Pelodictyon* | *Roseiflexus, Chloroherpeton, Synechococcus, Cyanothece, Candidatus Solibacter, Chlorobium, Chloroflexus, Anabaena, Hyphomonas, Nostoc, Thermosynechococcus, Bacteroides, Burkholderia, Desulfovibrio, Anaerolinea, Clostridium, Pedobacter, Geobacter, Flavobacterium, Oscillochloris, Cytophaga, Chitinophaga, Spirosoma, Chlorobaculum, Bacillus, Synechocystis, Pseudomonas, Herpetosiphon, Cupriavidus, Dyadobacter, Trichodesmium, Gramella, Ralstonia, Pelodictyon, Rhodobacter, Microscilla, Bordetella, Magnetospirillum, Anaeromyxobacter, Treponema, Planctomyces, Rhodothermus, Rhodospirillum, Acidovorax, Leptospira, Candidatus Koribacter, Prosthecochloris, Rhodopseudomonas, Spirochaeta, Parabacteroides, Marivirga, Prevotella, Rhodopirellula, Syntrophobacter, Microcoleus, Gloeobacter, Chthoniobacter, Polaromonas, Microcystis, Bradyrhizobium, Salinibacter, Methylobacterium, Acaryochloris, Shewanella, Pelobacter, Arthrospira, Sphingomonas, Robiginitalea, Myxococcus, Thiobacillus, Streptomyces, Thioalkalivibrio, Nitrosomonas, Methylibium, Oscillatoria, Aromatoleum, Nitrosococcus, Nodularia, Blastopirellula, Sphingobacterium, Leadbetterella* |

^1^ 11 genera accounted for an average 50.16 % of all classifiable reads in WG3.

^2^ 17 one genus accounted for an average 50.3 % of all classifiable reads in WG4.

^3^ 35 genera accounted for an average 50.32 % of all classifiable reads in WG5.

^4^ 68 one genus accounted for an average 50.18 % of all classifiable reads in WG6.

^5^ 81 genera accounted for an average 50.56 % of all classifiable reads in WG7.

**Supplementary Table 23.** Total number of genera identified in VWM after searching both its metagenomic readsets against the RDP database.

| **Name of the phylum** | **Number of genera identified** | **Names of genera identified** |
| --- | --- | --- |
| *Actinobacteria* | 5 | *Actinoplanes, Saccharopolyspora, Thermobispora, Tropheryma, Thermobifida* |
| *Aquificae* | 5 | *Sulfurihydrogenibium, Venenivibrio, Persephonella, Hydrogenobaculum, Desulfurobacterium* |
| *Bacteroidetes* | 4 | *Alistipes, Cytophaga, Rhodothermus, Salinibacter* |
| *Chloroflexi* | 5 | *Chloroflexus, Roseiflexus, Herpetosiphon, Dehalococcoides, Thermomicrobium* |
| *Cyanobacteria* | 2 | *Synechococcus, Oscillatoria* |
| *Deinococcus-Thermus* | 2 | *Thermus, Meiothermus* |
| *Dictyoglomi* | 1 | *Dictyoglomus* |
| *Firmicutes* | 12 | *Anoxybacillus, Bacillus, Geobacillus, Paenibacillus, Trichococcus, Lactobacillus, Clostridium, Caldicellulosiruptor, Coprothermobacter, Thermodesulfobium, Streptococcus, Carboxydothermus* |
| *Nitrospirae* | 1 | *Thermodesulfovibrio* |
| *Proteobacteria* | 7 | *Pseudomonas, Blastochloris, Thiobacillus, Acidithiobacillus, Microbulbifer, Campylobacter, Thiohalocapsa* |
| *Spirochaetes* | 1 | *Treponema* |
| *Tenericutes* | 1 | *Acholeplasma* |
| *Thermodesulfobacteria* | 2 | *Thermodesulfobacterium, Thermodesulfatator* |
| *Thermotogae* | 3 | *Thermotoga, Thermosipho, Fervidobacterium* |

**Supplementary Table 24.** Total number of genera identified in DG3 after searching both its metagenomic readsets against the RDP database.

| **Name of the phylum** | **Number of genera identified** | **Names of genera identified** |
| --- | --- | --- |
| *Acidobacteria* | 1 | *Candidatus* Solibacter |
| *Actinobacteria* | 11 | *Actinoplanes, Atopobium, Propionibacterium, Streptomyces, Frankia, Curtobacterium, Rathayibacter, Renibacterium, Nocardia, Kitasatospora, Thermoleophilum* |
| *Aquificae* | 1 | *Sulfurihydrogenibium* |
| *Bacteroidetes* | 21 | *Alistipes, Cytophaga, Flectobacillus, Flexibacter, Hymenobacter, Microscilla, Spirosoma, Flammeovirga, Flexithrix, Capnocytophaga, Croceibacter, Elizabethkingia, Flavobacterium, Gramella, Myroides, Tenacibaculum, Sphingobacterium, Chitinophaga, Prolixibacter, Marinoscillum, Wautersiella* |
| *Chlorobi* | 4 | *Chlorobaculum, Prosthecochloris, Chlorobium, Chloroherpeton* |
| *Chloroflexi* | 7 | *Chloroflexus, Roseiflexus, Oscillochloris, Herpetosiphon, Dehalococcoides, Sphaerobacter, Thermomicrobium* |
| *Cyanobacteria* | 22 | *Cyanothece, Gloeocapsopsis, Microcystis, Synechococcus, Anabaena, Dolichospermum, Nodularia, Calothrix, Scytonema, Geitlerinema, Oscillatoria, Planktothrix, Pseudanabaena, Spirulina, Chroococcidiopsis, Pleurocapsa, Stanieria, Xenococcus, Prochlorothrix, Thermosynechococcus, Leptolyngbya, Aphanizomenon* |
| *Deinococcus-Thermus* | 2 | *Deinococcus,Thermus* |
| *Firmicutes* | 16 | *Bacillus, Sporosarcina, Lysinibacillus, Planomicrobium, Kurthia, Faecalibacterium, Listeria, Brevibacillus, Leuconostoc, Oxobacter, Desulfotomaculum, Alicyclobacillus*  *Laceyella, Clostridium, Ammonifex, Thermodesulfobium* |
| *Planctomycetes* | 1 | *Isosphaera* |
| *Proteobacteria* | 16 | *Methylobacterium, Paracoccus, Rubritepida, Bdellovibrio, Desulfomicrobium, Arcobacter, Thiocapsa, Actinobacillus, Acinetobacter, Thiothrix, Helicobacter, Campylobacter, Burkholderia, Desulfocella, Acidithiobacillus, Pseudomonas* |
| *Spirochaetes* | 4 | *Leptonema, Leptospira, Spirochaeta, Treponema* |
| *Tenericutes* | 1 | *Mycloplasma* |
| *Thermotogae* | 1 | *Thermotoga* |
| *Verrucomicrobia* | 4 | *Diplosphaera, Chthoniobacter, Verrucomicrobium, Methylacidiphilum* |

**Supplementary Table 25.** Total number of genera identified in DG4 after searching both its metagenomic readsets against the RDP database.

| **Name of the phylum** | **Number of genera identified** | **Names of genera identified** |
| --- | --- | --- |
| *Actinobacteria* | 9 | *Gordonia, Microbacterium, Rhodococcus, Rathayibacter, Streptomyces, Atopobium, Rubrobacter, Tropheryma* |
| *Aquificae* | 2 | *Sulfurihydrogenibium, Persephonella* |
| *Bacteroidetes* | 24 | *Bacteroides, Odoribacter, Parabacteroides, Tannerella, Alistipes, Cytophaga, Flexibacter, Hymenobacter, Spirosoma, Flexithrix, Capnocytophaga, Elizabethkingia, Gramella, Tenacibaculum, Wautersiella, Saprospira, Pedobacter, Chitinophaga, Prolixibacter, Porphyromonas, Cyclobacterium, Flectobacillus, Microscilla, Cellulophaga* |
| *Chlorobi* | 4 | *Chlorobium, Prosthecochloris, Chlorobaculum, Chloroherpeton* |
| *Chloroflexi* | 8 | *Roseiflexus, Oscillochloris, Dehalococcoides, Herpetosiphon, Chloroflexus, Thermomicrobium, Sphaerobacter, Ktedonobacter* |
| *Cyanobacteria* | 17 | *Cyanobacterium, Cyanothece, Gloeocapsopsis, Microcystis, Synechococcus, Calothrix, Scytonema, Geitlerinema, Leptolyngbya, Microcoleus, Oscillatoria, Phormidium, Spirulina, Chroococcidiopsis, Chlorogloeopsis, Fischerella, Nostoc* |
| *Deinococcus-Thermus* | 3 | *Deinococcus, Truepera, Thermus* |
| *Firmicutes* | 14 | *Alicyclobacillus, Bacillus, Laceyella, Exiguobacterium, Leuconostoc, Streptococcus, Clostridium, Anaerococcus, Butyrivibrio, Desulfosporosinus, Ruminococcus, Syntrophomonas, Staphylococcus, Lactococcus* |
| *Planctomycetes* | 1 | *Isosphaera* |
| *Proteobacteria* | 31 | *Bradyrhizobium, Alishewanella, Azospirillum, Desulfovibrio, Enterobacter, Escherichia, Kinetoplastibacterium, Kingella, Novosphingobium, Paracoccus, Phyllobacterium, Pseudomonas, Rhodobaca, Rhodobacter, Roseisalinus, Roseococcus, Rubritepida, Shigella, Sphingopyxis, Stenotrophomonas, Thiocapsa, Thiothrix, Xanthomonas, Xylella, Desulfocella, Azotobacter, Helicobacter, Methylarcula, Nitrosovibrio, Sorangium, Thiorhodococcus* |
| *Spirochaetes* | 3 | *Leptospira, Spirochaeta, Treponema* |
| *Tenericutes* | 1 | *Acholeplasma* |
| *Thermodesulfobacteria* | 1 | *Thermodesulfobacterium* |
| *Thermotogae* | 1 | *Fervidobacterium* |
| *Verrucomicrobia* | 2 | *Pedosphaera, Methylacidiphilum* |

**Supplementary Table 26.** Total number of genera identified in WG3 after searching both its metagenomic readsets against the RDP database.

| **Name of the phylum** | **Number of genera identified** | **Names of genera identified** |
| --- | --- | --- |
| *Actinobacteria* | 6 | *Thermobispora, Enterorhabdus, Actinoplanes, Propionibacterium, Kitasatospora, Streptomyces* |
| *Aquificae* | 3 | *Persephonella, Sulfurihydrogenibium, Venenivibrio* |
| *Bacteroidetes* | 9 | *Hymenobacter, Rhodothermus, Flexithrix, Bacteroides, Alistipes, Salinibacter, Porphyromonas, Blattabacterium, Flavobacterium* |
| *Chlamydiae* | 2 | *Chlamydophila, Criblamydia* |
| *Chlorobi* | 4 | *Prosthecochloris, Chlorobaculum, Chloroherpeton, Chlorobium* |
| *Chloroflexi* | 7 | *Chloroflexus, Roseiflexus, Oscillochloris, Dehalococcoides, Ktedonobacter, Thermomicrobium, Herpetosiphon* |
| *Cyanobacteria* | 8 | *Synechococcus, Thermosynechococcus, Dolichospermum, Gloeobacter, Cylindrospermopsis, Nostoc, Planktothrix, Prochlorothrix* |
| *Deinococcus-Thermus* | 2 | *Thermus, Meiothermus* |
| *Firmicutes* | 11 | *Alicyclobacillus, Bacillus, Geobacillus, Aneurinibacillus, Paenibacillus, Lactobacillus, Leuconostoc, Clostridium, Butyrivibrio, Caldicellulosiruptor, Brevibacillus* |
| *Nitrospirae* | 1 | *Thermodesulfovibrio* |
| *Proteobacteria* | 22 | *Pseudomonas, Xanthomonas, Thiobacillus, Helicobacter, Thiocapsa, Magnetospirillum, Comamonas, Thiomonas, Neisseria, Desulfovibrio, Desulfoglaeba, Thermodesulforhabdus, Microbulbifer, Idiomarina, Halochromatium, Halorhodospira, Thiohalospira, Methylothermus, Xylella, Marichromatium, Campylobacter, Candidatus* Liberibacter |
| *Spirochaetes* | 3 | *Borrelia, Leptonema, Leptospira* |
| *Tenericutes* | 1 | *Spiroplasma* |
| *Thermodesulfobacteria* | 2 | *Thermodesulfobacterium, Thermodesulfatator* |
| *Thermotogae* | 2 | *Thermotoga, Fervidobacterium* |
| *Verrucomicrobia* | 1 | *Chthoniobacter* |

**Supplementary Table 27.** Total number of genera identified in WG4 after searching both its metagenomic readsets against the RDP database.

| **Name of the phylum** | **Number of genera identified** | **Names of genera identified** |
| --- | --- | --- |
| *Actinobacteria* | 18 | *Corynebacterium, Glycomyces, Clavibacter, Actinoplanes, Nocardia, Rhodococcus, Propionibacterium, Thermobispora, Kitasatospora, Actinomadura, Tropheryma, Bifidobacterium, Atopobium, Eggerthella, Enterorhabdus, Thermoleophilum, Rathayibacter, Streptomyces* |
| *Aquificae* | 5 | *Hydrogenobacter, Desulfurobacterium, Persephonella, Sulfurihydrogenibium, Venenivibrio* |
| *Bacteroidetes* | 28 | *Bacteroides, Butyricimonas, Odoribacter, Parabacteroides, Porphyromonas, Tannerella, Prevotella, Alistipes, Rikenella, Cytophaga, Flexibacter, Hymenobacter, Microscilla, Flexithrix, Blattabacterium, Coenonia, Flavobacterium, Leeuwenhoekiella, Myroides, Ornithobacterium, Riemerella, Tenacibaculum, Zunongwangia, Rhodothermus, Salinibacter, Prolixibacter, Capnocytophaga, Maribacter* |
| *Chlamydiae* | 2 | *Criblamydia, Chlamydophila* |
| *Chlorobi* | 4 | *Chlorobaculum, Chlorobium, Chloroherpeton, Prosthecochloris* |
| *Chloroflexi* | 7 | *Chloroflexus, Roseiflexus, Oscillochloris, Herpetosiphon, Dehalococcoides, Sphaerobacter, Thermomicrobium* |
| *Cyanobacteria* | 21 | *Gloeobacter, Acaryochloris, Microcystis, Synechococcus, Thermosynechococcus, Anabaena, Anabaenopsis, Dolichospermum, Nodularia, Nostoc, Calothrix, Geitlerinema, Leptolyngbya, Lyngbya, Pseudanabaena, Spirulina, Chroococcidiopsis, Prochlorothrix, Arthrospira, Oscillatoria, Planktothrix* |
| *Deferribacteres* | 2 | *Deferribacter, Flexistipes* |
| *Deinococcus-Thermus* | 2 | *Meiothermus, Thermus* |
| *Dictyoglomi* | 1 | *Dictyoglomus* |
| *Firmicutes* | 22 | *Alicyclobacillus, Bacillus, Blautia, Brevibacillus, Caldicellulosiruptor, Caloramator, Clostridium, Desulfitobacterium, Geobacillus, Halothermothrix, Lactobacillus, Leuconostoc, Staphylococcus, Streptococcus, Sulfobacillus, Syntrophomonas, Veillonella, Weissella, Mechercharimyces, Paenibacillus, Sporanaerobacter, Ammonifex* |
| *Fusobacteria* | 1 | *Ilyobacter* |
| *Nitrospirae* | 2 | *Leptospirillum, Thermodesulfovibrio* |
| *Planctomycetes* | 1 | *Isosphaera* |
| *Proteobacteria* | 33 | *Candidatus* Liberibacter*, Acetobacter, Rubritepida, Rhodovibrio, Ehrlichia, Ralstonia, Nitrosospira, Desulfosarcina, Desulfomicrobium, Desulfovibrio, Desulfocaldus, Campylobacter, Helicobacter, Acidithiobacillus, Pseudoalteromonas, Marichromatium, Thiocapsa, Halorhodospira, Thioalkalivibrio, Methylohalobius, Methylothermus, Acinetobacter, Pseudomonas, Stenotrophomonas, Xanthomonas, Xylella, Francisella, Magnetospirillum, Burkholderia, Methylobacillus, Thauera, Desulfonauticus, Allochromatium* |
| *Spirochaetes* | 5 | *Leptonema, Leptospira, Borrelia, Spirochaeta, Treponema* |
| *Synergistetes* | 1 | *Aminobacterium* |
| *Tenericutes* | 1 | *Spiroplasma* |
| *Thermodesulfobacteria* | 2 | *Thermodesulfatator, Thermodesulfobacterium* |
| *Thermotogae* | 3 | *Fervidobacterium, Thermosipho, Thermotoga* |
| *Verrucomicrobia* | 1 | *Chthoniobacter* |

**Supplementary Table 28.** Total number of genera identified in WG5 after searching both its metagenomic readsets against the RDP database.

| **Name of the phylum** | **Number of genera identified** | **Names of genera identified** |
| --- | --- | --- |
| *Actinobacteria* | 4 | *Streptomyces, Bifidobacterium, Acidithiomicrobium, Rhodococcus* |
| *Bacteroidetes* | 17 | *Cytophaga, Flexibacter, Hymenobacter, Microscilla, Spirosoma, Flammeovirga, Flexithrix, Blattabacterium, Capnocytophaga, Flavobacterium, Wautersiella, Prolixibacter, Prevotella, Flectobacillus, Elizabethkingia, Saprospira, Chitinophaga* |
| *Chlorobi* | 4 | *Chlorobaculum, Chloroherpeton, Prosthecochloris, Chlorobium* |
| *Chloroflexi* | 7 | *Chloroflexus, Roseiflexus, Oscillochloris, Herpetosiphon, Dehalococcoides, Sphaerobacter, Thermomicrobium* |
| *Cyanobacteria* | 13 | *Microcystis, Synechococcus, Anabaena, Nostoc, Calothrix, Geitlerinema, Leptolyngbya, Oscillatoria, Pseudanabaena, Chroococcidiopsis, Stanieria, Xenococcus, Prochlorothrix* |
| *Firmicutes* | 10 | *Alicyclobacillus, Leuconostoc, Oxobacter, Tissierella, Butyrivibrio, Ammonifex, Caldanaerobius, Bacillus, Lactobacillus, Clostridium* |
| *Proteobacteria* | 10 | *Methylobacterium, Bdellovibrio, Desulfocella, Desulfomicrobium, Helicobacter, Thiocapsa, Pseudomonas, Acinetobacter, Thiothrix, Desulfovibrio* |
| *Spirochaetes* | 3 | *Leptospira, Spirochaeta, Treponema* |
| *Tenericutes* | 1 | *Candidatus* Phytoplasma |
| *Thermotogae* | 1 | *Thermotoga* |
| *Verrucomicrobia* | 2 | *Chthoniobacter, Methylacidiphilum* |

**Supplementary Table 29.** Total number of genera identified in WG6 after searching both its metagenomic readsets against the RDP database.

| **Name of the phylum** | **Number of genera identified** | **Names of genera identified** |
| --- | --- | --- |
| *Acidobacteria* | 1 | *Candidatus* Solibacter |
| *Actinobacteria* | 13 | *Actinomyces, Kytococcus, Gordonia, Arthrobacter, Nocardia, Rhodococcus, Streptomyces, Alloscardovia, Stackebrandtia, Microbacterium, Kocuria, Bifidobacterium, Atopobium* |
| *Aquificae* | 3 | *Hydrogenobaculum, Persephonella, Sulfurihydrogenibium* |
| *Bacteroidetes* | 45 | *Marinilabilia, Arenibacter, Butyricimonas, Candidatus* Amoebophilus*, Candidatus* Azobacteroides*, Candidatus* Hemobacterium*, Capnocytophaga, Cellulophaga Chitinophaga, Chryseobacterium, Coenonia, Cyclobacterium, Cytophaga, Dokdonia, Elizabethkingia, Flammeovirga, Flavobacterium, Flectobacillus, Flexibacter, Flexithrix, Gramella, Hymenobacter, Leeuwenhoekiella, Microscilla, Myroides, Odoribacter, Ornithobacterium, Parabacteroides, Pedobacter, Persicobacter, Polaribacter, Porphyromonas, Prevotella, Prolixibacter, Rhodothermus, Riemerella, Salinibacter, Saprospira, Sphingobacterium, Tenacibaculum, Terrimonas, Zunongwangia, Wautersiella, Bacteroides, Alistipes* |
| *Chlamydiae* | 4 | *Criblamydia, Candidatus* Protochlamydia*, Neochlamydia, Parachlamydia* |
| *Chlorobi* | 4 | *Chlorobaculum, Chlorobium, Chloroherpeton, Prosthecochloris* |
| *Chloroflexi* | 7 | *Chloroflexus, Roseiflexus, Oscillochloris, Herpetosiphon, Dehalococcoides, Sphaerobacter, Thermomicrobium* |
| *Cyanobacteria* | 16 | *Acaryochloris, Gloeocapsopsis, Microcystis, Synechococcus, Anabaena, Dolichospermum, Calothrix, Lyngbya, Oscillatoria, Pseudanabaena, Stanieria, Prochlorothrix, Xenococcus, Symploca, Planktothrix, Arthrospira* |
| *Deinococcus-Thermus* | 3 | *Deinococcus, Truepera, Thermus* |
| *Firmicutes* | 25 | *Alicyclobacillus, Ammonifex, Bacillus, Brevibacillus, Clostridium, Desulfosporosinus, Erysipelothrix, Jeotgalibacillus, Laceyella, Lactobacillus, Lactococcus, Leuconostoc, Lysinibacillus, Megasphaera, Pelotomaculum, Planomicrobium, Ruminococcus, Selenomonas, Sporosarcina, Staphylococcus, Aeribacillus, Carnobacterium, Desulfotomaculum, Geobacillus, Phascolarctobacterium* |
| *Planctomycetes* | 4 | *Blastopirellula, Isosphaera, Pirellula, Planctomyces* |
| *Proteobacteria* | 62 | *Asticcacaulis, Acetobacter, Acidiphilium, Acinetobacter, Alcaligenes, Anaplasma, Bdellovibrio, Blastomonas, Bradyrhizobium, Burkholderia, Candidatus* Neoehrlichia*, Desulfobacterium, Desulfocella, Desulfomicrobium, Desulfovibrio, Desulfuromonas, Erythrobacter, Escherichia, Geobacter, Gluconacetobacter, Gluconobacter, Helicobacter, Holospora, Hyphomonas, Jannaschia, Kinetoplastibacterium, Klebsiella, Legionella, Loktanella, Mesorhizobium, Methylobacterium, Methylophaga, Nitrosococcus, Nitrosomonas, Ochrobactrum, Paracoccus, Phaeobacter, Porphyrobacter, Pseudomonas, Pseudoruegeria, Rhizobium, Rhodobacter, Rhodobium, Rhodospirillum, Rhodothalassium, Rhodovibrio, Rhodovulum, Roseisalinus, Roseobacter, Roseococcus, Roseospira, Rubritepida, Sorangium, Sphingobium, Sphingomonas, Sphingopyxis, Stenotrophomonas, Thermodesulforhabdus, Thioclava, Thiothrix, Xanthomonas, Xylella* |
| *Spirochaetes* | 3 | *Leptospira, Borrelia, Treponema* |
| *Tenericutes* | 2 | *Acholeplasma, Candidatus* Phytoplasma |
| *Thermotogae* | 1 | *Thermotoga* |
| *Verrucomicrobia* | 7 | *Diplosphaera, Chthoniobacter, Pedosphaera, Akkermansia, Prosthecobacter, Rubritalea, Methylacidiphilum* |

**Supplementary Table 30.** Total number of genera identified in WG7 after searching both its metagenomic readsets against the RDP database.

| **Name of the phylum** | **Number of genera identified** | **Names of genera identified** |
| --- | --- | --- |
| *Euryarchaeota* | 1 | *Methanocella* |
| *Acidobacteria* | 1 | *Candidatus* Solibacter |
| *Actinobacteria* | 19 | *Acidithiomicrobium, Brevibacterium, Corynebacterium, Microbacterium, Arthrobacter, Nesterenkonia, Nocardia, Rhodococcus, Nocardioides, Thermobifida, Propionibacterium, Amycolatopsis, Kitasatospora, Streptomyces, Actinomadura, Atopobium, Eggerthella, Actinocorallia, Thermoleophilum* |
| *Aquificae* | 1 | *Sulfurihydrogenibium* |
| *Bacteroidetes* | 45 | *Bacteroides, Alistipes, Butyricimonas, Candidatus* Amoebophilus*, Candidatus* Azobacteroides*, Capnocytophaga, Cellulophaga, Chitinophaga, Coenonia, Cyclobacterium, Cytophaga, Dokdonia, Elizabethkingia, Flammeovirga, Flavobacterium, Flectobacillus, Flexibacter, Flexithrix, Gramella, Hymenobacter, Leeuwenhoekiella, Microscilla, Myroides, Odoribacter, Ornithobacterium, Parabacteroides, Pedobacter, Persicobacter, Porphyromonas, Prevotella, Prolixibacter, Rhodothermus, Riemerella, Rikenella, Salinibacter, Saprospira, Sphingobacterium, Tannerella, Tenacibaculum, Terrimonas, Wautersiella, Zobellia, Marinilabilia, Chryseobacterium, Barnesiella* |
| *Chlamydiae* | 5 | *Criblamydia, Candidatus* Protochlamydia*, Waddlia, Parachlamydia, Chlamydophila* |
| *Chlorobi* | 5 | *Chlorobaculum, Chlorobium, Chloroherpeton, Prosthecochloris, Pelodictyon* |
| *Chloroflexi* | 7 | *Chloroflexus, Roseiflexus, Oscillochloris, Herpetosiphon, Dehalococcoides, Sphaerobacter, Thermomicrobium* |
| *Cyanobacteria* | 32 | *Acaryochloris, Anabaena, Calothrix, Chlorogloeopsis, Chroococcidiopsis, Cuspidothrix, Cyanothece, Dolichospermum, Fischerella, Geitlerinema, Gloeocapsopsis, Leptolyngbya, Lyngbya, Microcystis, Nodularia, Nostoc, Oscillatoria, Phormidium, Planktothrix, Prochlorococcus, Prochlorothrix, Pseudanabaena, Spirulina, Stanieria, Symploca, Synechococcus, Thermosynechococcus, Xenococcus, Synechocystis, Scytonema, Anabaenopsis, Cyanobacterium* |
| *Deferribacteres* | 2 | *Denitrovibrio, Flexistipes* |
| *Deinococcus-Thermus* | 4 | *Deinococcus, Truepera, Meiothermus, Thermus* |
| *Firmicutes* | 39 | *Alicyclobacillus, Ammonifex, Bacillus, Butyricicoccus, Butyrivibrio, Caldicellulosiruptor, Candidatus Desulforudis, Clostridium, Desulfitibacter, Desulfitobacterium, Desulfonispora, Desulfotomaculum, Dialister, Faecalibacterium, Geobacillus, Halothermothrix, Kurthia, Laceyella, Lactobacillus, Leuconostoc, Phascolarctobacterium, Ruminococcus, Sporanaerobacter, Staphylococcus, Streptococcus, Sulfobacillus, Symbiobacterium, Syntrophomonas, Tissierella, Acetivibrio, Desulfosporosinus, Eubacterium, Lachnospira, Moorella, Oenococcus, Selenomonas, Tepidimicrobium, Thermodesulfobium, Veillonella* |
| *Fusobacteria* | 1 | *Fusobacterium* |
| *Nitrospirae* | 2 | *Leptospirillum, Nitrospira* |
| *Planctomycetes* | 6 | *Candidatus* Kuenenia*, Blastopirellula, Pirellula, Planctomyces, Rhodopirellula, Isosphaera* |
| *Proteobacteria* | 89 | *Hyphomicrobium, Acetobacter, Acidiphilium, Acidovorax, Afifella, Alcaligenes, Alkalispirillum, Aquabacterium, Azoarcus, Azospira, Azospirillum, Bdellovibrio, Bordetella, Burkholderia, Caedibacter, Campylobacter, Candidatus* Liberibacter*, Candidatus* Tremblaya*, Chondromyces, Chromobacterium, Corallococcus, Dechloromonas, Derxia, Desulfobacter, Desulfocaldus, Desulfocella, Desulfococcus, Desulfohalobium, Desulfomicrobium, Desulfomonile, Desulfonatronovibrio, Desulfonatronum, Desulfonema, Desulforhabdus, Desulfovibrio, Erythrobacter, Escherichia, Francisella, Gluconobacter, Helicobacter, Hydrogenimonas, Hyphomonas, Kinetoplastibacterium, Kozakia, Legionella, Magnetospirillum, Maricaulis, Marinosulfonomonas, Methylobacillus, Methylobacterium, Methylohalomonas, Methylosinus, Myxococcus, Nitrococcus, Nitrosomonas, Nitrosospira, Nitrosovibrio, Novosphingobium, Oxalobacter, Pannonibacter, Paracoccus, Pasteurella, Phaeospirillum, Plesiocystis, Porphyrobacter, Pseudomonas, Ralstonia, Rhodobaca, Rhodobacter, Rhodovulum, Rickettsia, Rubritepida, Rubrivivax, Sorangium, Sphingobium, Sphingopyxis, Stenotrophomonas, Syntrophobacter, Thermodesulforhabdus, Thioalkalivibrio, Thiobacter, Thiocapsa, Thiococcus, Thiorhodococcus, Thiorhodospira, Thiothrix, Wolbachia, Xanthomonas, Xylella* |
| *Spirochaetes* | 4 | *Leptospira, Borrelia, Spirochaeta, Treponema* |
| *Tenericutes* | 4 | *Acholeplasma, Spiroplasma, Mycoplasma, Candidatus* Phytoplasma |
| *Thermotogae* | 3 | *Petrotoga, Thermosipho, Thermotoga* |
| *Verrucomicrobia* | 8 | *Opitutus, Chthoniobacter, Pedosphaera, Akkermansia, Prosthecobacter, Rubritalea, Methylacidiphilum, Verrucomicrobium* |

**Supplementary Table 31.** Microbial genera that were detected^1^ in all the three mat communities of the progressively-drying thermal gradient.

| **Phylum affiliation** | **VWM** | **DG3** | **DG4** |
| --- | --- | --- | --- |
| ***Actinobacteria*** | *Thermobispora* (0.009%)  *Thermobifida* (0.01%)  *Tropheryma* (0.001%)  *Saccharopolyspora* (0.01%) | *Thermobispora* (0.02%)  *Thermobifida* (0.04%)  *Tropheryma* (0.004%)  *Saccharopolyspora* (0.04%) | *Thermobispora* (0.07%)  *Thermobifida* (0.09%)  *Tropheryma* (0.01%)  *Saccharopolyspora* (0.09%) |
| ***Aquificae*** | *Sulfurihydrogenibium* (21.57%)  *Persephonella* (0.27%) | *Sulfurihydrogenibium* (0.12%)  *Persephonella* (0.02%) | *Sulfurihydrogenibium* (0.06%)  *Persephonella* (0.02%) |
| ***Bacteroidetes*** | *Alistipes* (0.009%)  *Cytophaga* (0.02%) | *Alistipes* (0.03%)  *Cytophaga* (0.25%) | *Alistipes* (0.03%)  *Cytophaga* (0.25%) |
| ***Chloroflexi*** | *Chloroflexus* (0.4%)  *Roseiflexus* (0.89%)  *Herpetosiphon* (0.02%)  *Dehalococcoides* (0.05%)  *Thermomicrobium* (0.02%) | *Chloroflexus* (4.56%)  *Roseiflexus* (14.73%)  *Herpetosiphon* (0.96%)  *Dehalococcoides* (0.1%)  *Thermomicrobium* (0.13%) | *Chloroflexus* (7.22%)  *Roseiflexus* (13.62%)  *Herpetosiphon* (2.1%)  *Dehalococcoides* (0.28%)  *Thermomicrobium* (0.35%) |
| ***Cyanobacteria*** | *Synechococcus* (0.17%)  *Oscillatoria* (0.01%) | *Synechococcus* (3.09%)  *Oscillatoria* (0.48%) | *Synechococcus* (1.08%)  *Oscillatoria* (0.18%) |
| ***Deinococcus-Thermus*** | *Thermus* (50.64%) | *Thermus* (1.03%) | *Thermus* (0.44%) |
| ***Firmicutes*** | *Bacillus* (0.18%)  *Clostridium* (0.27%)  *Streptococcus* (0.03%) | *Bacillus* (0.525%)  *Clostridium* (0.32%)  *Streptococcus* (0.06%) | *Bacillus* (0.88%)  *Clostridium* (0.56%)  *Streptococcus* (0.11%) |
| ***Proteobacteria*** | *Pseudomonas* (0.07%)  *Thiobacillus* (0.02%)  *Acidithiobacillus* (0.01%)  *Campylobacter* (0.08%) | *Pseudomonas* (0.215%)  *Thiobacillus* (0.04%)  *Acidithiobacillus* (0.03%)  *Campylobacter* (0.04%) | *Pseudomonas* (1.36%)  *Thiobacillus* (0.05%)  *Acidithiobacillus* (0.05%)  *Campylobacter* (0.05%) |
| ***Spirochaetes*** | *Treponema* (0.02%) | *Treponema* (0.1%) | *Treponema* (0.1%) |
| ***Thermotogae*** | *Thermotoga* (0.8%)  *Fervidobacterium* (0.16%) | *Thermotoga* (0.09%)  *Fervidobacterium* (0.03%) | *Thermotoga* (0.14%)  *Fervidobacterium* (0.04%) |

**^1^** Genera which were identified upon searching the respective metagenomes against the RDP as well as the *nr* protein database have their names underlined; those identified only upon searching against the *nr* protein database (and were not detectable in the RDP search) are not underlined. Mean percentage of all annotable metagenomic reads of the community ascribable to a genus (upon searching against the *nr* protein database) is given in parenthesis after the name of the genus.

**Supplementary Table 32.** Microbial genera that were detected^1^ in all the six mat communities of the wet thermal gradient.

|  | **VWM** | **WG3** | **WG4** | **WG5** | **WG6** | **WG7** |
| --- | --- | --- | --- | --- | --- | --- |
| ***Actinobacteria*** | *Kitasatospora* (0.008%)  *Thermobispora* (0.009%)  *Thermobifida* (0.01%)  *Streptomyces* (0.03%)  *Tropheryma* (0.001%)  *Propionibacterium* (0.01%)  *Saccharopolyspora* (0.01%) | *Kitasatospora* (0.009%)  *Thermobispora* (0.01%)  *Thermobifida* (0.02%)  *Streptomyces* (0.13%)  *Tropheryma* (0.004%)  *Propionibacterium* (0.02%)  *Saccharopolyspora* (0.03%) | *Kitasatospora* (0.008%)  *Thermobispora* (0.02%)  *Thermobifida* (0.03%)  *Streptomyces* (0.16%)  *Tropheryma* (0.003%)  *Propionibacterium* (0.03%)  *Saccharopolyspora* (0.03%) | *Kitasatospora* (0.01%)  *Thermobispora* (0.03%)  *Thermobifida* (0.06%)  *Streptomyces* (0.27%)  *Tropheryma* (0.002%)  *Propionibacterium* (0.03%)  *Saccharopolyspora* (0.05%) | *Kitasatospora* (0.01%)  *Thermobispora* (0.02%)  *Thermobifida* (0.04%)  *Streptomyces* (0.21%)  *Tropheryma* (0.004%)  *Propionibacterium* (0.06%)  *Saccharopolyspora* (0.05%) | *Kitasatospora* (0.01%)  *Thermobispora* (0.03%)  *Thermobifida* (0.04%)  *Streptomyces* (0.28%)  *Tropheryma* (0.004%)  *Propionibacterium* (0.06%)  *Saccharopolyspora* (0.05%) |
| ***Aquificae*** | *Sulfurihydrogenibium* (21.57%)  *Persephonella* (0.27%)  *Hydrogenobaculum* (0.28%) | *Sulfurihydrogenibium* (7.31%)  *Persephonella* (0.1%)  *Hydrogenobaculum* (0.09%) | *Sulfurihydrogenibium* (6.33%)  *Persephonella* (0.1%)  *Hydrogenobaculum* (0.1%) | *Sulfurihydrogenibium* (0.04%)  *Persephonella* (0.01%)  *Hydrogenobaculum* (0.01%) | *Sulfurihydrogenibium* (0.04%)  *Persephonella* (0.02%)  *Hydrogenobaculum* (0.01%) | *Sulfurihydrogenibium* (0.08%)  *Persephonella* (0.03%)  *Hydrogenobaculum* (0.02%) |
| ***Bacteroidetes*** | *Alistipes* (0.009%)  *Cytophaga* (0.02%)  *Rhodothermus* (0.08%)  *Salinibacter* (0.05%) | *Alistipes* (0.04%)  *Cytophaga* (0.13%)  *Rhodothermus* (0.29%)  *Salinibacter* (0.17%) | *Alistipes* (0.09%)  *Cytophaga* (0.26%)  *Rhodothermus* (0.36%)  *Salinibacter* (0.24%) | *Alistipes* (0.03%)  *Cytophaga* (0.2%)  *Rhodothermus* (0.19%)  *Salinibacter* (0.17%) | *Alistipes* (0.22%)  *Cytophaga* (1.38%)  *Rhodothermus* (0.33%)  *Salinibacter* (0.26%) | *Alistipes* (0.21%)  *Cytophaga* (0.77%)  *Rhodothermus* (0.43%)  *Salinibacter* (0.34%) |
| ***Chloroflexi*** | *Chloroflexus* (0.4%)  *Roseiflexus* (0.89%)  *Herpetosiphon* (0.02%)  *Dehalococcoides* (0.05%)  *Thermomicrobium* (0.02%) | *Chloroflexus* (3.09%)  *Roseiflexus* (20.39%)  *Herpetosiphon* (0.34%)  *Dehalococcoides* (0.08%)  *Thermomicrobium* (0.1%) | *Chloroflexus* (3.5%)  *Roseiflexus* (12.7%)  *Herpetosiphon* (0.4%)  *Dehalococcoides* (0.1%)  *Thermomicrobium* (0.11%) | *Chloroflexus* (6.97%)  *Roseiflexus* (9.86%)  *Herpetosiphon* (1.66%)  *Dehalococcoides* (0.13%)  *Thermomicrobium* (0.17%) | *Chloroflexus* (1.87%)  *Roseiflexus* (2.53%)  *Herpetosiphon* (0.57%)  *Dehalococcoides* (0.08%)  *Thermomicrobium* (0.09%) | *Chloroflexus* (1.26%)  *Roseiflexus* (2.12%)  *Herpetosiphon* (0.48%)  *Dehalococcoides* (0.11%)  *Thermomicrobium* (0.1%) |
| ***Cyanobacteria*** | *Synechococcus* (0.17%)  *Oscillatoria* (0.01%) | *Synechococcus* (5.09%)  *Oscillatoria* (0.07%) | *Synechococcus* (8%)  *Oscillatoria* (0.12%) | *Synechococcus* (4.74%)  *Oscillatoria* (0.82%) | *Synechococcus* (0.48%)  *Oscillatoria* (0.06%) | *Synechococcus* (1.98%)  *Oscillatoria* (0.32%) |
| ***Deinococcus-***  ***Thermus*** | *Thermus* (50.64%)  *Meiothermus* (2.16%)  *Deinococcus* (0.34%) | *Thermus* (7.8%)  *Meiothermus* (0.57%)  *Deinococcus* (0.17%) | *Thermus* (5.38%)  *Meiothermus* (0.44%)  *Deinococcus* (0.18%) | *Thermus* (0.23%)  *Meiothermus* (0.24%)  *Deinococcus* (0.26%) | *Thermus* (0.11%)  *Meiothermus* (0.13%)  *Deinococcus* (0.16%) | *Thermus* (0.17%)  *Meiothermus* (0.17%)  *Deinococcus* (0.2%) |
| ***Firmicutes*** | *Anoxybacillus* (0.01%)  *Alicyclobacillus* (0.03%)  *Bacillus* (0.18%)  *Geobacillus* (0.06%)  *Paenibacillus* (0.03%)  *Lactobacillus* (0.05%)  *Clostridium* (0.27%)  *Leuconostoc* (0.01%)  *Streptococcus* (0.03%)  *Coprothermobacter* (0.01%) | *Anoxybacillus* (0.02%)  *Alicyclobacillus* (0.04%)  *Bacillus* (0.31%)  *Geobacillus* (0.13%)  *Paenibacillus* (0.07%)  *Lactobacillus* (0.06%)  *Clostridium* (0.23%)  *Leuconostoc* (0.01%)  *Streptococcus* (0.05%)  *Coprothermobacter* (0.02%) | *Anoxybacillus* (0.03%)  *Alicyclobacillus* (0.05%)  *Bacillus* (0.41%)  *Geobacillus* (0.16%)  *Paenibacillus* (0.11%)  *Lactobacillus* (0.08%)  *Clostridium* (0.47%)  *Leuconostoc* (0.01%)  *Streptococcus* (0.06%)  *Coprothermobacter* (0.02%) | *Anoxybacillus* (0.03%)  *Alicyclobacillus* (0.06%)  *Bacillus* (0.5%)  *Geobacillus* (0.19%)  *Paenibacillus* (0.14%)  *Lactobacillus* (0.08%)  *Clostridium* (0.33%)  *Leuconostoc* (0.01%)  *Streptococcus* (0.07%)  *Coprothermobacter* (0.01%) | *Anoxybacillus* (0.03%)  *Alicyclobacillus* (0.04%)  *Bacillus* (0.69%)  *Geobacillus* (0.18%)  *Paenibacillus* (0.15%)  Lactobacillus (0.09%)  *Clostridium* (0.36%)  *Leuconostoc* (0.01%)  *Streptococcus* (0.08%)  *Coprothermobacter* (0.01%) | *Anoxybacillus* (0.04%)  *Alicyclobacillus* (0.07%)  *Bacillus* (0.61%)  *Geobacillus* (0.2%)  *Paenibacillus* (0.2%)  *Lactobacillus* (0.1%)  *Clostridium* (0.88%)  *Leuconostoc* (0.01%)  *Streptococcus* (0.09%)  *Coprothermobacter* (0.02%) |
| ***Proteobacteria*** | *Pseudomonas* (0.07%)  *Thiobacillus* (0.02%)  *Acidithiobacillus* (0.01%)  *Campylobacter* (0.08%) | *Pseudomonas* (0.67%)  *Thiobacillus* (0.43%)  *Acidithiobacillus* (0.03%)  *Campylobacter* (0.06%) | *Pseudomonas* (0.19%)  *Thiobacillus* (0.05%)  *Acidithiobacillus* (0.03%)  *Campylobacter* (0.07%) | *Pseudomonas* (0.3%)  *Thiobacillus* (0.04%)  *Acidithiobacillus* (0.04%)  *Campylobacter* (0.04%) | *Pseudomonas* (0.35%)  *Thiobacillus* (0.08%)  *Acidithiobacillus* (0.04%)  *Campylobacter* (0.08%) | *Pseudomonas* (0.53%)  *Thiobacillus* (0.25%)  *Acidithiobacillus* (0.08%)  *Campylobacter* (0.07%) |
| ***Spirochaetes*** | *Treponema* (0.02%)  *Borrelia* (0.01%) | *Treponema* (0.06%)  *Borrelia* (0.01%) | *Treponema* (0.08%)  *Borrelia* (0.03%) | *Treponema* (0.14%)  *Borrelia* (0.04%) | *Treponema* (0.05%)  *Borrelia* (0.02%) | *Treponema* (0.4%)  *Borrelia* (0.05%) |
| ***Thermotogae*** | *Thermotoga* (0.8%)  *Thermosipho* (0.12%)  *Fervidobacterium* (0.16%)  *Petrotoga* (0.08%) | *Thermotoga* (0.23%)  *Thermosipho* (0.06%)  *Fervidobacterium* (0.07%)  *Petrotoga* (0.05%) | *Thermotoga* (0.84%)  *Thermosipho* (0.14%)  *Fervidobacterium* (0.29%)  *Petrotoga* (0.07%) | *Thermotoga* (0.08%)  *Thermosipho* (0.03%)  *Fervidobacterium* (0.02%)  *Petrotoga* (0.02%) | *Thermotoga* (0.06%)  *Thermosipho* (0.03%)  *Fervidobacterium* (0.02%)  *Petrotoga* (0.02%) | *Thermotoga* (0.13%)  *Thermosipho* (0.06%)  *Fervidobacterium* (0.05%)  *Petrotoga* (0.04%) |

**^1^** Genera identified upon searching the metagenomes against RDP as well as nr protein database have their names underlined; those identified only upon searching against the nr protein database (and were not detectable in the RDP search) are not underlined. Mean percentage of all annotable metagenomic reads of the community ascribable to a genus (upon searching against nr protein database) is given in parenthesis after the genus name.

**Supplementary Table 33.** Microbial genera* comprised primarily of thermophilic species but present even at low-temperature (33-46°C) sites of *Shivlinga*’s thermal gradients.

| **Phyla** | **Genera** | **Temp. range for laboratory growth** | **References^#^** | **VWM**  **(66°C)** | **DG3**  **(52°C)** | **DG4**  **(41°C)** | **WG3**  **(56°C)** | **WG4**  **(46°C)** | **WG5**  **(38°C)** | **WG6**  **(36°C)** | **WG7**  **(33°C)** |
| --- | --- | --- | --- | --- | --- | --- | --- | --- | --- | --- | --- |
| ***Aquificae*** | ***Sulfurihydrogenibium*** | 70–40°C | ([Takai *et al*., 2003](#_ENREF_23)) | + | + | + | + | + | – | + | + |
|  | ***Persephonella*** | 80–55°C | ([Gotz *et al*., 2002](#_ENREF_7); [Nakagawa *et al*., 2003](#_ENREF_18)) | + | – | + | + | + | – | + | – |
|  | ***Hydrogenobaculum*** | 80–45°C | ([Stohr *et al*., 2001](#_ENREF_22)) | + | – | – | – | – | – | + | – |
| ***Deinococcus–Thermus*** | ***Thermus*** | 80–40°C | ([Vajna *et al*., 2012](#_ENREF_25)) | + | + | + | + | + | – | + | + |
|  | ***Meiothermus*** | 70–35°C | ([Chen *et al*., 2002](#_ENREF_4); [Mori *et al*., 2012](#_ENREF_15)) | + | – | – | + | + | – | – | + |
| ***Dictyoglomi*** | ***Dictyoglomus*** | 80–50°C | ([Saiki *et al*., 1985](#_ENREF_21)) | + | – | – | – | + | – | – | – |
| ***Thermodesulfobacteria*** | ***Thermodesulfobacterium*** | 80–50°C | ([Jeanthon *et al*., 2002](#_ENREF_11)) | + | – | + | + | + | – | – | – |
|  | ***Thermodesulfatator*** | 80–55°C | ([Alain *et al*., 2010](#_ENREF_1); [Moussard *et al*., 2004](#_ENREF_16)) | + | – | – | + | + | – | – | – |
| ***Thermotogae*** | ***Thermotoga*** | 90–55 °C | ([Huber *et al*., 1986](#_ENREF_10)) | + | + | – | + | + | + | + | + |
|  | ***Thermosipho*** | 80–37°C | ([Podosokorskaya *et al*., 2011](#_ENREF_20); [Urios *et al*., 2004](#_ENREF_24)) | + | – | – | – | + | – | – | + |
|  | ***Petrotoga*** | 65–40°C | ([Lien *et al*., 1998](#_ENREF_14)) | – | – | – | – | – | – | – | + |

* Identified in RDP searches of the metagenomes.

^#^ References used in this table have been given in Supplementary references.

|  | **VWM**  **1^st^ sample** | **VWM**  **2^nd^ sample** | **VWM average** | **DG3**  **1^st^ sample** | **DG3**  **2^nd^ sample** | **DG3 average** | **DG4**  **1^st^ sample** | **DG4**  **2^nd^ sample** | **DG4 average** |
| --- | --- | --- | --- | --- | --- | --- | --- | --- | --- |
| ***Acidobacteria*** | 0.12 | 0.17 | 0.145 | 0.84 | 0.68 | 0.76 | 1.16 | 1.16 | 1.16 |
| ***Actinobacteria*** | 0.36 | 0.42 | 0.39 | 1.9 | 2.41 | 2.155 | 5.03 | 5.02 | 5.025 |
| ***Alphaproteobacteria*** | 0.46 | 0.62 | 0.54 | 2.3 | 2.82 | 2.56 | 7.26 | 7.58 | 7.42 |
| ***Aquificae*** | 21.7 | 33.3 | 27.5 | 0.48 | 0.2 | 0.34 | 0.31 | 0.29 | 0.3 |
| ***Bacteroidetes*** | 0.3 | 0.49 | 0.395 | 5.92 | 3.61 | 4.765 | 4.16 | 4.26 | 4.21 |
| ***Betaproteobacteria*** | 0.38 | 0.52 | 0.45 | 1.56 | 1.82 | 1.69 | 2.9 | 2.71 | 2.805 |
| ***Chlamydiae*** | 0.02 | 0.03 | 0.025 | 0.07 | 0.08 | 0.075 | 0.12 | 0.13 | 0.125 |
| ***Chlorobi*** | 0.21 | 0.36 | 0.285 | 16.44 | 3.42 | 9.93 | 4.13 | 4.01 | 4.07 |
| ***Chloroflexi*** | 1.96 | 1.61 | 1.785 | 34.72 | 30.97 | 32.845 | 40.93 | 40.8 | 40.865 |
| ***Cyanobacteria*** | 0.93 | 0.9 | 0.915 | 18.96 | 38.5 | 28.73 | 7.29 | 8.19 | 7.74 |
| ***Deferribacteres*** | 0.08 | 0.18 | 0.13 | 0.1 | 0.07 | 0.085 | 0.11 | 0.1 | 0.105 |
| ***Deinococcus-Thermus*** | 67.4 | 51.53 | 59.465 | 3.34 | 1.1 | 2.22 | 2.61 | 2.57 | 2.59 |
| ***Deltaproteobacteria*** | 1.27 | 2.48 | 1.875 | 2.54 | 2.72 | 2.63 | 3.99 | 3.97 | 3.98 |
| ***Dictyoglomi*** | 0.07 | 0.15 | 0.11 | 0.07 | 0.05 | 0.06 | 0.1 | 0.11 | 0.105 |
| ***Epsilonproteobacteria*** | 0.48 | 1.00 | 0.74 | 0.2 | 0.22 | 0.21 | 0.24 | 0.25 | 0.245 |
| ***Firmicutes*** | 1.7 | 2.74 | 2.22 | 4.9 | 4.48 | 4.69 | 8.23 | 8.37 | 8.3 |
| ***Fusobacteria*** | 0.04 | 0.08 | 0.06 | 0.1 | 0.09 | 0.095 | 0.14 | 0.15 | 0.145 |
| ***Gammaproteobacteria*** | 0.74 | 1.03 | 0.885 | 2.39 | 3.25 | 2.82 | 7.18 | 6.23 | 6.705 |
| ***Gemmatimonadetes*** | 0.01 | 0.01 | 0.01 | 0.11 | 0.07 | 0.09 | 0.12 | 0.11 | 0.115 |
| ***Nitrospirae*** | 0.18 | 0.4 | 0.29 | 0.16 | 0.12 | 0.14 | 0.18 | 0.17 | 0.175 |
| ***Planctomycetes*** | 0.06 | 0.09 | 0.075 | 0.47 | 0.56 | 0.515 | 0.9 | 0.92 | 0.91 |
| ***Spirochaetes*** | 0.07 | 0.11 | 0.09 | 0.42 | 1.06 | 0.74 | 0.54 | 0.53 | 0.535 |
| ***Synergistetes*** | 0.05 | 0.07 | 0.06 | 0.13 | 0.1 | 0.115 | 0.18 | 0.18 | 0.18 |
| ***Tenericutes*** | 0.03 | 0.03 | 0.03 | 0.04 | 0.03 | 0.035 | 0.06 | 0.07 | 0.065 |
| ***Thermotogae*** | 1.17 | 1.45 | 1.31 | 0.27 | 0.21 | 0.24 | 0.39 | 0.4 | 0.395 |
| ***Verrucomicrobia*** | 0.07 | 0.09 | 0.08 | 1.06 | 0.75 | 0.91 | 0.69 | 0.68 | 0.69 |
| **Unclassified *Bacteria*** | 0.08 | 0.1 | 0.09 | 0.36 | 0.38 | 0.37 | 0.81 | 0.81 | 0.81 |
| **Unclassified *Proteobacteria*** | 0.03 | 0.05 | 0.04 | 0.04 | 0.09 | 0.07 | 0.1 | 0.1 | 0.1 |
| **Others^2^, as shown in Fig.5A** | | | | | | | | | |
| ***Thermodesulfobacteria*** | 0.01 | 0.01 | 0.01 | 0 | 0 | 0 | 0 | 0 | 0 |
| ***Elusimicrobia*** | 0.01 | 0.01 | 0.01 | 0.02 | 0.02 | 0.02 | 0.02 | 0.02 | 0.02 |
| ***Chrysiogenetes*** | 0.01 | 0.01 | 0.01 | 0.02 | 0.02 | 0.02 | 0.03 | 0.03 | 0.03 |
| ***Lentisphaerae*** | 0 | 0.01 | 0.005 | 0.03 | 0.03 | 0.03 | 0.04 | 0.04 | 0.04 |
| ***Fibrobacteres*** | 0 | 0.02 | 0.01 | 0.02 | 0.02 | 0.02 | 0.02 | 0.02 | 0.02 |
| ***Poribacteria*** | 0 | 0 | 0 | 0.01 | 0.01 | 0.01 | 0.02 | 0.02 | 0.02 |
| ***Zetaproteobacteria*** | 0 | 0.01 | 0.005 | 0.01 | 0.01 | 0.01 | 0.02 | 0.02 | 0.02 |

**Supplementary Table 34.** Phylum- or class-level percentage distribution of the total bacterial metagenomic reads^1^ identified in the mat samples of the drying thermal gradient.

^1^ Reads were searched by BlastX against the *nr* protein database with minimum alignment length of 45 bp (15 amino acids) and minimum identity cutoff of 60%.

^2^ Other include bacterial phyla or proteobacterial classes contributing <0.1% reads in all the six metagenomic datasets.

**Supplementary Table 35.** Phylum- or class-level percentage distribution of the total bacterial metagenomic reads^1^ identified in the mat samples of the wet thermal gradient.

|  | **WG3**  **1^st^**  **sample** | **WG3**  **2^nd^**  **sample** | **WG3**  **Avg.** | **WG4**  **1^st^**  **sample** | **WG4**  **2^nd^**  **sample** | **WG4**  **Avg.** | **WG5**  **1^st^**  **sample** | **WG5**  **2^nd^**  **sample** | **WG5**  **Avg.** | **WG6**  **1^st^**  **sample** | **WG6**  **2^nd^**  **sample** | **WG6**  **Avg.** | **WG7**  **1^st^**  **sample** | **WG7**  **2^nd^**  **sample** | **WG7**  **Avg.** |
| --- | --- | --- | --- | --- | --- | --- | --- | --- | --- | --- | --- | --- | --- | --- | --- |
| ***Acidobacteria*** | 0.61 | 0.74 | 0.675 | 0.68 | 0.71 | 0.695 | 0.77 | 0.7 | 0.74 | 0.82 | 0.9 | 0.86 | 2.98 | 2.49 | 2.74 |
| ***Actinobacteria*** | 1.31 | 1.46 | 1.385 | 1.74 | 1.63 | 1.685 | 2.7 | 2.64 | 2.67 | 2.09 | 2.3 | 2.195 | 2.76 | 2.46 | 2.61 |
| ***Alphaproteobacteria*** | 1.53 | 1.75 | 1.64 | 1.77 | 1.79 | 1.78 | 3 | 2.9 | 2.95 | 8.55 | 10.87 | 9.71 | 11.89 | 8.8 | 10.35 |
| ***Aquificae*** | 9.54 | 13.35 | 11.445 | 8.95 | 10.48 | 9.715 | 0.2 | 0.19 | 0.2 | 0.2 | 0.19 | 0.2 | 0.31 | 0.35 | 0.33 |
| ***Bacteroidetes*** | 2.68 | 3.73 | 3.205 | 4.87 | 6.22 | 5.545 | 3 | 3.65 | 3.33 | 52.3 | 47.66 | 49.98 | 14.97 | 18.39 | 16.68 |
| ***Betaproteobacteria*** | 2.31 | 2.46 | 2.385 | 1.47 | 1.45 | 1.46 | 1.9 | 1.83 | 1.87 | 3.19 | 3.6 | 3.4 | 10.06 | 6.89 | 8.48 |
| ***Chlamydiae*** | 0.06 | 0.08 | 0.07 | 0.08 | 0.09 | 0.085 | 0.09 | 0.08 | 0.09 | 0.13 | 0.14 | 0.14 | 0.16 | 0.18 | 0.17 |
| ***Chlorobi*** | 3.36 | 4.12 | 3.74 | 7.57 | 8.82 | 8.195 | 2.99 | 3.5 | 3.25 | 2.41 | 2.41 | 2.41 | 6.44 | 7.7 | 7.07 |
| ***Chloroflexi*** | 39.06 | 33.38 | 36.22 | 30.19 | 22.5 | 26.345 | 34.28 | 31.51 | 32.9 | 8.98 | 8.92 | 8.95 | 8.07 | 6.78 | 7.43 |
| ***Cyanobacteria*** | 13.27 | 12.05 | 12.66 | 18.84 | 19.71 | 19.275 | 35.02 | 37.09 | 36.06 | 3.17 | 3.58 | 3.38 | 13.4 | 17.73 | 15.57 |
| ***Deferribacteres*** | 0.11 | 0.17 | 0.14 | 0.27 | 0.43 | 0.35 | 0.07 | 0.07 | 0.07 | 0.1 | 0.1 | 0.1 | 0.16 | 0.17 | 0.17 |
| ***Deinococcus-Thermus*** | 13.01 | 10.64 | 11.825 | 7.94 | 8.05 | 7.995 | 1.28 | 1.25 | 1.27 | 0.7 | 0.7 | 0.7 | 0.97 | 0.87 | 0.92 |
| ***Deltaproteobacteria*** | 2.36 | 3.08 | 2.72 | 2.97 | 3.53 | 3.25 | 2.86 | 2.83 | 2.85 | 2.69 | 2.84 | 2.77 | 6.54 | 6.13 | 6.34 |
| ***Dictyoglomi*** | 0.07 | 0.1 | 0.085 | 0.22 | 0.35 | 0.285 | 0.06 | 0.06 | 0.06 | 0.05 | 0.04 | 0.05 | 0.08 | 0.09 | 0.09 |
| ***Epsilonproteobacteria*** | 0.38 | 0.55 | 0.465 | 0.42 | 0.57 | 0.495 | 0.2 | 0.2 | 0.2 | 0.39 | 0.41 | 0.4 | 0.36 | 0.41 | 0.39 |
| ***Firmicutes*** | 3.34 | 4.34 | 3.84 | 4.64 | 5.34 | 4.99 | 4.88 | 4.58 | 4.73 | 4.54 | 4.64 | 4.59 | 6.46 | 6.87 | 6.67 |
| ***Fusobacteria*** | 0.09 | 0.11 | 0.1 | 0.11 | 0.17 | 0.14 | 0.08 | 0.09 | 0.09 | 0.16 | 0.16 | 0.16 | 0.18 | 0.21 | 0.2 |
| ***Gammaproteobacteria*** | 3.77 | 4.04 | 3.905 | 2.85 | 2.83 | 2.84 | 3.22 | 3.28 | 3.25 | 5.33 | 5.73 | 5.53 | 7.04 | 6.52 | 6.78 |
| ***Gemmatimonadetes*** | 0.07 | 0.07 | 0.07 | 0.08 | 0.08 | 0.08 | 0.08 | 0.06 | 0.07 | 0.09 | 0.08 | 0.09 | 0.18 | 0.16 | 0.17 |
| ***Nitrospirae*** | 0.17 | 0.24 | 0.205 | 0.37 | 0.58 | 0.475 | 0.14 | 0.12 | 0.13 | 0.11 | 0.13 | 0.12 | 0.22 | 0.22 | 0.22 |
| ***Planctomycetes*** | 0.34 | 0.42 | 0.38 | 0.39 | 0.41 | 0.4 | 0.56 | 0.56 | 0.56 | 1.4 | 1.67 | 1.54 | 2.03 | 1.82 | 1.93 |
| ***Spirochaetes*** | 0.34 | 0.45 | 0.395 | 0.54 | 0.76 | 0.65 | 0.93 | 1.09 | 1.01 | 0.39 | 0.4 | 0.4 | 1.59 | 1.69 | 1.64 |
| ***Synergistetes*** | 0.1 | 0.12 | 0.11 | 0.14 | 0.17 | 0.155 | 0.11 | 0.1 | 0.105 | 0.09 | 0.1 | 0.1 | 0.23 | 0.22 | 0.23 |
| ***Tenericutes*** | 0.03 | 0.05 | 0.04 | 0.05 | 0.06 | 0.055 | 0.04 | 0.03 | 0.035 | 0.06 | 0.06 | 0.06 | 0.05 | 0.06 | 0.06 |
| ***Thermotogae*** | 0.48 | 0.66 | 0.57 | 1.58 | 2 | 1.79 | 0.22 | 0.23 | 0.225 | 0.18 | 0.18 | 0.18 | 0.35 | 0.38 | 0.37 |
| ***Verrucomicrobia*** | 1.16 | 1.3 | 1.23 | 0.72 | 0.68 | 0.7 | 0.7 | 0.75 | 0.725 | 1.14 | 1.42 | 1.28 | 1.54 | 1.5 | 1.52 |
| ***unclassified Bacteria*** | 0.28 | 0.33 | 0.305 | 0.35 | 0.35 | 0.35 | 0.43 | 0.4 | 0.415 | 0.47 | 0.47 | 0.47 | 0.55 | 0.51 | 0.53 |
| ***unclassified Proteobacteria*** | 0.05 | 0.06 | 0.055 | 0.05 | 0.06 | 0.055 | 0.09 | 0.09 | 0.09 | 0.07 | 0.07 | 0.07 | 0.13 | 0.12 | 0.125 |
| **Others^2^, as shown in Fig.5B** | | | | | | | | | | | | | | | |
| ***Thermodesulfobacteria*** | 0 | 0 | 0 | 0 | 0 | 0 | 0 | 0 | 0 | 0 | 0 | 0 | 0 | 0 | 0 |
| ***Elusimicrobia*** | 0.02 | 0.03 | 0.025 | 0.04 | 0.06 | 0.05 | 0.02 | 0.01 | 0.015 | 0.02 | 0.03 | 0.025 | 0.04 | 0.04 | 0.04 |
| ***Chrysiogenetes*** | 0.02 | 0.03 | 0.025 | 0.03 | 0.03 | 0.03 | 0.02 | 0.02 | 0.02 | 0.02 | 0.02 | 0.02 | 0.05 | 0.05 | 0.05 |
| ***Lentisphaerae*** | 0.02 | 0.03 | 0.025 | 0.03 | 0.03 | 0.03 | 0.04 | 0.03 | 0.035 | 0.08 | 0.09 | 0.085 | 0.11 | 0.1 | 0.105 |
| ***Zetaproteobacteria*** | 0.01 | 0.02 | 0.015 | 0.01 | 0.02 | 0.015 | 0.02 | 0.01 | 0.015 | 0.02 | 0.02 | 0.02 | 0.04 | 0.04 | 0.04 |
| ***Fibrobacteres*** | 0.01 | 0.02 | 0.015 | 0.02 | 0.02 | 0.02 | 0.02 | 0.02 | 0.02 | 0.03 | 0.02 | 0.025 | 0.04 | 0.04 | 0.04 |
| ***Poribacteria*** | 0.01 | 0.01 | 0.01 | 0.01 | 0.01 | 0.01 | 0.02 | 0.02 | 0.02 | 0.02 | 0.02 | 0.02 | 0.02 | 0.02 | 0.02 |

^1^ Reads were searched by BlastX against the *nr* protein database with minimum alignment length of 45 bp (15 amino acids) and minimum identity cutoff of 60%.

^2^ Other include bacterial phyla or proteobacterial classes contributing <0.1% reads in all the ten metagenomic datasets.

**Supplementary Table 36.** The contingency table that was used to determine by Chi-square test whether individual COG-counts under different metabolic/functional categories^1^ across the eight communities were significantly high or low.

| **Functional categories** | **VWM** | **DG3** | **DG4** | **WG3** | **WG4** | **WG5** | **WG6** | **WG7** |
| --- | --- | --- | --- | --- | --- | --- | --- | --- |
| **J** | 7.7 | 7.4 | 7.0 | 7.8 | 8.9 | 7.0 | 7.6 | 7.7 |
| **K** | 4.1 | 4.0 | 4.5 | 3.6 | 4.1 | 4.0 | 4.3 | 4.7 |
| **L** | 10.1 | 8.3 | 7.6 | 10.3 | 7.7 | 7.8 | 9.9 | 7.8 |
| **D** | 1.4 | 1.2 | 1.2 | 1.5 | 1.4 | 1.3 | 1.3 | 1.4 |
| **V** | 2.9 | 2.8 | 2.9 | 2.6 | 2.3 | 2.9 | 3.1 | 2.8 |
| **T** | 7.3 | 9.8 | 10.3 | 7.2 | 7.5 | 10.8 | 7.8 | 8.3 |
| **M** | 9.6 | 10.2 | 10.2 | 9.6 | 10.5 | 9.9 | 9.5 | 10.2 |
| **N** | 1.0 | 0.6 | 0.6 | 0.9 | 1.5 | 0.4 | 0.7 | 1.0 |
| **U** | 1.9 | 1.8 | 1.7 | 1.9 | 2.0 | 1.7 | 2.1 | 2.1 |
| **O** | 5.9 | 6.2 | 6.1 | 6.2 | 6.4 | 6.1 | 6.0 | 6.3 |
| **C** | 9.5 | 9.1 | 8.7 | 10.0 | 9.6 | 8.9 | 8.5 | 9.4 |
| **G** | 7.4 | 7.5 | 7.3 | 7.1 | 7.5 | 8.1 | 7.1 | 6.9 |
| **E** | 10.3 | 10.0 | 10.1 | 10.3 | 9.9 | 10.0 | 10.5 | 9.8 |
| **F** | 3.9 | 3.6 | 3.3 | 3.9 | 3.8 | 3.4 | 3.5 | 3.5 |
| **H** | 4.6 | 4.9 | 4.5 | 4.9 | 4.8 | 4.6 | 4.2 | 4.4 |
| **I** | 3.7 | 3.7 | 3.8 | 3.6 | 3.7 | 3.4 | 3.8 | 3.8 |
| **P** | 7.1 | 7.1 | 8.0 | 7.1 | 6.7 | 7.4 | 7.7 | 7.9 |
| **Q** | 1.7 | 1.9 | 2.3 | 1.6 | 1.5 | 2.1 | 2.2 | 2.0 |

^1^ **One-letter codes used to indicate the various metabolic/functional categories:J**, Translation; ribosomal structure and biogenesis; **K**, Transcription; **L**, Replication; recombination and repair; **D**, Cell cycle control; cell division; chromosome partitioning; **V**, Defense mechanisms; **T**, Signal transduction mechanisms; **M**, Cell wall/membrane/envelope biogenesis; **N**, Cell motility; **U**, Intracellular trafficking; secretion; and vesicular transport; **O**, Posttranslational modification; protein turnover; chaperones; **C**, Energy production and conversion; **G**, Carbohydrate transport and metabolism; **E**, Amino acid transport and metabolism; **F**, Nucleotide transport and metabolism; **H**, Coenzyme transport and metabolism; **I**, Lipid transport and metabolism; **P**, Inorganic ion transport and metabolism; **Q**, Secondary metabolites biosynthesis; transport and catabolism.

**Supplementary References**

**Supplementary references used in Table 2 of the main text and the Supplementary Table 33.**

Alain K, Postec A, Grinsard E, Lesongeur F, Prieur D, Godfroy A. *Thermodesulfatator atlanticus* sp. nov., a thermophilic, chemolithoautotrophic, sulfate-reducing bacterium isolated from a Mid-Atlantic Ridge hydrothermal vent. Int J Syst Evol Microbiol. 2010;60:33-38.

Kumar P, Srinivas TN, Thiel V, Tank M, Sasikala C, Ramana Ch V,et al. *Thiohalocapsa marina* sp. nov., from an Indian marine aquaculture pond. Int J Syst Evol Microbiol. 2009;59:2333-2338.

Bernardet JF, Bowman JP. Flavobacterium. *Bergey's Manual of Systematics of Archaea and Bacteria*. John Wiley and Sons: Chichester, UK. 2015.

Briand JF, Leboulanger C, Humbert JF, Bernard C, Dufour P. *Cylindrospermopsis raciborskii* (cyanobacteria) invasion at mid-latitudes: selection, wide physiological tolerance, orglobalwarming. J Phycol. 2004;40:231-238.

Brinkhoff T, Muyzer G, Wirsen CO, Kuever J. *Thiomicrospira chilensis* sp. nov., a mesophilic obligately chemolithoautotrophic sulfuroxidizing bacterium isolated from a Thioploca mat. Int J Syst Bacteriol. 1999;49:875-879.

Castenholz RW. Phylum BX. Cyanobacteria. In: Boone DR, Castenholz RW (eds). Bergey's Manual of Systematic Bacteriology. Springer: New York. 2001;473-599.

Chang YH, Han JI, Chun J, Lee KC, Rhee MS, Kim YB, et al. *Comamonas koreensis* sp. nov., a non-motile species from wetland in Woopo, Korea. Int J Syst Evol Microbiol. 2002;52:377-381.

Chen MY, Lin GH, Lin YT, Tsay SS. *Meiothermus taiwanensis* sp. nov., a novel filamentous, thermophilic species isolated in Taiwan. Int J Syst Evol Microbiol. 2002;52:1647-1654.

Christensen P. Description and Taxonomic Status of *Cytophaga heparina* (Payza and Korn) comb. nov. (Basionym: Flavobacterium heparinurn Payza and Korn 1956). Int J Syst Bacteriol.1980;30:473-475.

Clavel T, Duck W, Charrier C, Wenning M, Elson C, Haller D. *Enterorhabdus caecimuris* sp. nov., a member of the family Coriobacteriaceae isolated from a mouse model of spontaneous colitis, and emended description of the genus *Enterorhabdus* Clavel *et al*. 2009. Int J Syst Evol Microbiol. 2010;60:1527-1531.

Evans NJ, Brown JM, Demirkan I, Murray RD, Birtles RJ, Hart CA, et al. *Treponema pedis* sp. nov., a spirochaete isolated from bovine digital dermatitis lesions. Int J Syst Evol Microbiol. 2009;59:987-991.

Gotz D, Banta A, Beveridge TJ, Rushdi AI, Simoneit BR, Reysenbach AL. *Persephonella marina* gen. nov., sp. nov. and *Persephonella guaymasensis* sp. nov., two novel, thermophilic, hydrogen-oxidizing microaerophiles from deep-sea hydrothermal vents. Int J Syst Evol Microbiol. 2002;52:1349-1359.

Han X, Zheng J, Xin D, Xin Y, Wei X, Zhang J. Streptomyces albiflavescens sp. nov., an actinomycete isolated from soil. Int J Syst Evol Microbiol. 2015;65:1467-1473.

Hatamoto M, Kaneshige M, Nakamura A, Yamaguchi T. *Bacteroides luti* sp. nov., an anaerobic, cellulolytic and xylanolytic bacterium isolated from methanogenic sludge. Int J Syst Evol Microbiol. 2014;64:1770-1774.

Hirschler-Rea A, Matheron R, Riffaud C, Moune S, Eatock C, Herbert RA,et al. Isolation and characterization of spirilloid purple phototrophic bacteria forming red layers in microbial mats of Mediterranean salterns: description of Halorhodospira neutriphila sp. nov. and emendation of the genus Halorhodospira. Int J Syst Evol Microbiol. 2003;53:153-163.

Hosoya S, Yokota A. Reclassification of *Flexibacter aggregans* (Lewin 1969) Leadbetter 1974 as a later heterotypic synonym of Flexithrix dorotheae Lewin 1970. Int J Syst Evol Microbiol. 2007;57:1086-1088.

Huber R, Langworthy T, König H, Thomm M, Woese C, Sleytr U,et al*.Thermotoga maritima* sp. nov. represents a new genus of unique extremely thermophilic eubacteria growing up to 90°C. Arch Microbiol. 1986;144:324-333.

Imelfort M, Parks D, Woodcroft BJ, Dennis P, Hugenholtz P, Tyson GW. GroopM: an automated tool for the recovery of population genomes from related metagenomes. Peer J. 2014;2:e603.

Imhoff JF, Suling J, Petri R. Phylogenetic relationships among the Chromatiaceae, their taxonomic reclassification and description of the new genera *Allochromatium*, *Halochromatium*, *Isochromatium*, *Marichromatium*, *Thiococcus*, *Thiohalocapsa* and *Thermochromatium*. Int J Syst Bacteriol. 1998;48:1129-1143.

Jeanthon C, L'Haridon S, Cueff V, Banta A, Reysenbach AL, Prieur D. *Thermodesulfobacteriumhydrogeniphilum* sp. nov., a thermophilic, chemolithoautotrophic, sulfate-reducing bacterium isolated from a deep-sea hydrothermal vent at Guaymas Basin, and emendation of the genus Thermodesulfobacterium. Int J Syst Evol Microbiol. 2002;52:765-772.

Kang DD, Froula J, Egan R, Wang Z. MetaBAT, an efficient tool for accurately reconstructing single genomes from complex microbial communities. PeerJ. 2015;3:e1165.

Kelly DP, Wood AP. Reclassification of some species of *Thiobacillus* to the newly designated genera *Acidithiobacillus* gen. nov., *Halothiobacillus* gen. nov. and *Thermithiobacillus* gen. nov. Int J Syst Evol Microbiol. 2000;50:511-516.

Kim YO, Kong HJ, Park S, Kang SJ, Kim KK, Moon DY,et al*.Paracoccusfistulariae* sp. nov., a lipolytic bacterium isolated from bluespotted cornetfish, Fistularia commersonii. Int J Syst Evol Microbiol. 2010;60:2908-2912.

Koussemon M, Combet-Blanc Y, Patel BK, Cayol JL, Thomas P, Garcia JL,et al. *Propionibacteriummicroaerophilum* sp. nov., a microaerophilic bacterium isolated from olive mill wastewater. Int J Syst Evol Microbiol. 2001;51:1373-1382.

Koziel M, O'Doherty P, Vandamme P, Corcoran GD, Sleator RD, Lucey B. *Campylobactercorcagiensis* sp. nov., isolated from faeces of captive lion-tailed macaques (Macaca silenus). Int J Syst Evol Microbiol. 2014;64:2878-2883.

Li H, Durbin R. Fast and accurate short read alignment with Burrows-Wheeler transform. Bioinformatics (Oxford, England) . 2009;25:1754-1760.

Li H, Handsaker B, Wysoker A, Fennell T, Ruan J, Homer N, et al. The Sequence Alignment/Map format and SAMtools. Bioinformatics (Oxford, England). 2009;25:2078-2079.

Lien T, Madsen M, Rainey FA, Birkeland NK. *Petrotogamobilis* sp. nov., from a North Sea oil-production well. Int J Syst Bacteriol. 1998;48:1007-1013.

Mori K, Iino T, Ishibashi J, Kimura H, Hamada M, Suzuki K. *Meiothermushypogaeus* sp. nov., a moderately thermophilic bacterium isolated from a hot spring. Int J Syst Evol Microbiol. 2012;62:112-117.

Moussard H, L'Haridon S, Tindall BJ, Banta A, Schumann P, Stackebrandt E,et al. *Thermodesulfatatorindicus* gen. nov., sp. nov., a novel thermophilic chemolithoautotrophic sulfate-reducing bacterium isolated from the Central Indian Ridge. Int J Syst Evol Microbiol. 2004;54:227-233.

Nakagawa S, Takai K, Horikoshi K, Sako Y. *Persephonellahydrogeniphila* sp. nov., a novel thermophilic, hydrogen-oxidizing bacterium from a deep-sea hydrothermal vent chimney. Int J Syst Evol Microbiol. 2003;53:863-869.

Parks DH, Imelfort M, Skennerton CT, Hugenholtz P, Tyson GW. CheckM: assessing the quality of microbial genomes recovered from isolates, single cells, and metagenomes. Genome Res. 2015;25:1043-1055.

Podosokorskaya OA, Kublanov IV, Reysenbach AL, Kolganova TV, Bonch-Osmolovskaya EA. *Thermosiphoaffectus* sp. nov., a thermophilic, anaerobic, cellulolytic bacterium isolated from a Mid-Atlantic Ridge hydrothermal vent. Int J Syst Evol Microbiol. 2011;61:1160-1164.

Rautio M, Eerola E, Vaisanen-Tunkelrott ML, Molitoris D, Lawson P, Collins MD,et al. Reclassification of *Bacteroidesputredinis* (Weinberg *et al*., 1937) in a new genus *Alistipes* gen. nov., as *Alistipesputredinis* comb. nov., and description of *Alistipesfinegoldii* sp. nov., from human sources. Syst Appl Microbiol. 2003;26:182-188.

Rooney AP, Dunlap CA, Flor-Weiler LB. *Acinetobacter lactucae sp*. nov., isolated from iceberg lettuce (Asteraceae: Lactuca sativa). Int J Syst Evol Microbiol. 2016;66:3566-3572.

Saiki T, Kobayashi Y, Kawagoe K, Beppu T. *Dictyoglomusthermophilum* gen. nov., sp. nov., a Chemoorganotrophic, Anaerobic, Thermophilic Bacterium. Int J Syst Bacteriol. 1985;35:253-259.

Schleifer KH, Schüler D, Spring S, Weizenegger M, Amann R, Ludwig W,et al. The Genus *Magnetospirillum* gen. nov. Description of *Magnetospirillumgryphiswaldense* sp. nov. and Transfer of Aquaspirillum magnetotacticum to Magnetospirillum magnetotacticum comb. nov. Syst Appl Microbiol. 1991;14:379-385.

Shah HN, Collins MD. Proposal for Reclassification of *Bacteroidesasaccharolyticus*, Bacteroides gingivalis, and Bacteroides endodontalis in a New Genus, Porphyromonas. Int J Syst Evol Microbiol. 1988;38:128-131.

Sherman JM, Stark P. Streptococci which grow at high temperatures. J. Bacteriol. 1931;22:275-285.

Shida O, Takagi H, Kadowaki K, Nakamura LK, Komagata K. Transfer of *Bacillusalginolyticus*, *Bacilluschondroitinus*, *Bacilluscurdlanolyticus*, *Bacillusglucanolyticus*, *Bacilluskobensis*, and *Bacillusthiaminolyticus* to the genus *Paenibacillus* and emended description of the genus Paenibacillus. Int J Syst Bacteriol. 1997;47:289-98.

Smythe L, Adler B, Hartskeerl RA, Galloway RL, Turenne CY, Levett PN,et al. Classification of *Leptospiragenomospecies* 1, 3, 4 and 5 as *Leptospiraalstonii* sp. nov., *Leptospiravanthielii* sp. nov., *Leptospiraterpstrae* sp. nov. and *Leptospirayanagawae* sp. nov., respectively. Int J Syst Evol Microbiol. 2013;63:1859-1862.

Sorokin DY, Tourova TP, Muyzer G, Kuenen GJ. *Thiohalospirahalophila* gen. nov., sp. nov. and *Thiohalospiraalkaliphila* sp. nov., novel obligately chemolithoautotrophic, halophilic, sulfur-oxidizing gammaproteobacteria from hypersaline habitats. Int J Syst Evol Microbiol. 2008;58:1685-1692.

Stohr R, Waberski A, Volker H, Tindall BJ, Thomm M. *Hydrogenothermusmarinus* gen. nov., sp. nov., a novel thermophilic hydrogen-oxidizing bacterium, recognition of *Calderobacteriumhydrogenophilum* as a member of the genus *Hydrogenobacter* and proposal of the reclassification of *Hydrogenobacteracidophilus* as *Hydrogenobaculumacidophilum* gen. nov., comb. nov., in the phylum 'Hydrogenobacter/Aquifex'. Int J Syst Evol Microbiol. 2001;51:1853-1862.

Subhash Y, Sasikala C, Ramana Ch V. *Hymenobacterroseus* sp. nov., isolated from sand. Int J Syst Evol Microbiol. 2014;64:4129-4133.

Sucharita K, Kumar ES, Sasikala C, Panda BB, Takaichi S, Ramana Ch V. *Marichromatiumfluminis* sp. nov., a slightly alkaliphilic, phototrophic gammaproteobacterium isolated from river sediment. Int J Syst Evol Microbiol. 2010;60:1103-1107.

Sun W, Dong GX, Zhang YQ, Wei YZ, Li QP, Yu LY,et al. *Actinoplanes* sichuanensis sp. nov. and Actinoplanes xinjiangensis sp. nov. Int J Syst Evol Microbiol. 2009;59:2763-2768.

Takai K, Kobayashi H, Nealson KH, Horikoshi K. Sulfurihydrogenibium subterraneum gen. nov., sp. nov., from a subsurface hot aquifer. Int J Syst Evol Microbiol. 2003;53:823-827.

Tully JG, Whitcomb RF, Rose DL, Bove JM, Carle P, Somerson NL,et al. *Acholeplasmabrassicae* sp. nov. and *Acholeplasmapalmae* sp. nov., two non-sterol-requiring mollicutes from plant surfaces. Int J Syst Bacteriol. 1994;44:680-684.

Urios L, Cueff-Gauchard V, Pignet P, Postec A, Fardeau ML, Ollivier B,et al. *Thermosiphoatlanticus* sp. nov., a novel member of the *Thermotogalesisolated* from a Mid-Atlantic Ridge hydrothermal vent. Int J Syst Evol Microbiol. 2004;54:1953-1957.

Vajna B, Kanizsai S, Keki Z, Marialigeti K, Schumann P, Toth EM. *Thermuscomposti* sp. nov., isolated from oyster mushroom compost. Int J Syst Evol Microbiol. 2012;62:1486-1490.

Wang MQ, Sun L. *Pseudomonas oceani* sp. nov., isolated from deep seawater. Int J Syst Evol Microbiol. 2016;66:4250-4255.

Wells JM, Raju BC, Hung HY, Weisburg WG, Mandelco-Paul L, Brenner DJ. *Xylellafastidiosa* gen. nov., sp. nov: Gram-Negative, Xylem-Limited, Fastidious Plant Bacteria Related to Xanthomonas spp. Int J Syst Evol Microbiol. 1987;37:136-143.

Wolfgang WJ, Carpenter AN, Cole JA, Gronow S, Habura A, Jose S,et al. *Neisseriawadsworthii* sp. nov. and *Neisseriashayeganii* sp. nov., isolated from clinical specimens. Int J Syst Evol Microbiol. 2011;61:91-98.

Wood AP, Kelly DP, McDonald IR, Jordan SL, Morgan TD, Khan S,et al. A novel pink-pigmented facultative methylotroph, *Methylobacteriumthiocyanatum* sp. nov., capable of growth on thiocyanate or cyanate as sole nitrogen sources. Arch Microbiol. 1998;169:148-158.

Zhang Z, Wang Y, Ruan J. A proposal to revive the genus *Kitasatospora* (Omura, Takahashi, Iwai, and Tanaka 1982). Int J Syst Bacteriol. 1997;47:1048-1054.
